# A Metataxonomic Approach Reveals Diversified Bacterial Communities in Antarctic Sponges

**DOI:** 10.3390/md19030173

**Published:** 2021-03-22

**Authors:** Nadia Ruocco, Roberta Esposito, Marco Bertolino, Gianluca Zazo, Michele Sonnessa, Federico Andreani, Daniela Coppola, Daniela Giordano, Genoveffa Nuzzo, Chiara Lauritano, Angelo Fontana, Adrianna Ianora, Cinzia Verde, Maria Costantini

**Affiliations:** 1 Department of Marine Biotechnology, Stazione Zoologica Anton Dohrn, Villa Comunale, 80121 Napoli, Italy; nadia.ruocco@szn.it (N.R.); roberta.esposito@szn.it (R.E.); daniela.coppola@ibbr.cnr.it (D.C.); daniela.giordano@ibbr.cnr.it (D.G.); chiara.lauritano@szn.it (C.L.); ianora@szn.it (A.I.); cinzia.verde@ibbr.cnr.it (C.V.); 2 Department of Biology, University of Naples Federico II, Complesso Universitario di Monte Sant’Angelo, Via Cinthia 21, 80126 Napoli, Italy; 3 Dipartimento di Scienze della Terra, dell’Ambiente e della Vita (DISTAV), Università degli Studi di Genova, Corso Europa 26, 16132 Genova, Italy; marco.bertolino@unige.it; 4 Department of Research Infrastructure for Marine Biological Resources, Stazione Zoologica Anton Dohrn, Villa Comunale, 80121 Napoli, Italy; gianluca.zazo@szn.it; 5 Bio-Fab Research srl, Via Mario Beltrami, 5, 00135 Roma, Italy; laboratori@biofabresearch.it (M.S.); michelesonnessa@biofabresearch.it (F.A.); 6 Institute of Biosciences and BioResources (IBBR), National Research Council (CNR), Via Pietro Castellino 111, 80131 Napoli, Italy; 7 Consiglio Nazionale delle Ricerche, Istituto di Chimica Biomolecolare, Via Campi Flegrei 34, 80078 Pozzuoli (Napoli), Italy; nuzzo.genoveffa@icb.cnr.it (G.N.); afontana@icb.cnr.it (A.F.)

**Keywords:** Antarctica, Demospongiae, marine biotechnology, metataxonomics, microbiota

## Abstract

Marine sponges commonly host a repertoire of bacterial-associated organisms, which significantly contribute to their health and survival by producing several anti-predatory molecules. Many of these compounds are produced by sponge-associated bacteria and represent an incredible source of novel bioactive metabolites with biotechnological relevance. Although most investigations are focused on tropical and temperate species, to date, few studies have described the composition of microbiota hosted by Antarctic sponges and the secondary metabolites that they produce. The investigation was conducted on four sponges collected from two different sites in the framework of the XXXIV Italian National Antarctic Research Program (PNRA) in November–December 2018. Collected species were characterized as *Mycale* (*Oxymycale*) *acerata*, *Haliclona* (*Rhizoniera*) *dancoi, Hemigellius pilosus* and *Microxina sarai* by morphological analysis of spicules and amplification of four molecular markers. Metataxonomic analysis of these four Antarctic sponges revealed a considerable abundance of Amplicon Sequence Variants (ASVs) belonging to the phyla Proteobacteria, Bacteroidetes, Actinobacteria and Verrucomicrobia. In particular, *M*. (*Oxymycale*) *acerata,* displayed several genera of great interest, such as *Endozoicomonas*, *Rubritalea*, *Ulvibacter*, *Fulvivirga* and *Colwellia*. On the other hand, the sponges *H. pilosus* and *H.* (*Rhizoniera*) *dancoi* hosted bacteria belonging to the genera *Pseudhongella*, *Roseobacter* and *Bdellovibrio*, whereas *M. sarai* was the sole species showing some strains affiliated to the genus *Polaribacter*. Considering that most of the bacteria identified in the present study are known to produce valuable secondary metabolites, the four Antarctic sponges could be proposed as potential tools for the discovery of novel pharmacologically active compounds.

## 1. Introduction

The Antarctic region comprises ice shelves, waters and all the island territories in the Southern Ocean, covering about 10% of the total world ocean’s area. The Antarctic is characterized by low temperature and scarce availability of nutrients, together with a high seasonality in terms of light conditions. Due to the extreme environmental conditions, Antarctic fauna has developed several physiological and behavioural adaptations, leading to the evolution of unique characteristics [1]. For instance, a longer period of larval development or parental care has been observed in Antarctic invertebrates, including sponges [2,3,4]. Moreover, marine invertebrate communities living in this area have been subjected to a wide temporal and biogeographic isolation [5,6] dating back to about 140 million years ago when the Antarctic continent separated from Gondwana [7,8]. This event has promoted the development of specific traits, which make Antarctic organisms extremely diverse from those living in other southern hemisphere seas [9,10,11].

Sponges are sessile and filter-feeder organisms, belonging to the phylum Porifera, which represent, in terms of abundance and biomass, the major component of the Antarctic zoobenthos [12], with a total number of 400 known species [13]. Through their aquiferous system, they are able to capture several microorganisms (including bacteria, yeasts, microalgae) from surrounding water and harbour a huge microbial community within their body [14,15,16]. Sponges normally establish a strong interaction with their bacterial hosts due to several benefits that improve their fitness and survival, including nutritional supply, transport of waste products, and molecules that confer chemical and mechanical defence [17,18,19].

Although a good knowledge is available on sponge fauna, the Antarctic region covers an extraordinarily wide area that makes some zones almost unknown to the scientific community [20,21]. Until now, a few studies have investigated the composition of microbial communities living within Antarctic sponges [22,23,24,25,26,27,28,29,30]. Some of these studies have demonstrated that Antarctic sponges are mostly dominated by Proteobacteria and Bacteroidetes [23,26,29,31]. Interestingly, species composition has been found to be strictly specific, probably regulated by several bioactive molecules and quorum sensing [14,31,32,33,34,35]. Since several studies have revealed that symbiotic bacteria are able to produce bioactive metabolites (reviewed by Brinkmann et al. [36]), studying the species composition of microbiomes could shed light on the possible biotechnological applications of sponges. This scientific question becomes much more attractive when addressed to Antarctic species, which are still largely unknown, and might reserve a great potential considering that they have undergone incredible adaptations. Interactions between sponges and microorganisms may occur in many forms, representing these microorganisms’ food sources, pathogens/parasites, or mutualistic symbionts [37,38,39,40]. Microbial associates can represent up to 40% of sponge tissue volume. Furthermore, the diversity in types of interactions may be matched by the phylogenetic diversity of microbes that occur within host sponges.

In the present work, we aimed to enlarge the yet scant knowledge on the bacterial communities inhabiting Antarctic sponges. In particular, we collected four sponges from two different sites of Tethys Bay (Victoria Land, Antarctica) in the framework of the XXXIV Italian National Antarctic Research Program (PNRA) expedition. Victoria Land (Tethys Bay) belongs to the Antarctic Specially Protected Area n. 161 [41] (ASPA 161; https://www.ats.aq/devAS/Meetings/Measure/688 accessed on 29 January 2021). Sponge species were characterized by morphological and molecular analysis. Metagenomic DNA extraction and Illumina MiSeq analysis were applied in order to identify the associated communities living within the analysed sponges. More than five hundred bacterial isolates were phylogenetically identified to establish whether the associated bacterial communities were host-specific. By relying on Amplicon Sequence Variants (ASVs) data, the biotechnological potential of sponge specimens was also considered.

## 2. Results

### 2.1. Species Identification 

#### 2.1.1. Morphological Analysis

The four sponge specimens belonged to the class Demospongiae and the following two orders: Haplosclerida with three species, *Hemigellius pilosus* (Kirkpatrick, 1907), *Microxina sarai* (Calcinai and Pansini, 2000), and *Haliclona* (*Rhizoniera*) *dancoi* (Topsent, 1901), and Poecilosclerida with one species, *Mycale* (*Oxymycale*) *acerata* (Kirkpatrick, 1907) (Table 1).

#### 2.1.2. Molecular Analysis

BLAST similarity search totally agreed with the morphological identification obtained for B4 and D4 samples. Molecular analysis confirmed B4 species as *M.* (*Oxymycale*) *acerata*, with CO1 primers that were the most specific (98% of pairwise identity) in comparison to 18S, 28S and ITS molecular markers. Similarly, CO1 also appeared to be the best molecular marker for the identification of the sponge D4, with a highest sequence similarity to *H. pilosus* (98% sequence identity). Regarding sample C6, molecular markers identified the genus corresponding to *Haliclona*, with the most striking result achieved using the 18S marker (92% similarity to *Haliclona sp*.). Unfortunately, it was not possible to identify this sponge at the species level, because there are no other available sequences on GenBank for *H.* (*Rhizoniera*) *dancoi*. Similarly, the results achieved with sample D6 were partially unclear, since several genera at low-sequence similarity were observed from BLAST outputs. In fact, the sequences of *M. sarai*, identified by spicule observations, are still not uploaded in GenBank (see Tables S1–S4; details on the alignments are reported in Figures S1–S3).

### 2.2. Metataxonomic Data Analysis

**Figure 1:**
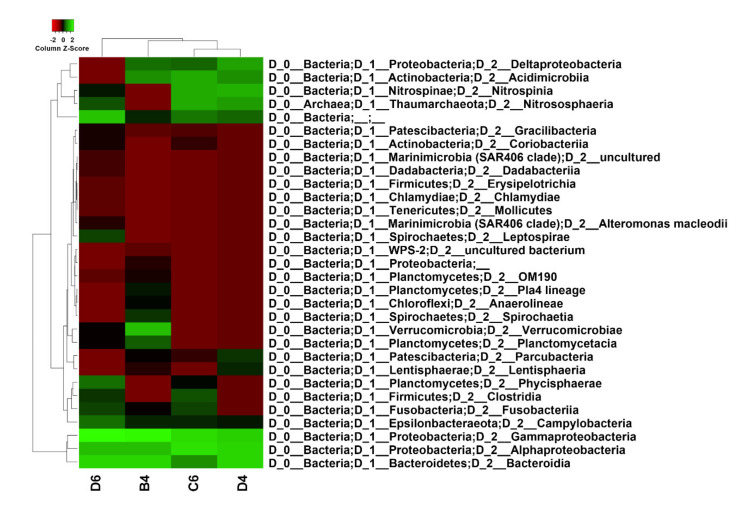
Heatmap of taxon relative abundance using taxonomic profiling, showing that sponge samples were all dominated by Gammaproteobacteria, Alphaproteobacteria and Bacteroidia. Sample code: B4 = *M.* (*Oxymycale*) *acerata*; D4 = *H. pilosus*, D6 = *M. sarai*, C6 = *H.* (*Rhizoniera*) *dancoi*. Scaling was done by column and clustering was performed using average linkage method and Manhattan distance measurement. Values were normalized as Log10.

ASVs analysis was conducted considering those reporting a percentage of confidence ≥ 75%. The sponge *M.* (*Oxymycale*) *acerata* (B4) hosted the greatest abundance of bacterial taxa (250 ASVs), while *H. pilosus* (D4), *H.* (*Rhizoniera*) *dancoi* (C6) and *M. sarai* (D6) showed 47, 55 and 120 ASVs, respectively (Tables S5–S8). Overall, concerning the taxonomic profiling, sponge samples were all dominated by Gammaproteobacteria, Alphaproteobacteria and Bacteroidia (Figure 1).

In addition, *M.* (*Oxymycale*) *acerata*, *H. pilosus* and *H.* (*Rhizoniera*) *dancoi* revealed an abundance of both Deltaproteobacteria and Acidimicrobiia. Manhattan algorithm indicated that *M. sarai* clustered separately in comparison to the others, with *H. pilosus* and *H.* (*Rhizoniera*) *dancoi* resulting as the most similar in terms of species structure and abundance (Figure 1).

**Figure 2:**
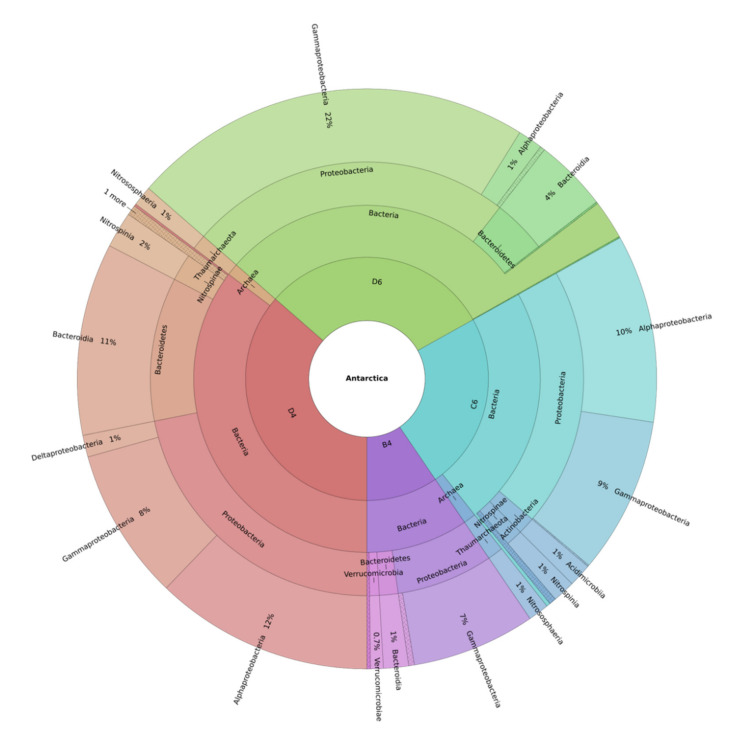
Krona Plot at the class level. Gammaproteobacteria of the genus Endozoicomonas were identified from *M.* (*Oxymycale*) *acerata* as well as Gammaproteobacteria belonging to the genus *Colwellia* and some bacterial strains classified as *Fulvivirga* and *Ulvibacter*, two genera included into Bacteroidetes. Bacteria of the family Rhodobacteraceae (class Alphaproteobacteria) were identified in *H.* (*Rhizoniera*) *dancoi* and *H. pilosus*. *M. sarai* was the only species showing a relative abundance of Polaribacter, an additional species grouped into the Bacteroidetes phylum. Sample code: B4 = *M.* (*Oxymycale*) *acerata*; D4 = *H. pilosus*, D6 = *M. sarai*, C6 = *H.* (*Rhizoniera*) *dancoi*. “1 more” corresponds to Acidicrodobiia group, which is present in trace levels.

More specifically, a high relative frequency of Gammaproteobacteria and Bacteroidia were found in *M.* (*Oxymycale*) *acerata* (61.5% and 19%, respectively) and *M. sarai* (71% and 14%, respectively) (Figure 2; see also Figure S4).

On the contrary, a lower percentage (5–7%) of Alphaproteobacteria was detected in both species. In addition, *M.* (*Oxymycale*) *acerata* revealed 2–7% of bacteria belonging to Acidimicrobiia and Verrucomicrobiae classes, while lower percentages (~1%) of other bacterial phyla (Proteobacteria, Epsilonbacteraeota, Planctomycetes) together with 7% of an unknown phylum were recorded in *M. sarai* (Figure S4).

As reported above (Figure 1), the sponges *H.* (*Rhizoniera*) *dancoi* and *H. pilosus* revealed a similar composition in bacterial species distribution. In fact, a high abundance of Alphaproteobacteria (44% in *H.* (*Rhizoniera*) *dancoi* and 33.2% in *H. pilosus*) and Gammaproteobacteria (37% in *H.* (*Rhizoniera*) *dancoi* and 24% *H. pilosus*) was observed in both species. Moreover, lower percentages (0.5–5%) of additional taxa were recorded, including the Nitrospinia, Nitrososphaeria, Acidimicrobiia and Deltaproteobacteria groups. A huge difference was detected for bacteria belonging to the class Bacteroidia, since a higher relative abundance was found in *H. pilosus* (14%) in comparison to *H.* (*Rhizoniera*) *dancoi* (2%) (Figure S4).

## 3. Discussion

In the present study, we analyzed the species composition and abundance of the associated microbiota from four Antarctic sponges, *M.* (*Oxymycale*) *acerata*, *H.* (*Rhizoniera*) *dancoi*, *H. pilosus* and *M. sarai*, collected from Tethys Bay (Victoria Land, Antarctica). In particular, the associated community of *M.* (*Oxymycale*) *acerata*, collected from site 1 (Table 1), was similar to *H.* (*Rhizoniera*) *dancoi* and *H. pilosus* (Figure 1), retrieved from site 2 (Table 1). Interestingly, at this latter site, we collected the sponge *M. sarai*, whose species abundance was found statistically different by Manhattan clustering analysis (Figure 1).

As reported in most investigations focusing on sponge-associated bacteria [42,43,44,45,46,47], taxonomic profiling showed that Proteobacteria and Bacteroidetes dominated the four Antarctic sponges (Figure 2; Tables S5–S8). Previous studies identified these bacterial groups from *M.* (*Oxymycale*) *acerata* and other Antarctic species by metagenomic approaches [23,27,28,30,31,32,48]. These bacteria were frequently found to be the dominant bacterial phyla in marine ecosystems [49]. In particular, Proteobacteria showed different functions in host, including nitrogen fixation, and were involved in host defense mechanisms [50]. Furthermore, some bacteria were described as highly specialized hydrocarbon degrading microorganisms [51,52] and their wide distribution may be due to a strong positive interaction in environments where bacteria represent a fundamental source of nutrients, such as the case of Antarctica. This finding could be corroborated by results revealing that these bacteria are able to adapt to extreme environments, including polar habitats [53,54,55,56]. Concerning their biotechnological potential, genome-mining approaches reported several biosynthetic gene clusters (BGCs) encoding for bioactive molecules from marine Proteobacteria (reviewed by Buijs et al. [57]). However, there is no direct 100% correlation between the presence of a certain BGC, a bacterial genus and a bioactive metabolite. BGCs can be silent in certain conditions and, hence, methods should be developed to unlock their silent potential [58], to observe the production of a particular compound and induce the desired bioactivity. The most common approach known to discover new metabolites is the “OSMAC” (one strain many compounds) approach. The term OSMAC was coined for the first time by Zeeck and co-workers [59], indicating the ability of single strains to produce different metabolites when cultivated under different conditions. Examples are the use of different culturing strategies to trigger the production of secondary metabolites such as changing culturing conditions (e.g., nutrients or light exposure), mimicking environmental stressors and co-culturing with other species.

On the whole, several species belonging to Gammaproteobacteria and Alphaproteobacteria isolated from sponges and soils showed antibacterial, antiviral, antifungal and antiprotozoal activities that make them suitable tools in drug discovery research fields [36,43,60,61,62,63]. In particular, the Gammaproteobacteria of the genus *Endozoicomonas*, identified from *M.* (*Oxymycale*) *acerata* in the present work (Figure 2; Table S5), was found to induce antimicrobial activities [64,65].

Always *M.* (*Oxymycale*) *acerata* showed a relative abundance of a Gammaproteobacteria belonging to the genus *Colwellia* (Figure 2; Table S5), which is extremely interesting since it was recently proposed as a useful tool for the bioremediation of nitrogen pollutants [66]. Previous investigations also demonstrated that a sponge-associated *Colwellia* sp. produces several extracellular polymeric substances (EPSs) with potential use in the production of cosmeceutical and nutraceutical ingredients [35,67].

*M.* (*Oxymycale*) *acerata* also revealed some bacterial strains classified as *Fulvivirga* and *Ulvibacter*, two genera included into Bacteroidetes, the second most abundant phylum found in the samples under analysis (Figure 2; Table S5). Genome-mining approaches coupled to chemical analyses revealed the presence of some amine acylated desferrioxamine siderophores from *Fulvivirga* sp. with anticancer properties [68]. Similarly, *Ulvibacter* species, already observed in Antarctic habitats [69], belong to the family Flavobacteriaceae, whose biotechnological applications are well-documented. In fact, several polysaccharide-digesting enzymes together with antibiotics and other bioactive compounds, such as quercetin (known for its antioxidant, anti-inflammatory, chemopreventive properties), were isolated [70,71].

The sequencing of 16S regions revealed that *M.* (*Oxymycale*) *acerata* was the sole species hosting a certain abundance of *Rubritalea* strains (phylum Verrucomicrobia) (Figure 2; Table S5). This bacterial group was already observed in other sponge species, from which some BGCs encoding for PKSs (polyketide synthases) were identified [42,72,73,74,75,76,77]. Verrucomicrobia, coupled with Planctomycetes and Chlamydiae, was classified in the PVC (Planctomycetes, Verrucomicrobia, and Chlamydiae) superphylum, which is known to include a wide number of species with biotechnological potential [78,79,80]. The finding of these bacteria within the symbiotic community of *M.* (*Oxymycale*) *acerata* may be extremely attractive since several bioactive molecules, such as carotenoids and squalene, were found in several bacteria belonging to the genus *Rubritalea* [72,73,74,81,82,83,84]. The potential capability to produce biotechnologically relevant compounds was also demonstrated by genomic analyses, revealing some genes involved in defense mechanisms mediated by toxin-antitoxin systems from sponge-associated verrucomicrobial strains [77].

ASV’s data showed bacteria of the family Rhodobacteraceae (class Alphaproteobacteria) in *H.* (*Rhizoniera*) *dancoi* and *H. pilosus* (Figure 2; Tables S6 and S7). Several genera were recognized as a huge source of novel bioactives, especially *Pseudovibrio* species living in seawater and through symbiotic relationships with sponges, tunicates and corals [85,86]. For example, *H. pilosus* specifically hosted the genus *Roseobacter*, which was also studied for its antimicrobial properties [87,88]. The hydrocarbon-degrading Gammaproteobacteria of the genus *Pseudohongiella*, with potential use in the bioremediation of anthropogenic contaminants [89,90], were also revealed in *H.* (*Rhizoniera*) *dancoi* and *H. pilosus* (Figure 2; Tables S6 and S7).

Less abundant members living within *H. pilosus* and *H.* (*Rhizoniera*) *dancoi* belonged to the classes Nitrospinia (phylum Nitrospinae) and Nitrosophaeria (phylum Thaumarchaeota) (Figure 2; Tables S6 and S7). Recent investigations already reported low percentages of Nitrospinia from *H. pilosus*, *H.* (*Rhizoniera*) *dancoi* and other Antarctic species [26,29]. Concerning the capability to produce molecules with biotechnological potential, very little information is available so far. In a recent study, BLASTp search against the Integrated Microbial Genomes (IMG) database identified a *Pseudoalteromonas luteoviolacea* gene encoding for a l-amino acid oxidase (LAAO) with antimicrobial properties in the genome of a strain belonging to the phylum Nitrospinae [91].

The sponges under analysis had low percentages of Acidimicrobiia (phylum Actinobacteria), except for *M. sarai* (Tables S5–S8). According to our results, this bacterial class was recently reported from *H.* (*Rhizoniera*) *dancoi*, *H. pilosus* and other Antarctic sponges by metagenomic analysis [27,28]. Acidimicrobiia were widely observed in marine sponges, particularly from tropical species [63,92,93,94,95,96,97]. Similar to Proteobacteria, several studies demonstrated the great biotechnological potential of Actinobacteria, especially those belonging to the *Streptomyces* genus. In fact, several bioactive compounds with antimicrobial, antiviral, antiparasitic, antiprotozoal and antitumor effects have been described [98,99,100,101,102,103,104,105,106,107]. Moreover, genomic analyses revealed some BGCs encoding for secondary metabolites, such as PKS I and III, NRPS (nonribosomal peptides), terpene and bacteriocin gene clusters from a sponge-derived Actinobacteria showing antimicrobial activities [108,109,110,111,112].

**Figure 3:**
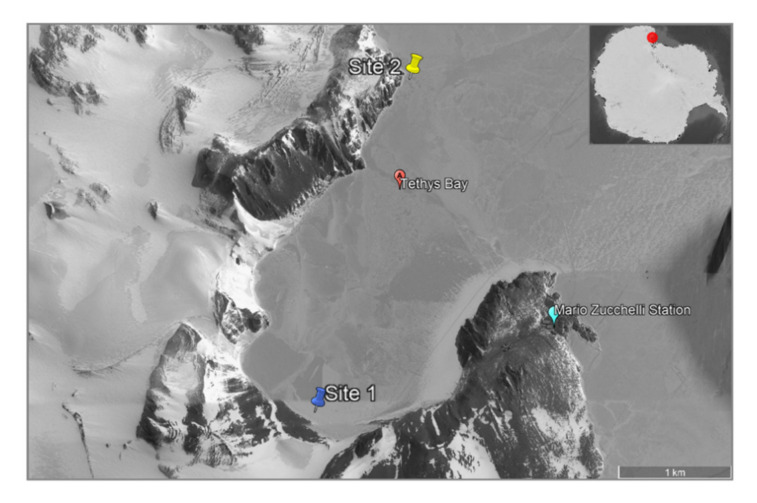
Map of Tethys Bay (Victoria Land, Antarctica). The collection sites were reported as blue (site 1) and yellow (site 2) icons. Scale bar = 1 km.

ASV’s analysis of the three sponges, *M.* (*Oxymycale*) *acerata*, *H. pilosus* and *H.* (*Rhizoniera*) *dancoi*, displayed Deltaproteobacteria (Figure 2; Tables S5–S7) belonging to the phylum Proteobacteria, that, as mentioned above, produce interesting bioactive metabolites [57]. For instance, *H. pilosus* exhibited some strains included into the genus *Bdellovibrio* (Figure 3; Table S7), which is an obligate predator of other Gram-negative bacteria that was proposed for possible biotechnological applications toward medicinal, agricultural and industrial fields [113,114,115].

*M. sarai* was the only species showing a relative abundance of *Polaribacter*, an additional species grouped into the Bacteroidetes phylum (Figure 2; Table S8). Some data demonstrated that these cold-adapted bacteria produced interesting EPSs molecules with protective effects on human skin and anti-aging properties [116,117].

## 4. Materials and Methods

### 4.1. Sponge Collection

Four sponge samples, reported as B4, C6, D4 and D6, were collected by scuba divers in November–December 2018 at two sites of Tethys Bay: 1) B4 at 26 metres of depth (74°42.067′ S, 164°02.518′ E) and 2) C6, D4 and D6 at 28 metres of depth (74°40.537′ S, 164°04.169′ E) (Figure 3). Samples were immediately washed at least three times with filter-sterilized natural seawater to remove transient and loosely attached bacteria and/or debris [14,27]. Firstly, a small fragment of each sponge was preserved in 70% ethanol for taxonomic identification. Specimens were then placed into individual sterile tubes and kept in RNA*later*^©^ at −20° C until transported to the Stazione Zoologica Anton Dohrn (Naples, Italy).

Sponge slides of spicules are deposited at the Italian National Antarctic Museum (MNA, Section of Genoa, Italy). The MNA voucher codes of the sponges investigated in the present work are reported in Table 1.

### 4.2. Morphological Analysis of Spicules 

The taxonomic identification was conducted at the species level. Small fragments of each sponge were heat-dissolved in nitric acid, rinsed in water and dehydrated in ethanol. Then, spicules were mounted on slides for microscopic analyses, following standard methods [118]. The skeletal architecture was examined under light microscope and hand-cut sections of sponge portions were made as described in Hooper [119].

The taxonomic classification follows the updated nomenclature reported in the World Porifera Database (WPD) [120].

### 4.3. DNA Extraction and PCR Amplification

About 10 mg of tissue were used for DNA extraction by using *QIAamp^®^ DNA Micro kit* (QIAGEN), according to the manufacturer’s instructions. DNA quantity (ng/μL) was evaluated by NanoDrop spectrophotometer. PCR reactions were performed on the C1000 Touch Thermal Cycler (BioRad) in a 30 µL reaction mixture final volume including about 50–100 ng of genomic DNA, 6 µL of 5X Buffer GL (GeneSpin Srl, Milan, Italy), 3 µL of dNTPs (2 mM each), 2 µL of each forward and reverse primer (25 pmol/µL, Table 1), 0.2 µL of Xtra Taq Polymerase (5 U/µL, GeneSpin Srl, Milan, Italy), using different PCR programs for 18S and 28S, ITS and CO1 as follows:

(i) for 18S and 28S, a denaturation step at 95 °C for 2 min, [35 cycles denaturation step at 95 °C for 1 min, annealing step at 60 °C (A/B, [121]), 57 °C (C2/D2, [122]), 55 °C (18S-AF/18S-BR, NL4F/NL4R, [123]) for 1 min and 72 °C of primer extension for 2 min], a final extension step at 72 °C for 10 min;

(ii) ITS primers (RA2/ITS2.2, [121]), a first denaturation at 95 °C for 2 min, [35 cycles denaturation step at 95 °C for 1 min, annealing step at 67 °C for 1 min and 72 °C of primer extension for 2 min], a final extension step at 72 °C for 10 min. 

(iii) CO1 primers (dgLCO1490/dgHCO2198, [124]), a first denaturation at 94 °C for 3 min, [35 cycles of denaturation at 94 °C for 30 sec, annealing at 45 °C for 30 sec and primer extension at 72 °C for 1 min].

Sequences of PCR primers are reported in Supporting Information (Table S9). PCR products were run on 1.5% agarose gel and the fragment length was evaluated by using 100 bp DNA ladder (GeneSpin Srl, Milan, Italy). PCR products were purified using *QIAquick Gel Extraction Kit* (Qiagen), according to the manufacturer’s instructions. PCR amplicons were then sequenced in both strands through Applied Biosystems (Life Technologies) 3730 Analyzer (48 capillaries). Sequences produced were ~300–650 bases long in average with more than 97.5% accuracy, starting from PCR fragments. The total 18S, 28S, ITS and CO1 region were submitted to GenBank using Basic Local Alignment Search Tool (BLAST) [125] and then aligned with highly similar sequences using MultiAlin (http://multalin.toulouse.inra.fr/multalin/ accessed on 29 January 2021) [126].

### 4.4. Metagenomic DNA Extraction and Illumina MiSeq Sequencing

Genomic DNA for 16S rRNA sequencing was performed from about 250 mg of tissue by using *DNeasy^®^ PowerSoil^®^ Pro Kit* (QIAGEN), according to the manufacturer’s instructions. Extractions were performed using both internal and external sponge tissue in order to obtain the whole bacterial community. DNA quantity (ng/μL) and quality (A260/280, A260/230) were evaluated by NanoDrop spectrophotometer, whereas DNA integrity was checked on 0.8% agarose gel electrophoresis in TAE buffer (40 mM Tris-acetate, 1 mM EDTA, pH 8.0). 20 µL of samples (30 ng/μL final concentration) were subjected to 16S V3-V4 rRNA gene library preparation and sequencing (Bio-Fab Research, Rome, Italy). Illumina adapters overhang nucleotide sequences were added to the gene specific primer sequences targeting the V3-V4 region [127]. After 16S amplification, a PCR clean-up was done to purify the V3-V4 amplicon from free primers and primer-dimer species. A subsequent limited cycle amplification step was performed to add multiplexing indices and Illumina sequencing adapters by using a Nextera XT Index Kit. Finally, libraries were normalized and pooled by denoising processes (Table S10), and sequenced on Illumina MiSeq Platform with 2x300 bp paired-end reads. Taxonomy was assigned using “home made” Naive Bayesian Classifier trained on V3-V4 16S sequences of SILVA 132 database [128]. QIIME 2 (Quantitative Insights Into Microbial Ecology) platform [129] was used for microbiome analysis from raw DNA sequencing data. QIIME analysis workflow was performed by demultiplexing, quality filtering, chimera removal and taxonomic assignment. The full dataset of raw data has been deposited in the SRA database (submission ID: SUB8701897; BioProject ID: PRJNA687362).

### 4.5. Statistical Analysis

ASVs distribution and frequency in the whole dataset and for each sample are reported in the Supporting Information (Figures S5 and S6).

Heatmap was generated by using Heatmapper Software available at http://www.heatmapper.ca/ accessed on 29 January 2021 [130]. The number of features observed for each identified taxa were normalized as Log10 and scaled by column. Hierarchical clustering was applied on both rows and columns by average linkage method. For computing distance between rows and columns, Manhattan distance measurement algorithm was performed.

## 5. Conclusions

Our metataxonomic analysis highlights the occurrence of dominant and locally enriched microbes in the Antarctic sponges *M.* (*Oxymycale*) *acerata*, *H.* (*Rhizoniera*) *dancoi*, *H. pilosus* and *M. sarai*, characterized by morphological and molecular approaches. This can be considered a starting point in the understanding of the global Antarctic microbiome in a more complete perspective, given the scarce information in the literature for extreme environments such as the Antarctica. According to the microbial community identified, the biotechnological value should not be underestimated. In fact, our findings open new perspectives concerning the possible role of these Antarctic sponges and their symbiotic bacteria as a source of bioactive compounds. Further studies will be devoted to bioassay-guided fractionations for identifying new potential drugs useful in pharmaceutical, nutraceutical and cosmeceutical fields.

## Figures and Tables

**Table 1:** Sites, sample IDs, species identification, MNA code, geographic coordinates, sampling method in meters (m) and depth.

Site	Sample ID	Sponge Taxonomy	MNA code	Sampling Method	Sampling Depth (m)	Coordinates
1	B4	*Mycale* (*Oxymycale*) *acerata* (Kirkpatrick, 1907)	13264	SCUBA	26	74°42.067’S 164°02.518’E
2	C6	*Haliclona* (*Rhizoniera*) *dancoi* (Topsent, 1901)	13265	SCUBA	28	74°40.537’S 164°04.169’E
2	D4	*Hemigellius pilosus* (Kirkpatrick, 1907)	13266	SCUBA	28	74°40.537’S 164°04.169’E
2	D6	*Microxina sarai* (Calcinai & Pansini, 2000)	13267	SCUBA	28	74°40.537’S 164°04.169’E

## Supplementary Materials

The following are available online at https://www.mdpi.com/1660-3397/19/3/173/s1, Figure S1. Alignments of CO1 (PorCOI2fwd/PorCOI2rev) sequence from B4 sponge with (a) the first BLAST hit *Asbestopluma lycopodium* and (b) the sequence of *M. acerata* displaying low query cover, Figure S2: Alignment of CO1 (dgLCO1490/dgHCO2198) sequence from B4 sponge with the first BLAST hit (*M. acerata*), Figure S3: Alignment of CO1 (dgLCO1490/dgHCO2198) sequence from D4 sponge with the first BLAST hit (*H. pilosus*), Figure S4. Krona plot at increasing complexity levels. Regnum (a), phylum (b), class (c), order (d), family (e), genus (f) and species (g) were reported. Figure S5. Distribution of ASV’s frequencies, Figure S6. Distribution of ASV’s frequencies for each sample (reported as a blue bar), Table S1. BLAST results from B4 sponge (*Mycale* (*Oxymycale*) *acerata*). The primer names, sequence length in base pairs (bp), first hits (highlighted in bold), hits at low significance displaying the correct species (where present), query cover and identity percentages (%) were reported, Table S2. BLAST results from C6 sponge (*Haliclona* (*Rhizoniera*) *dancoi*). The primer names, sequence length in base pairs (bp), first hits (highlighted in bold), hits at low significance displaying the correct species (where present), query cover and identity percentages (%) were reported, Table S3. BLAST results from D4 sponge (*Hemigellius pilosus*). The primer names, sequence length in base pairs (bp), first hits (highlighted in bold), hits at low significance displaying the correct species (where present), query cover and identity percentages (%) were reported, Table S4. BLAST results from D6 sponge (*Microxina sarai*). The primer names, sequence length in base pairs (bp), first hits (highlighted in bold), query cover and identity percentages (%) were reported, Table S5. ASVs found from *M.* (*Oxymycale*) *acerata* with percentage of confidence ≥ 75%, Table S6. ASVs found from *H.* (*Rhizoniera*) *dancoi* with percentage of confidence ≥ 75%, Table S7. ASVs found from *H. pilosus* with percentage of confidence ≥ 75%, Table S8. ASVs found from *M. sarai* with percentage of confidence ≥ 75%, Table S9. Targeted region, forward and reverse names, sequences (5’→ 3’) and reference of primers pairs used for molecular characterization, Table S10. Denoising process.
