# Supplementary material for: A Metataxonomic Approach Reveals Diversified Bacterial Communities in Antarctic Sponges
Source: Mar Drugs. 2021 Mar 22;19(3):173. doi: 10.3390/md19030173 (PMC8004616; doi:10.3390/md19030173)
Supplement: Supplementary file 1 [file marinedrugs-19-00173-s001.pdf]

Supplementary Material

Figure S1. Alignments of CO1 (PorCOI2fwd/PorCOI2rev) sequence from B4 sponge with (A) the first BLAST hit *Asbestopluma lycopodium* and (B) the sequence of *M. acerata* displaying low query cover.

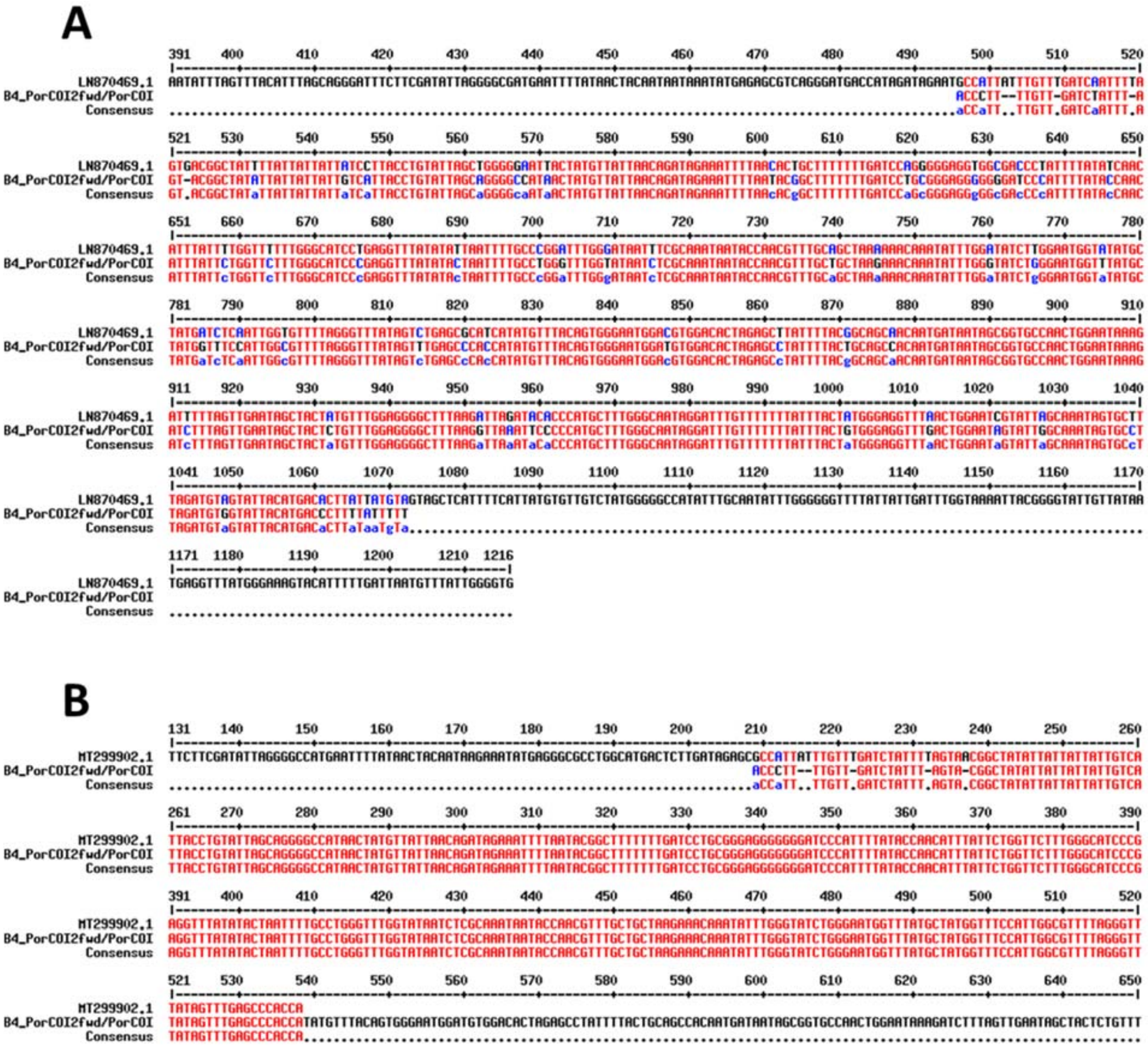

Figure S2. Alignment of CO1 (dgLCO1490/dgHCO2198) sequence from B4 sponge with the first BLAST hit (*M. acerata*).

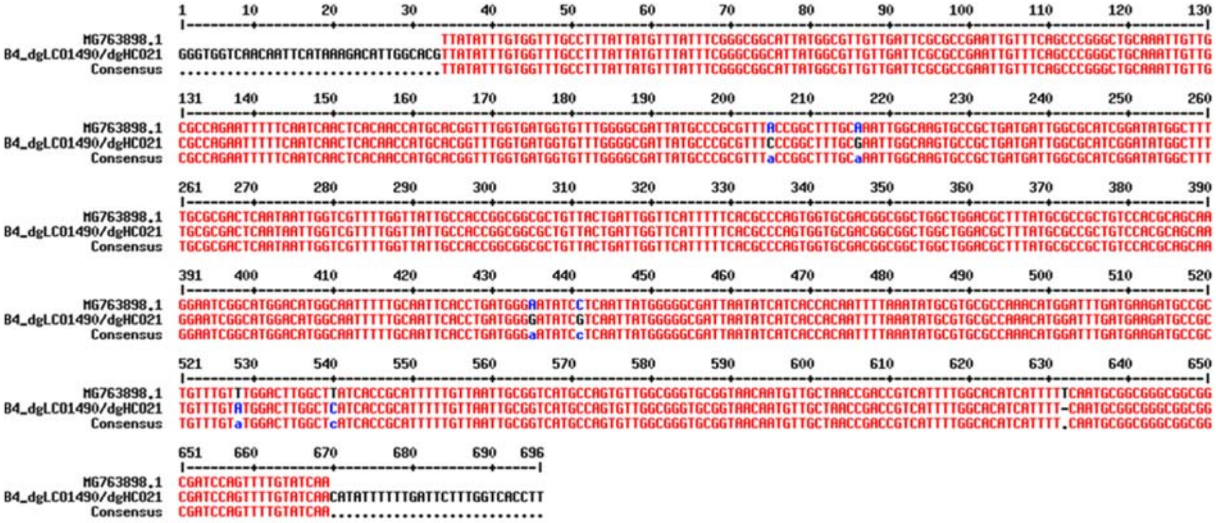

Figure S3. Alignment of CO1 (dgLCO1490/dgHCO2198) sequence from D4 sponge with the first BLAST hit (*H. pilosus*).

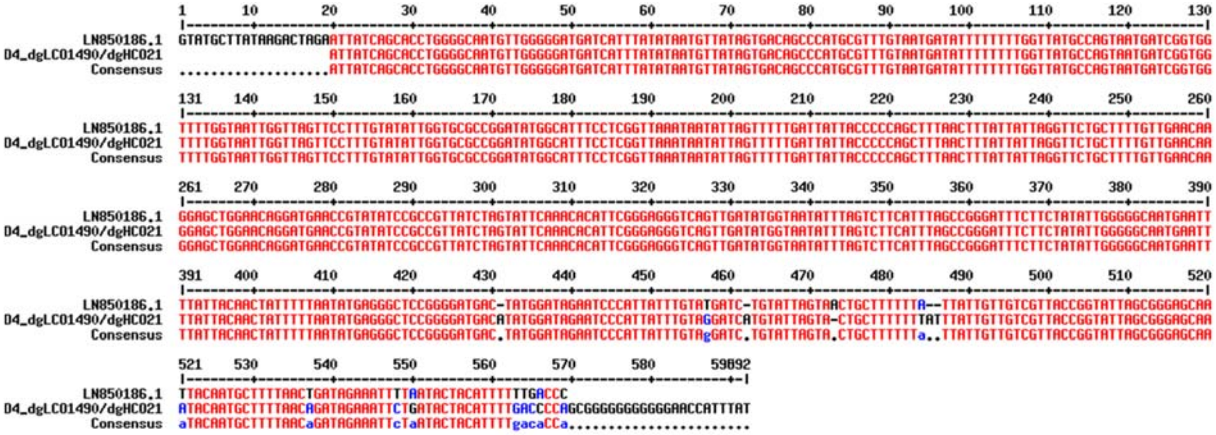

Figure S4. Taxonomy Bar Plot, reporting the relative frequencies (in percentage, %) of the bacteria taxons more representative for each of the four sponges under analysis . Sample code: B4= *M.* (*Oxymycale*) *acerata*; D4= *H. pilosus*, D6= *M. sarai*, C6= *H.* (*Rhizoniera*) *dancoi*. Each taxon is highlighted by a different color.

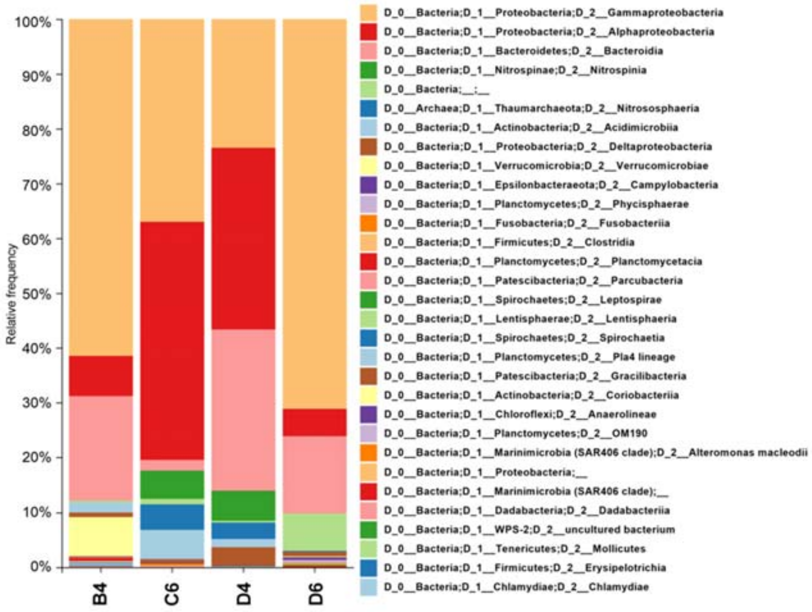

Figure S5. Krona plot at the seven increasing complexity levels: (a) Regnum, (b) Phylum, (c) Class, (d) Order, (e) Family, (f) Genus and (g) Species.

a)

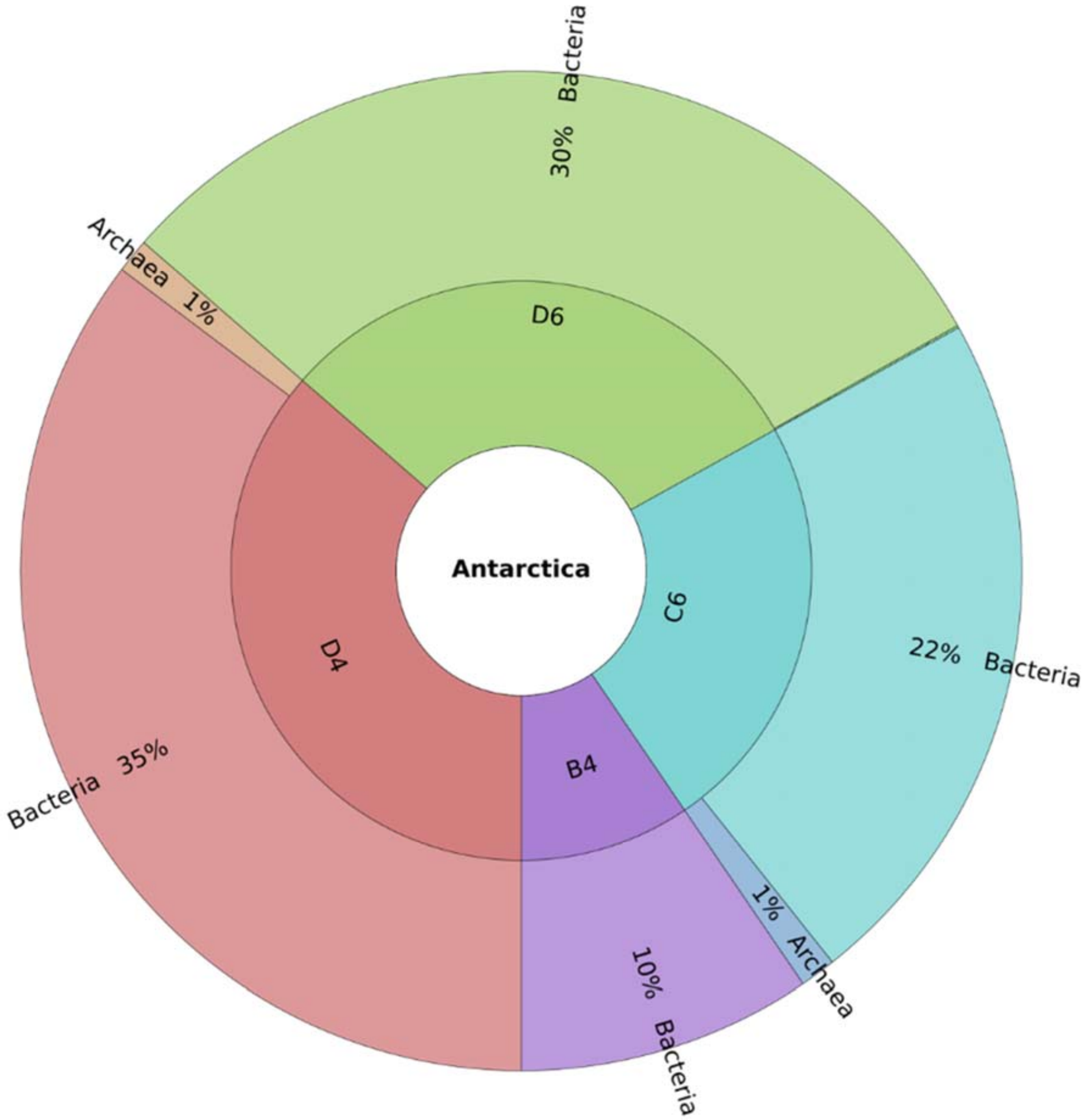

b)

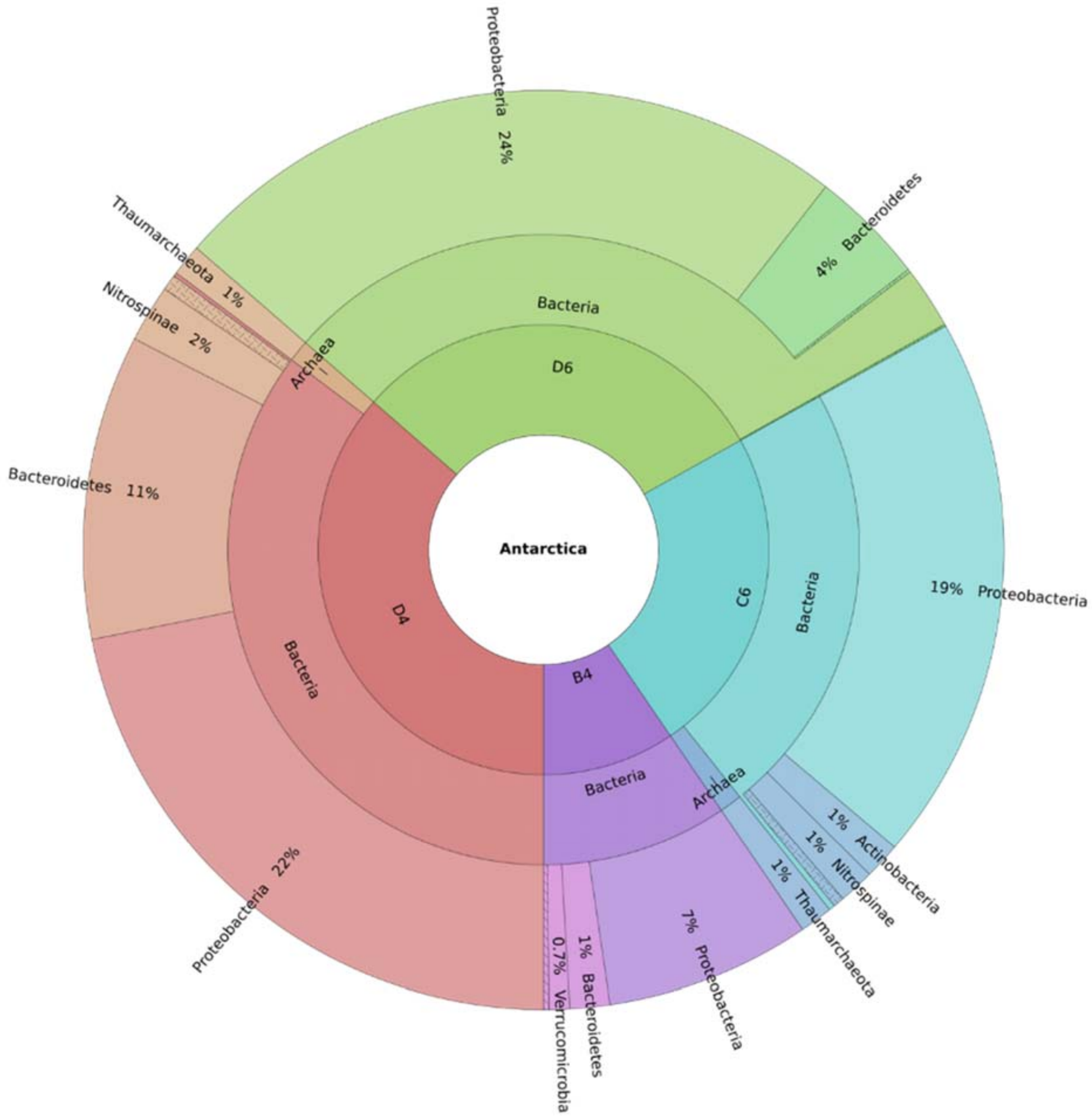
Antarctica
D6
Bacteria
Proteobacteria 24%
4% Bacteroidetes
Thaumarchaeota 1%
Nitrospinae 2%
Archaea
Bacteroidetes 11%
D4
Bacteria
Proteobacteria 22%
C6
Bacteria
19% Proteobacteria
B4
Bacteria
Archaea
1% Actinobacteria
1% Nitrospinae
1% Thaumarchaeota
7% Proteobacteria
1% Bacteroidetes
0.7% Verrucomicrobia

c)

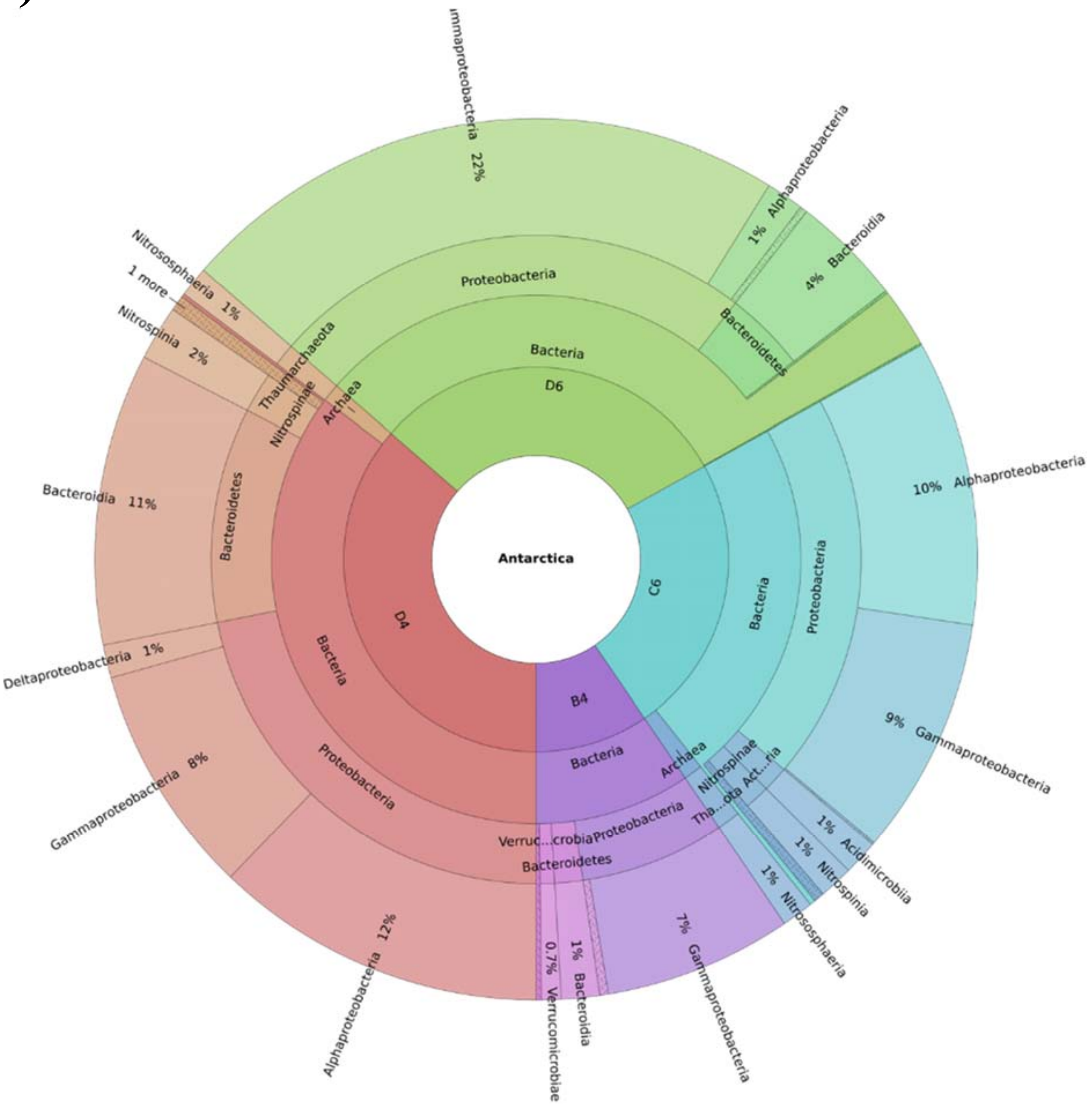
Antarctica
D6
Bacteria
Proteobacteria
Gammaproteobacteria 22%
1% Alphaproteobacteria
4% Bacteroidia
Bacteroidetes
Archaea
Thaumarchaeota
Nitrospinae
Nitrososphaeria 1%
1 more
Nitrospinia 2%
C6
Bacteria
Proteobacteria
10% Alphaproteobacteria
9% Gammaproteobacteria
D4
Bacteria
Bacteroidetes
Proteobacteria
Bacteroidia 11%
Deltaproteobacteria 1%
Gammaproteobacteria 8%
Alphaproteobacteria 12%
B4
Bacteria
Archaea
Nitrospinae
Tha...ota
Act...ria
Proteobacteria
Verruc...crobia
Bacteroidetes
1% Acidimicrobiia
1% Nitrospinia
1% Nitrososphaeria
7% Gammaproteobacteria
1% Bacteroidia
0.7% Verrucomicrobiae

d)

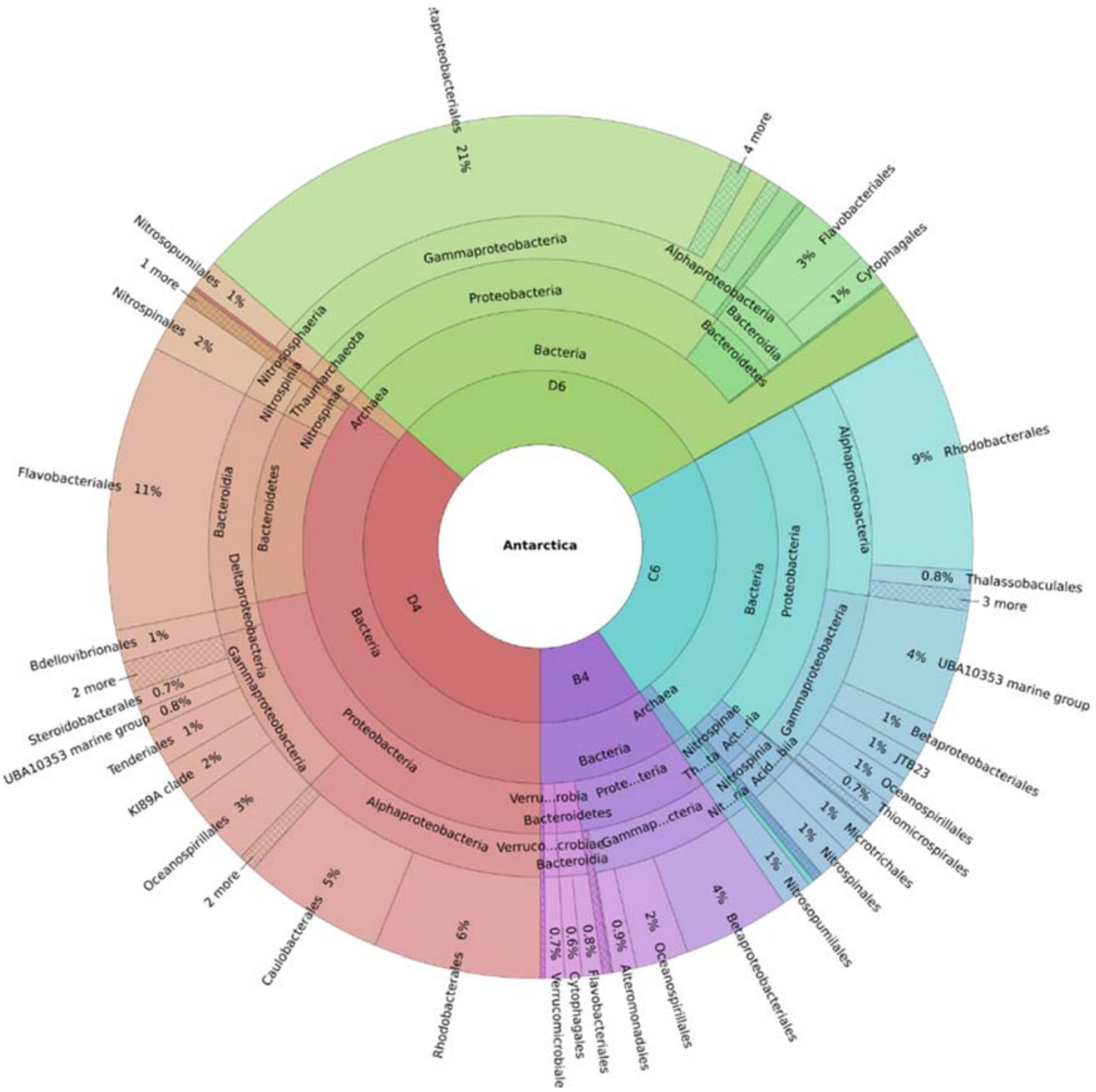
Antarctica
D6
Bacteria
Proteobacteria
Gammaproteobacteria
Alphaproteobacteria
Bacteroidia
Bacteroidetes
21%
4 more
3% Flavobacteriales
1% Cytophagales
Nitrosopumilales 1%
1 more
Nitrospinales 2%
Nitrososphaeria
Nitrospinia
Thaumarchaeota
Nitrospinae
Archaea
C6
9% Rhodobacterales
0.8% Thalassobaculales
3 more
4% UBA10353 marine group
1% Betaproteobacteriales
1% JTB23
1% Oceanospirillales
0.7% Thiomicrospirales
1% Microtrichales
1% Nitrospinales
1% Nitrosopumilales
D4
Flavobacteriales 11%
Deltaproteobacteria
Bdellovibrionales 1%
2 more
Steroidobacterales 0.7%
UBA10353 marine group 0.8%
Tenderiales 1%
KI89A clade 2%
Oceanospirillales 3%
2 more
Caulobacterales 5%
Rhodobacterales 6%
B4
Verru...robia
Prote...teria
Gammap...cteria
Verruco...crobiae
Th...ta
Act...ria
Nit...ria
Acid...bia
0.7% Verrucomicrobiale
0.6% Cytophagales
0.8% Flavobacteriales
0.9% Alteromonadales
2% Oceanospirillales
4% Betaproteobacteriales

e)

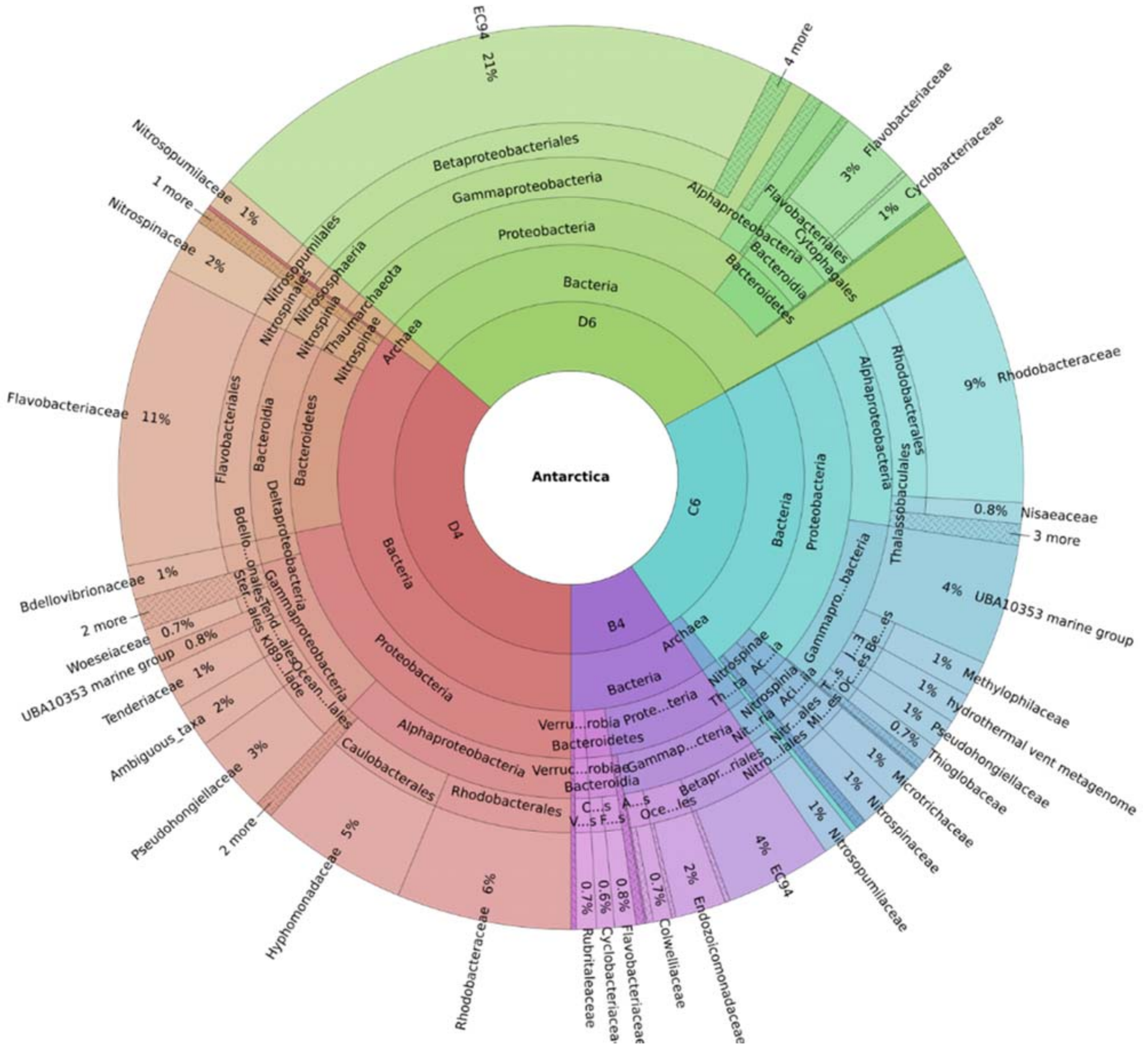

Antarctica
D6
D4
C6
B4
Bacteria
Archaea
Proteobacteria
Gammaproteobacteria
Betaproteobacteriales
EC94 21%
4 more
Flavobacteriaceae 3%
Cyclobacteriaceae 1%
Alphaproteobacteria
Flavobacteriales
Cytophagales
Bacteroidia
Bacteroidetes
Thaumarchaeota
Nitrososphaeria
Nitrosopumilales
Nitrosopumilaceae 1%
1 more
Nitrospinaceae 2%
Nitrospinia
Nitrospinales
Nitrospinae
Flavobacteriaceae 11%
Flavobacteriales
Deltaproteobacteria
Bdellovibrionaceae 1%
2 more
Woeseiaceae 0.7%
UBA10353 marine group 0.8%
Tenderiaceae 1%
Ambiguous_taxa 2%
Pseudohongiellaceae 3%
2 more
Hyphomonadaceae 5%
Caulobacterales
Rhodobacterales
Rhodobacteraceae 6%
Rhodobacteraceae 9%
Thalassobaculales
Nisaeaceae 0.8%
3 more
UBA10353 marine group 4%
Methylophilaceae 1%
hydrothermal vent metagenome 1%
Pseudohongiellaceae 1%
Thioglobaceae 0.7%
Microtrichaceae 1%
Nitrospinaceae 1%
Nitrosopumilaceae 1%
EC94 4%
Endozoicomonadaceae 2%
Colwelliaceae 0.7%
Flavobacteriaceae 0.8%
Cyclobacteriaceae 0.6%
Rubritaleaceae 0.7%
Verrucomicrobiae
Betaproteobacteriales

f)

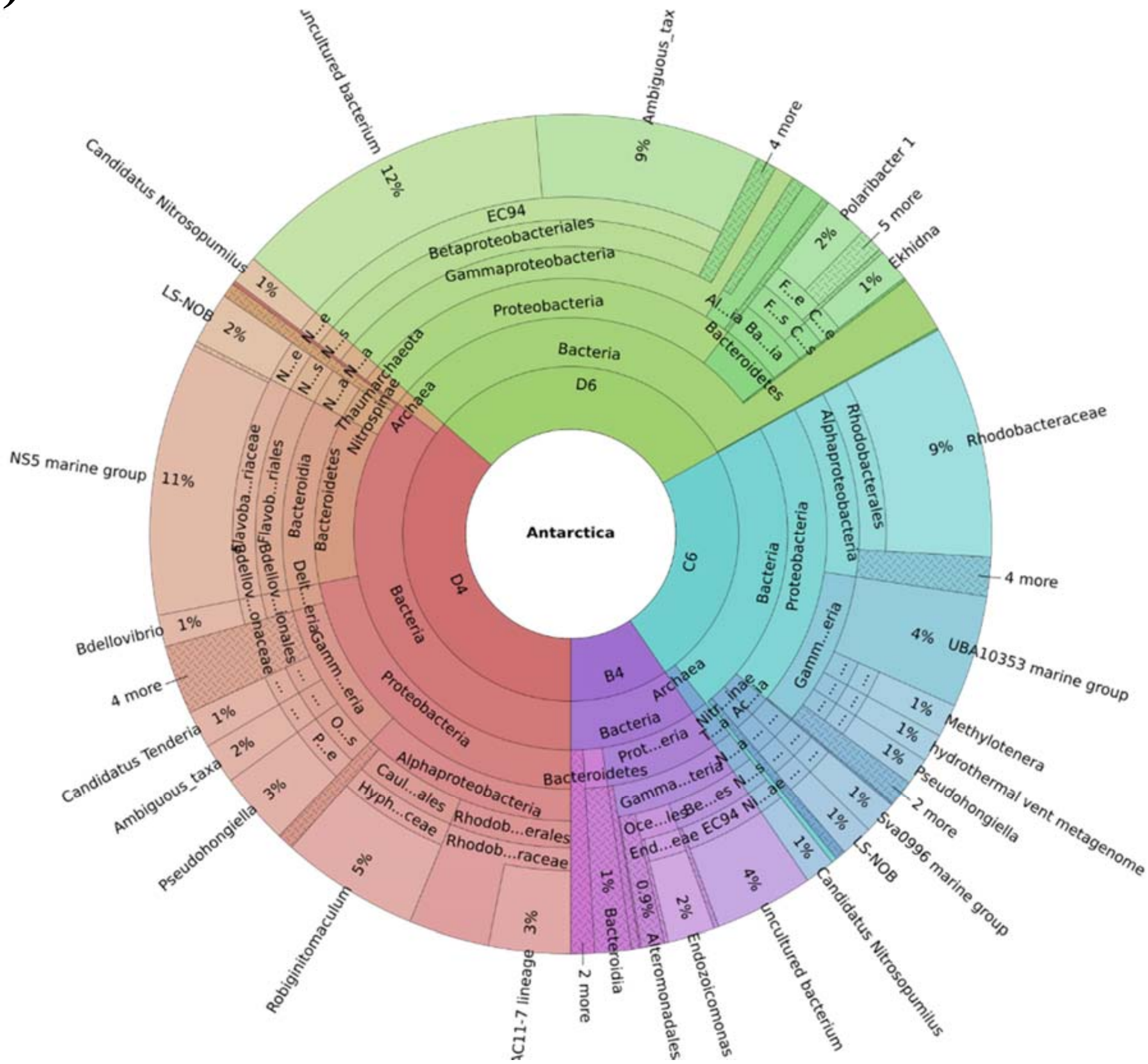
Antarctica
D6
D4
C6
B4
Bacteria
Archaea
Proteobacteria
Gammaproteobacteria
Betaproteobacteriales
EC94
Bacteroidetes
Thaumarchaeota
Nitrospinae
Bacteroidia
Alphaproteobacteria
Rhodobacterales
Rhodobacteraceae
uncultured bacterium
12%
Ambiguous_tax
9%
4 more
Polaribacter 1
5 more
Ekhidna
2%
1%
Candidatus Nitrosopumilus
LS-NOB
NS5 marine group
11%
Bdellovibrio
4 more
Candidatus Tenderia
Ambiguous_taxa
Pseudohongiella
3%
Robiginitomaculum
5%
AC11-7 lineage
2 more
Alteromonadales
Endozoicomonas
4%
0.9%
Rhodobacteraceae
9%
4 more
UBA10353 marine group
Methylotenera
hydrothermal vent metagenome
Pseudohongiella
2 more
Sva0996 marine group
LS-NOB
Candidatus Nitrosopumilus
uncultured bacterium
Caul...ales
Hyph...ceae
Rhodob...erales
Rhodob...raceae
Gamma...teria
Prot...eria
Oce...les
End...eae
EC94
Flavob...riales
Flavoba...riaceae
Bdellov...ionales
Bdellov...onaceae
Delt...eria
Gamm...eria

g)

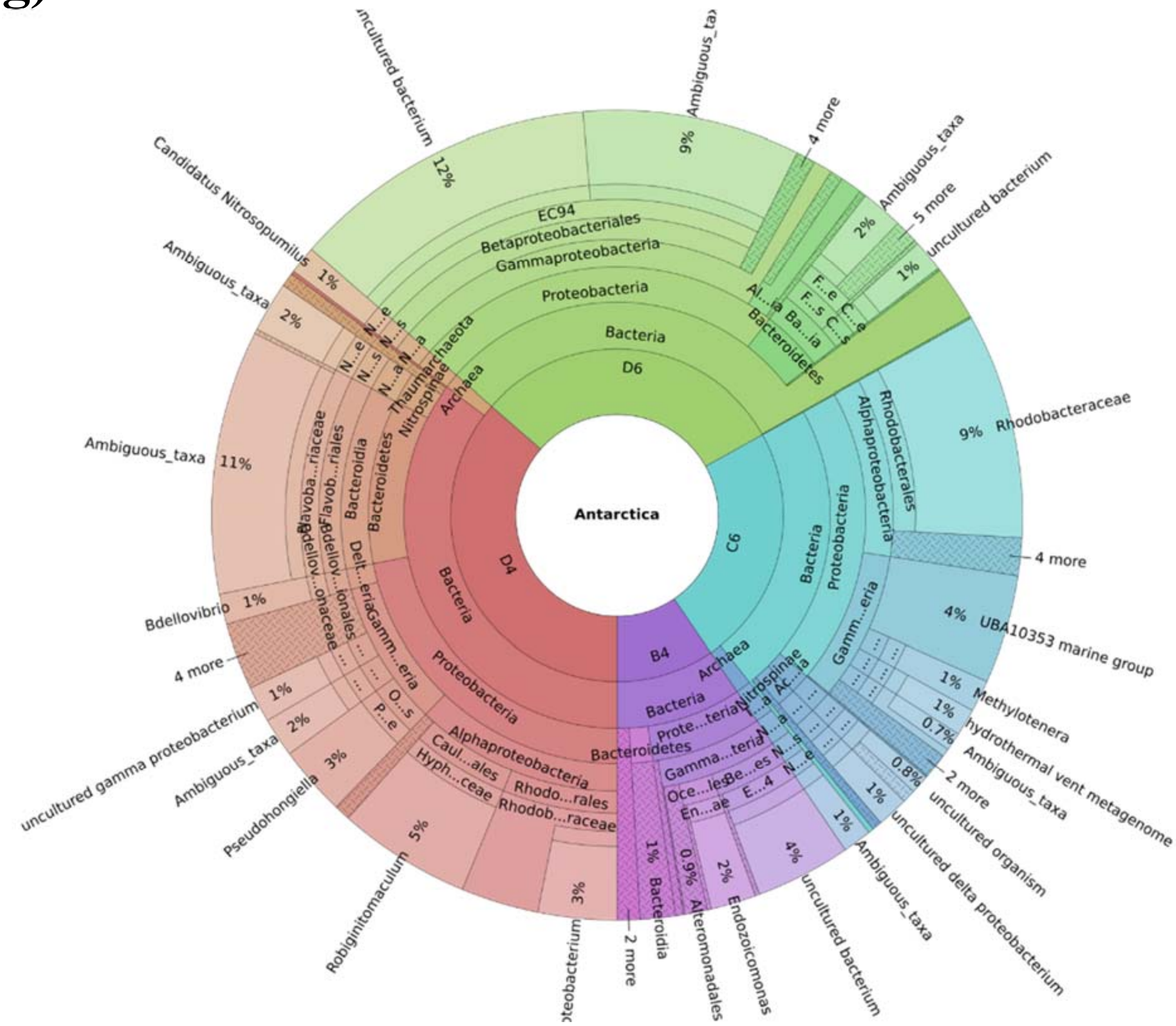

Antarctica
D6
C6
B4
D4
Bacteria
Archaea
Proteobacteria
Gammaproteobacteria
Betaproteobacteriales
EC94
Bacteroidetes
Thaumarchaeota
Nitrospinae
uncultured bacterium
12%
Ambiguous_taxa
9%
4 more
Ambiguous_taxa
2%
5 more
uncultured bacterium
1%
Rhodobacteraceae
9%
Rhodobacterales
Alphaproteobacteria
4 more
4% UBA10353 marine group
1% Methylotenera
1% hydrothermal vent metagenome
0.7% Ambiguous_taxa
2 more
0.8% uncultured organism
1% uncultured delta proteobacterium
1% Ambiguous_taxa
4% uncultured bacterium
2% Endozoicomonas
0.9% Alteromonadales
1% Bacteroidia
2 more
3%
Rhodob...raceae
Rhodo...rales
Alphaproteobacteria
Caul...ales
Hyph...ceae
5% Robiginitomaculum
3% Pseudohongiella
2% Ambiguous_taxa
1% uncultured gamma proteobacterium
4 more
1% Bdellovibrio
11% Ambiguous_taxa
2% Ambiguous_taxa
1% Candidatus Nitrosopumilus
Bacteroidia
Flavob...riales
Flavoba...riaceae
Bdellov...ionales
Bdellov...ohnaceae
Delt...eria
Gamm...eria
Gamma...teria
Prote...teria

Figure S6. Distribution of ASV's frequencies.

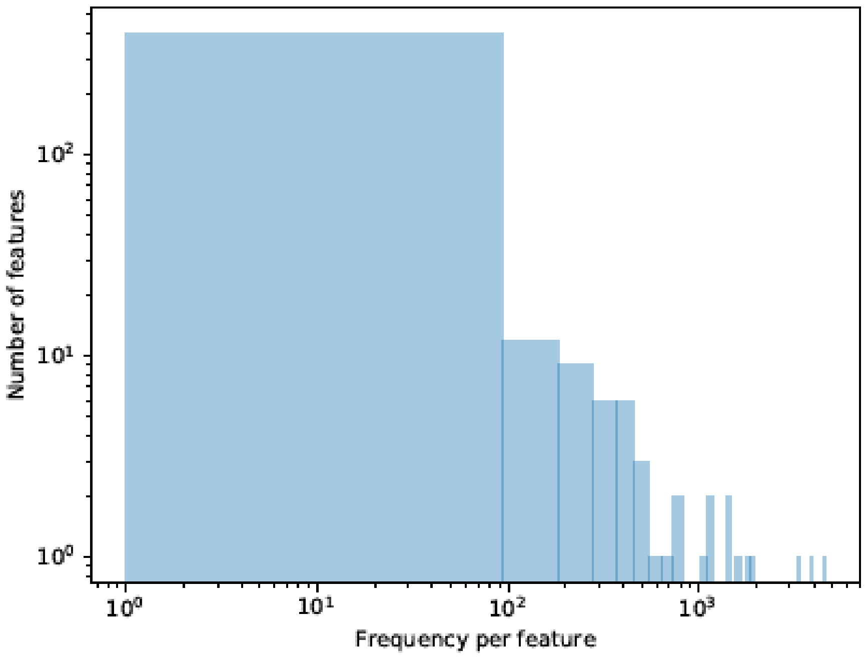

Figure S7. Distribution of ASV's frequencies for each sample (reported as a blue bar).

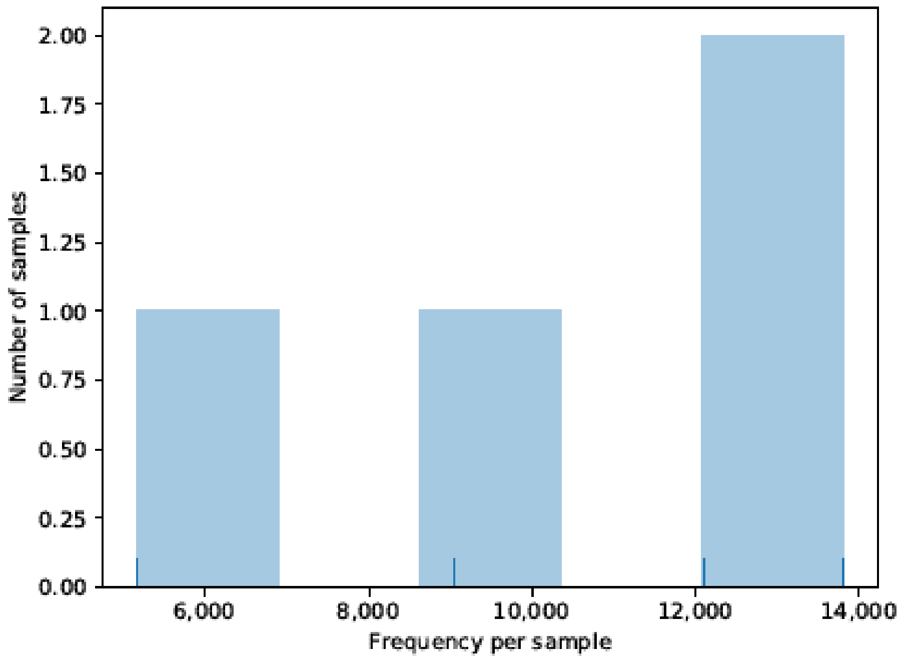

Table S1. BLAST results from B4 sponge (*Mycale* (*Oxymycale*) *acerata*). The primer names, sequence length in base pairs (bp), first hits (highlighted in bold), hits at low significance displaying the correct species (where present), query cover and identity percentages (%) were reported.

Primers	Sequence length (bp)	BLAST results	Query cover (%)	Identity (%)
A/B	1700	***Mycale macilenta* voucher 0CDN7203-O small subunit 18S ribosomal RNA gene, partial sequence**	99	98
18S-AF/18S-BR	1700	***Mycale macilenta* voucher 0CDN7203-O small subunit 18S ribosomal RNA gene, partial sequence**	99	99
RA2/ITS2.2	700	***Mycale grandis* isolate SPO4A small subunit ribosomal RNA gene, partial sequence; internal transcribed spacer 1, 5.8S ribosomal RNA gene, and internal transcribed spacer 2, complete sequence; and large subunit ribosomal RNA gene, partial sequence**	61	81
NL4F/NL4R	1000	***Mycale macilenta* voucher NCI346 28S ribosomal RNA gene, partial sequence**	99	97
PorCOI2fwd/ PorCOI2rev	577	***Asbestopluma lycopodium* mitochondrial partial mRNA for cytochrome oxidase subunit 1 (COI gene), specimen voucher ZMBN103466**	98	90
		Mycale acerata isolate Myc_HH71 cytochrome c oxidase subunit I (COX1) gene, partial cds; mitochondrial	55	99
dgLCO1490/ dgHCO2198	694	***Mycale acerata* cytochrome c oxidase subunit I (COI) gene, partial cds; mitochondrial**	91	99

Table S2. BLAST results from C6 sponge (*Haliclona* (*Rhizoniera*) *dancoi*). The primer names, sequence length in base pairs (bp), first hits (highlighted in bold), hits at low significance displaying the correct species (where present), query cover and identity percentages (%) were reported.

Primers	Sequence lenght (bp)	BLAST results	Query cover (%)	Identity (%)
18S-AF/18S-BR	1867	***Haliclona* sp. DS-2007a 18S ribosomal RNA gene, partial sequence**	97	92
NL4F/NL4R	1144	***Amphimedon queenslandica* large subunit ribosomal RNA gene, partial sequence**	99	90

Table S3. BLAST results from D4 sponge (*Hemigellius pilosus*). The primer names, sequence length in base pairs (bp), first hits (highlighted in bold), hits at low significance displaying the correct species (where present), query cover and identity percentages (%) were reported.

Primers	Sequence lenght (bp)	BLAST results	Query cover (%)	Identity (%)
PorCOI2fwd/ PorCOI2rev	625	***Gelliodes wilsoni* cytochrome c oxidase subunit I (COX1) gene, partial cds; mitochondrial**	99	94
dgLCO1490/ dgHCO2198	572	***Hemigellius pilosus* mitochondrial partial COI gene for cytochrome oxidase subunit 1, specimen voucher NIWA29200**	94	98

Table S4. BLAST results from D6 sponge (*Microxina sarai*). The primer names, sequence length in base pairs (bp), first hits (highlighted in bold), query cover and identity percentages (%) were reported.

Primers	Sequence lenght (bp)	BLAST results	Query cover (%)	Identity (%)
A/B	1500	***Haliclona* sp. DS-2007a 18S ribosomal RNA gene, partial sequence**	98	92
18S1/18S2	1900	***Petrosia strongylata* voucher 0CDN9767-Z small subunit 18S ribosomal RNA gene, partial sequence**	94	90
18S-AF/18S-BR	1300	***Haliclona* sp. DS-2007a 18S ribosomal RNA gene, partial sequence**	95	92
NL4F/NL4R	1100	***Amphimedon queenslandica* large subunit ribosomal RNA gene, partial sequence**	98	90
PorCOI2fwd/ PorCOI2rev	591	***Gelliodes wilsoni* cytochrome c oxidase subunit I (COX1) gene, partial cds; mitochondrial**	96	94
dgLCO1490/ dgHCO2198	623	***Niphatidae* sp. 3 PRT-2020 voucher TS4542 cytochrome c oxidase subunit I (COX1) gene, partial cds; mitochondrial**	99	95

Table S5. ASVs found from *M.* (*Oxymycale*) *acerata* with percentage of confidence ≥ 75%.

Feature ID	Number of sequences	Confidence	Taxon
a4ea6d60a8f9e63298a5ea75868d3c43	1427	0.912665256	D_0__Bacteria;D_1__Proteobacteria;D_2__Gammaproteobacteria;D_3__Betaproteobacteriales;D_4__EC94;D_5__uncultured bacterium;D_6__uncultured bacterium
9f1ed010890d69a37368ea5e92ad5ae2	378	0.999816003	D_0__Bacteria;D_1__Proteobacteria;D_2__Gammaproteobacteria;D_3__Oceanospirillales;D_4__Endozoicomonadaceae;D_5__Endozoicomonas
1b593a6300ad92a0d3c14fa1b1d769be	210	0.998855494	D_0__Bacteria;D_1__Bacteroidetes;D_2__Bacteroidia;D_3__Cytophagales;D_4__Cyclobacteriaceae;D_5__Fulvivirga;D_6__uncultured bacterium
d1296951cd07ad936917eb5a529b8519	171	0.999867479	D_0__Bacteria;D_1__Proteobacteria;D_2__Gammaproteobacteria;D_3__Oceanospirillales;D_4__Endozoicomonadaceae;D_5__Endozoicomonas
62a2b95a760428a5dcc4142aa39cb8c5	136	0.996765346	D_0__Bacteria;D_1__Verrucomicrobia;D_2__Verrucomicrobiae;D_3__Verrucomicrobiales;D_4__Rubritaleaceae;D_5__Rubritalea;D_6__uncultured bacterium
e2c1342958768738f33d30d8ad6dddaf	136	0.999992101	D_0__Bacteria;D_1__Proteobacteria;D_2__Gammaproteobacteria;D_3__Alteromonadales;D_4__Colwelliaceae;D_5__Colwellia
a1020a368dc2ed3acbfe0b52849310cf	103	0.889236536	D_0__Bacteria;D_1__Bacteroidetes;D_2__Bacteroidia;D_3__Flavobacteriales;D_4__Flavobacteriaceae;D_5__Ulvibacter;Ambiguous_taxa
ad7c0f31a1c10c1c69ce29ca1a8fe9bc	68	0.999708203	D_0__Bacteria;D_1__Proteobacteria;D_2__Gammaproteobacteria;D_3__Betaproteobacteriales;D_4__Methylophilaceae;D_5__Methylotenera
f40efeb4eaab598159be6c09c53e1794	66	0.985880058	D_0__Bacteria;D_1__Proteobacteria;D_2__Gammaproteobacteria;D_3__Alteromonadales;D_4__Alteromonadaceae;D_5__Paraglaciecola;Ambiguous_taxa
895f8d4d0116fba8ab4ace8506d42270	61	0.999993017	D_0__Bacteria;D_1__Bacteroidetes;D_2__Bacteroidia;D_3__Flavobacteriales;D_4__Flavobacteriaceae;D_5__Maritimimonas;D_6__uncultured marine bacterium
3c9e154bb7add5d96a146d47a50b0928	51	0.999877938	D_0__Bacteria;D_1__Proteobacteria;D_2__Gammaproteobacteria;D_3__Oceanospirillales;D_4__Endozoicomonadaceae;D_5__Endozoicomonas
96ed4932292a58087478ccba3a67eb52	50	0.999999433	D_0__Bacteria;D_1__Proteobacteria;D_2__Gammaproteobacteria;D_3__Oceanospirillales;D_4__Endozoicomonadaceae;D_5__Endozoicomonas
278fb2198b21befc38f8943ca61e98cc	48	0.95188884	D_0__Bacteria;D_1__Verrucomicrobia;D_2__Verrucomicrobiae;D_3__Verrucomicrobiales;D_4__Rubritaleaceae;D_5__Rubritalea;D_6__uncultured bacterium
ee870ce17e3f15f8901fd69b429fcef4	47	0.999990415	D_0__Bacteria;D_1__Proteobacteria;D_2__Gammaproteobacteria;D_3__Alteromonadales;D_4__Colwelliaceae;D_5__Colwellia

b0622d44882b29138fe6e2d4d2ce5ad1	45	0.99985596	D_0__Bacteria;D_1__Proteobacteria;D_2__Gammaproteobacteria;D_3__Alteromonadales;D_4__Colwelliaceae;D_5__Colwellia
9d65f1d36e791afe1c55e4e5cd43fbe4	43	0.999999982	D_0__Bacteria;D_1__Verrucomicrobia;D_2__Verrucomicrobiae;D_3__Verrucomicrobiales;D_4__Rubritaleaceae;D_5__Rubritalea;D_6__uncultured bacterium
fd1b630abd30a2d66d755191318cad6a	34	0.999955984	D_0__Bacteria;D_1__Proteobacteria;D_2__Alphaproteobacteria;D_3__Kordiimonadales;D_4__uncultured;D_5__uncultured alpha proteobacterium;D_6__uncultured alpha proteobacterium
b9a1e544e1001768214eab4d2456427b	33	0.994559771	D_0__Bacteria;D_1__Proteobacteria;D_2__Gammaproteobacteria;D_3__Oceanospirillales;D_4__Kangiellaceae;D_5__uncultured;Ambiguous_taxa
b3cff3bb35803df4da9e0a622938098f	30	0.941528817	D_0__Bacteria;D_1__Bacteroidetes;D_2__Bacteroidia;D_3__Flavobacteriales;D_4__Flavobacteriaceae;D_5__Ulvibacter;Ambiguous_taxa
b003e0a1d135733e3e047728e3f21bea	29	0.998562648	D_0__Bacteria;D_1__Bacteroidetes;D_2__Bacteroidia;D_3__Cytophagales;D_4__Cyclobacteriaceae;D_5__Fulvivirga;D_6__uncultured bacterium
40b18ed3bbfe833327078205d4f16471	28	0.9445629	D_0__Bacteria;D_1__Actinobacteria;D_2__Acidimicrobiia;D_3__Microtrichales;D_4__Microtrichaceae;D_5__uncultured;D_6__uncultured Acidimicrobiales bacterium
b75210954630df7794c0f6c01f047407	28	0.999674058	D_0__Bacteria;D_1__Proteobacteria;D_2__Gammaproteobacteria;D_3__Oceanospirillales;D_4__Endozoicomonadaceae;D_5__Endozoicomonas
e8550673c2ac62406b48621aff4f52f2	27	1	D_0__Bacteria;D_1__Bacteroidetes;D_2__Bacteroidia;D_3__Flavobacteriales;D_4__Flavobacteriaceae
159593908836f364fb4f3c5e663a5e59	24	0.811485846	D_0__Bacteria;D_1__Proteobacteria;D_2__Alphaproteobacteria;D_3__Rhodobacterales;D_4__Rhodobacteraceae;D_5__Roseobacter clade NAC11-7 lineage
1cec8261e5fc06ca6b16ac7ac9d3664d	24	0.999999808	D_0__Bacteria;D_1__Proteobacteria;D_2__Gammaproteobacteria;D_3__Cellvibrionales;D_4__Spongiibacteraceae;D_5__Sinobacterium;D_6__uncultured bacterium
2b80824766a33f8fd384da14c8117574	24	0.997598841	D_0__Bacteria;D_1__Actinobacteria;D_2__Acidimicrobiia;D_3__Microtrichales;D_4__Microtrichaceae;D_5__Sva0996 marine group;D_6__uncultured organism
8a31bd6ef7a26c35b0206e8e0b852f74	24	0.999931828	D_0__Bacteria;D_1__Bacteroidetes;D_2__Bacteroidia;D_3__Flavobacteriales;D_4__Flavobacteriaceae;D_5__Maritimimonas;D_6__uncultured marine bacterium
bcc6b5465440970c6c3c97757d3868a9	24	0.998767926	D_0__Bacteria;D_1__Proteobacteria;D_2__Gammaproteobacteria;D_3__Oceanospirillales;D_4__Nitrincolaceae;D_5__uncultured;D_6__uncultured bacterium
0aebc7c8b90e3922ae2960d6bc6ba3f8	23	0.968351558	D_0__Bacteria;D_1__Proteobacteria;D_2__Gammaproteobacteria;D_3__Alteromonadales;D_4__Moritellaceae;D_5__Moritella;Ambiguous_taxa
6edc5627c661f42de7cef55aafae77ae	22	0.910915424	D_0__Bacteria;D_1__Bacteroidetes;D_2__Bacteroidia;D_3__Flavobacteriales;D_4__Flavobacteriaceae;D_5__Algibacter;Ambiguous_taxa

28a9b6d8a9e659f3ba901248608300b3	21	0.878100476	D_0__Bacteria;D_1__Verrucomicrobia;D_2__Verrucomicrobiae;D_3__Verrucomicrobiales;D_4__Rubritaleaceae;D_5__Persicirhabdus;Ambiguous_taxa
6a5b30e33023c1d5a18cbed55b2c3998	21	0.964920543	D_0__Bacteria;D_1__Proteobacteria;D_2__Alphaproteobacteria;D_3__Rhodobacterales;D_4__Rhodobacteraceae;D_5__Amylibacter
c16b5119dc858091596759269edccac0	21	0.900557567	D_0__Bacteria;D_1__Verrucomicrobia;D_2__Verrucomicrobiae;D_3__Verrucomicrobiales;D_4__Rubritaleaceae;D_5__Roseibacillus;D_6__uncultured marine bacterium
7b5dea034b895ccfdd11548ca5b5371c	20	0.932325934	D_0__Bacteria;D_1__Proteobacteria;D_2__Alphaproteobacteria;D_3__Rhodobacterales;D_4__Rhodobacteraceae;D_5__Sulfitobacter;Ambiguous_taxa
c3dda5a58756b031c9e08df9b53b7224	20	0.997756169	D_0__Bacteria;D_1__Proteobacteria;D_2__Alphaproteobacteria;D_3__Rhizobiales;D_4__Rhizobiaceae;D_5__Pseudahrensia;D_6__uncultured bacterium
cc642e360e47b363c9bbd221d2a54399	20	0.920611496	D_0__Bacteria;D_1__Bacteroidetes;D_2__Bacteroidia;D_3__Flavobacteriales;D_4__Flavobacteriaceae;D_5__Maribacter;Ambiguous_taxa
ccb67f3a797ef786dc0c42cf3b8bd220	20	0.999963971	D_0__Bacteria;D_1__Proteobacteria;D_2__Gammaproteobacteria;D_3__Alteromonadales;D_4__Colwelliaceae;D_5__Colwellia
d49ab65491384d592ce52ceb40e4482a	20	0.999653457	D_0__Bacteria;D_1__Actinobacteria;D_2__Acidimicrobiia;D_3__Microtrichales;D_4__Microtrichaceae;D_5__Sva0996 marine group
3fd90259319c61629859866ddf0d2905	19	0.83117912	D_0__Bacteria;D_1__Verrucomicrobia;D_2__Verrucomicrobiae;D_3__Verrucomicrobiales;D_4__Rubritaleaceae;D_5__Rubritalea;D_6__uncultured bacterium
ad9166d7dd6d9dfb179101bd56bdc59e	19	0.745946007	D_0__Bacteria;D_1__Proteobacteria;D_2__Alphaproteobacteria;D_3__Rhodobacterales;D_4__Rhodobacteraceae;D_5__Loktanella;D_6__uncultured bacterium
b4d48f6625d60557067aa2d5f1c3e4ed	19	0.81326208	D_0__Bacteria;D_1__Bacteroidetes;D_2__Bacteroidia;D_3__Flavobacteriales;D_4__Flavobacteriaceae;D_5__Dokdonia;Ambiguous_taxa
faa53b1ce37beb0ae77266df153c2028	19	0.968333622	D_0__Bacteria;D_1__Proteobacteria;D_2__Gammaproteobacteria;D_3__Cellvibrionales;D_4__Spongiibacteraceae;D_5__uncultured;D_6__uncultured bacterium
7d7689c3b0b3830f32c36ab568c7fee3	18	0.999998014	D_0__Bacteria;D_1__Proteobacteria;D_2__Gammaproteobacteria;D_3__Oceanospirillales;D_4__Endozoicomonadaceae;D_5__Endozoicomonas
aecb5b60b758d3e80787bd3a707e3bbd	18	0.752265014	D_0__Bacteria;D_1__Proteobacteria;D_2__Alphaproteobacteria;D_3__Rhodobacterales;D_4__Rhodobacteraceae;D_5__Roseobacter;Ambiguous_taxa
047b36c4f0e57bd73d8377e4a9941a15	17	0.999857817	D_0__Bacteria;D_1__Proteobacteria;D_2__Gammaproteobacteria;D_3__Alteromonadales;D_4__Colwelliaceae;D_5__Colwellia
7f45393e29fb7e75e281f15439a19095	17	0.81311773	D_0__Bacteria;D_1__Proteobacteria;D_2__Gammaproteobacteria;D_3__Cellvibrionales;D_4__Halieaceae;D_5__Halioglobus

d08bc4fcfc17cc6b2be189153c9064d5	17	0.985606948	D_0__Bacteria;D_1__Proteobacteria;D_2__Gammaproteobacteria;D_3__BD7-8
e3e9faf4f891661bb18d96277f1c0e9e	17	1	D_0__Bacteria;D_1__Bacteroidetes;D_2__Bacteroidia;D_3__Flavobacteriales;D_4__Flavobacteriaceae
e420266aac740dddc12375f0765dcc64	17	0.997255745	D_0__Bacteria;D_1__Verrucomicrobia;D_2__Verrucomicrobiae;D_3__Verrucomicrobiales;D_4__Rubritaleaceae;D_5__Rubritalea;D_6__uncultured bacterium
0125be2e39c35fced6c36d7f5e82cdf7	16	0.977061758	D_0__Bacteria;D_1__Proteobacteria;D_2__Gammaproteobacteria;D_3__Oceanospirillales;D_4__Kangiellaceae;D_5__uncultured;Ambiguous_taxa
4a36b1a09856f0a523ca7af7515ba242	16	0.923354141	D_0__Bacteria;D_1__Proteobacteria;D_2__Gammaproteobacteria;D_3__Cellvibrionales;D_4__Microbulbiferaceae;D_5__Microbulbifer;Ambiguous_taxa
590d05f47d955cd512cd0eb539458e3f	16	1	D_0__Bacteria;D_1__Bacteroidetes;D_2__Bacteroidia;D_3__Chitinophagales;D_4__Saprospiraceae;D_5__Lewinella
5f831485aa9ffe3e39cc763379204883	16	0.999950333	D_0__Bacteria;D_1__Bacteroidetes;D_2__Bacteroidia;D_3__Flavobacteriales;D_4__Flavobacteriaceae;D_5__Maritimimonas;D_6__uncultured marine bacterium
84ab1e0355d6a4bbe207b17e384c9248	16	0.999999985	D_0__Bacteria;D_1__Planctomycetes;D_2__Planctomycetacia;D_3__Pirellulales;D_4__Pirellulaceae;D_5__Blastopirellula
90ad0789fa1f71092d9987d92fc8b173	16	0.99999795	D_0__Bacteria;D_1__Spirochaetes;D_2__Spirochaetia;D_3__Spirochaetales;D_4__Spirochaetaceae;D_5__Spirochaeta 2;D_6__uncultured marine bacterium
16cf0f7f0c048095d5c69fabf0868c8a	15	0.98485092	D_0__Bacteria;D_1__Proteobacteria;D_2__Alphaproteobacteria;D_3__Rhodobacterales;D_4__Rhodobacteraceae;D_5__Sulfitobacter;D_6__uncultured marine bacterium
4724bbbe36903e114d17ef5229ae49ba	15	0.92918385	D_0__Bacteria;D_1__Bacteroidetes;D_2__Bacteroidia;D_3__Flavobacteriales;D_4__Flavobacteriaceae;D_5__Winogradskyella;Ambiguous_taxa
7d8305421802790dbeab049b9d75cae2	15	0.766163888	D_0__Bacteria;D_1__Proteobacteria;D_2__Gammaproteobacteria;D_3__Gammaproteobacteria Incertae Sedis;D_4__Unknown Family;D_5__Marinicella;D_6__uncultured bacterium
bdb4d422f0622606ea95a4dd1b17b1d7	15	0.920158259	D_0__Bacteria;D_1__Proteobacteria;D_2__Gammaproteobacteria;D_3__Alteromonadales;D_4__Alteromonadaceae;D_5__Paraglaciecola;Ambiguous_taxa
d0265845e2a2b207cacb4fe7866e7dcd	15	0.856298732	D_0__Bacteria;D_1__Proteobacteria;D_2__Gammaproteobacteria;D_3__Oceanospirillales;D_4__Pseudohongiellaceae;D_5__Pseudohongiella;D_6__uncultured marine bacterium
ce42b14845e10ad958ca1cb53045dbca	14	0.995966837	D_0__Bacteria;D_1__Proteobacteria;D_2__Gammaproteobacteria;D_3__Alteromonadales;D_4__Colwelliaceae;D_5__Colwellia
2062b1a374f102a30880e12b90cc1e89	13	1	D_0__Bacteria;D_1__Proteobacteria;D_2__Alphaproteobacteria;D_3__Rhodobacterales;D_4__Rhodobacteraceae

4edb032cf8d7c83d2902426e792749d5	13	0.983474655	D_0__Bacteria;D_1__Proteobacteria;D_2__Gammaproteobacteria;D_3__Nitrosococcales;D_4__Methylophagaceae;D_5__uncultured;Ambiguous_taxa
52b4f46250c3263f256229b5954b7201	13	0.93433736	D_0__Bacteria;D_1__Bacteroidetes;D_2__Bacteroidia;D_3__Flavobacteriales;D_4__Cryomorphaceae;D_5__uncultured;Ambiguous_taxa
5b89b8b9b143bd19e2f738356fd40d87	13	0.978871778	D_0__Bacteria;D_1__Proteobacteria;D_2__Gammaproteobacteria;D_3__Thiotrichales;D_4__Thiotrichaceae;D_5__Leucothrix;Ambiguous_taxa
8101484a5c241eb9197f3f5ed275e28d	13	0.999999995	D_0__Bacteria;D_1__Proteobacteria;D_2__Alphaproteobacteria;D_3__Rhodobacterales;D_4__Rhodobacteraceae
a790226f2d39a6b1eacb449e2b6fbe26	13	0.993397935	D_0__Bacteria;D_1__Proteobacteria;D_2__Gammaproteobacteria;D_3__Oceanospirillales;D_4__Kangiellaceae;D_5__uncultured;Ambiguous_taxa
ce64d54ce8204b1e5a227ad9d260bfe2	13	0.999975375	D_0__Bacteria;D_1__Proteobacteria;D_2__Gammaproteobacteria;D_3__Betaproteobacteriales;D_4__Methylophilaceae;D_5__Methylotenera
d5f424da453819b39fe6c6bf2b901997	13	0.984993474	D_0__Bacteria;D_1__Proteobacteria;D_2__Gammaproteobacteria;D_3__Oceanospirillales;D_4__Kangiellaceae;D_5__uncultured;Ambiguous_taxa
ee0f5caff94365896ef095e26d1fed6d	13	0.749932806	D_0__Bacteria;D_1__Proteobacteria;D_2__Alphaproteobacteria;D_3__SAR11 clade;D_4__Clade I;D_5__Clade Ia
4222f44306fe312dc21c42d676f9ce65	12	0.986315118	D_0__Bacteria;D_1__Verrucomicrobia;D_2__Verrucomicrobiae;D_3__Verrucomicrobiales;D_4__Rubritaleaceae;D_5__Persicirhabdus;D_6__uncultured bacterium
5d9835c028d02be320fdd0c95eb23f4f	12	0.82943598	D_0__Bacteria;D_1__Bacteroidetes;D_2__Bacteroidia;D_3__Flavobacteriales;D_4__Flavobacteriaceae;D_5__Polaribacter 1;Ambiguous_taxa
646a76fd0777100707a2af1a3344be46	12	0.998330898	D_0__Bacteria;D_1__Proteobacteria;D_2__Alphaproteobacteria;D_3__Rhodospirillales;D_4__Magnetospiraceae;D_5__Magnetospira;D_6__uncultured marine bacterium
8248b5ca3cabf57e63291c0250b18cd8	12	0.928013931	D_0__Bacteria;D_1__Proteobacteria;D_2__Gammaproteobacteria;D_3__Alteromonadales;D_4__Shewanellaceae;D_5__Psychrobium;D_6__uncultured bacterium
b4abd5eea6527558dc4cc070b6b70032	12	0.891154075	D_0__Bacteria;D_1__Actinobacteria;D_2__Acidimicrobiia;D_3__Microtrichales;D_4__Ilumatobacteraceae;D_5__Ilumatobacter;Ambiguous_taxa
d99a727d18d025d687533115516c7cbb	12	0.999999999	D_0__Bacteria
fac52f1f824f78b6cf50a3268831ae82	12	0.879684082	D_0__Bacteria;D_1__Actinobacteria;D_2__Acidimicrobiia;D_3__Microtrichales;D_4__Ilumatobacteraceae;D_5__Ilumatobacter;Ambiguous_taxa
0243483399bf33f0de6658ac1b2258c8	11	0.782258864	D_0__Bacteria;D_1__Proteobacteria;D_2__Alphaproteobacteria;D_3__Rhodobacterales;D_4__Rhodobacteraceae;D_5__Loktanella

46c5e74f2284235ed905dc88eb991569	11	0.988708557	D_0__Bacteria;D_1__Proteobacteria;D_2__Gammaproteobacteria;D_3__Oceanospirillales;D_4__Kangiellaceae;D_5__uncultured;Ambiguous_taxa
535b9ebd7316c464b0b200ab675833db	11	0.86318965	D_0__Bacteria;D_1__Proteobacteria;D_2__Alphaproteobacteria;D_3__Rhodobacterales;D_4__Rhodobacteraceae;D_5__Amylibacter
a007abe4d82829eb7626d11282a92935	11	0.999999988	D_0__Bacteria;D_1__Planctomycetes;D_2__Planctomycetacia;D_3__Pirellulales;D_4__Pirellulaceae;D_5__Blastopirellula
b23c69c6735213b55453abe9642b1af1	11	0.999386499	D_0__Bacteria;D_1__Bacteroidetes;D_2__Bacteroidia;D_3__Chitinophagales;D_4__Saprospiraceae;D_5__Lewinella;D_6__uncultured marine bacterium
cd9102d8a7aa4d9602a128a83ac80491	11	0.960424736	D_0__Bacteria;D_1__Bacteroidetes;D_2__Bacteroidia;D_3__Flavobacteriales;D_4__Flavobacteriaceae;D_5__Winogradskyella;Ambiguous_taxa
d966d20d69c1c82424702adf6e07f488	11	0.999638568	D_0__Bacteria;D_1__Bacteroidetes;D_2__Bacteroidia;D_3__Cytophagales;D_4__Cyclobacteriaceae;D_5__uncultured;D_6__uncultured bacterium
e9ca6bfaab0bfb3275a138393fb49674	11	0.885402338	D_0__Bacteria;D_1__Proteobacteria;D_2__Gammaproteobacteria;D_3__Arenicellales;D_4__Arenicellaceae;D_5__Arenicella;Ambiguous_taxa
f0e6eac9bd44f060d5f5ccc49cdbc395	11	0.96119322	D_0__Bacteria;D_1__Proteobacteria;D_2__Gammaproteobacteria;D_3__Thiohalorhabdales;D_4__Thiohalorhabdaceae;D_5__Granulosicoccus;D_6__uncultured marine bacterium
fda1d23e0f057b10e04e90852eb64121	11	0.981348293	D_0__Bacteria;D_1__Proteobacteria;D_2__Alphaproteobacteria;D_3__Sneathiellales;D_4__Sneathiellaceae;D_5__Sneathiella;D_6__uncultured bacterium
0696faca32374e98e0bc77e4d5bd1bc2	10	0.999999971	D_0__Bacteria;D_1__Proteobacteria;D_2__Alphaproteobacteria;D_3__Rhodobacterales;D_4__Rhodobacteraceae
3161bf8d4e50e0cc41ed28dfadb75ee2	10	0.892241844	D_0__Bacteria;D_1__Proteobacteria;D_2__Alphaproteobacteria;D_3__Rhizobiales;D_4__Rhizobiaceae;D_5__uncultured;Ambiguous_taxa
45d75404c5a89925e20d9a4d893e59f1	10	0.992257377	D_0__Bacteria;D_1__Proteobacteria;D_2__Gammaproteobacteria;D_3__Gammaproteobacteria Incertae Sedis;D_4__Unknown Family;D_5__Marinicella;Ambiguous_taxa
4abb22cdd91561294fd1e8fd67604648	10	0.763183871	D_0__Bacteria;D_1__Bacteroidetes;D_2__Bacteroidia;D_3__Flavobacteriales;D_4__Flavobacteriaceae;D_5__[Polaribacter] huanghezhanensis;Ambiguous_taxa
6bbbd6e7960913833d03b6c0258bc7fb	10	0.999997674	D_0__Bacteria;D_1__Proteobacteria;D_2__Gammaproteobacteria
9ae4b8e315c734f70e112ac5da49f2d2	10	0.967388972	D_0__Bacteria;D_1__Proteobacteria;D_2__Gammaproteobacteria;D_3__Arenicellales;D_4__Arenicellaceae;D_5__Arenicella;D_6__uncultured gamma proteobacterium
d2c7ce967205a06fd70e3e3ec8972711	10	0.908992998	D_0__Bacteria;D_1__Proteobacteria;D_2__Gammaproteobacteria;D_3__Thiotrichales;D_4__Thiotrichaceae;D_5__Thiothrix;Ambiguous_taxa

d86f57d65d0302308bb4f571a88ba49c	10	0.999999997	D_0__Bacteria;D_1__Proteobacteria;D_2__Deltaproteobacteria;D_3__NB1-j
de9232f2da52576b10923892d9c5e458	10	0.969250635	D_0__Bacteria;D_1__Planctomycetes;D_2__Pla4 lineage;D_3__uncultured bacterium;D_4__uncultured bacterium;D_5__uncultured bacterium;D_6__uncultured bacterium
ea7e0dea2b1f4768ca6e0b237286f9ef	10	0.986328456	D_0__Bacteria;D_1__Proteobacteria;D_2__Alphaproteobacteria;D_3__Rhizobiales;D_4__Hyphomicrobiaceae;D_5__Filomicrobium;Ambiguous_taxa
ece89bff980b08b04d6f8dbe287592c3	10	0.999999069	D_0__Bacteria;D_1__Epsilonbacteraeota;D_2__Campylobacteria;D_3__Campylobacterales;D_4__Arcobacteraceae;D_5__Arcobacter
f7119682fee25ac95e6ba65510ff35c3	10	0.999999975	D_0__Bacteria;D_1__Proteobacteria;D_2__Alphaproteobacteria;D_3__Sphingomonadales;D_4__Sphingomonadaceae
fe6b598825e6dece979bdbd18a10010f	10	0.809945138	D_0__Bacteria;D_1__Bacteroidetes;D_2__Bacteroidia;D_3__Flavobacteriales;D_4__Flavobacteriaceae;D_5__Zobellia;Ambiguous_taxa
1810c361b5f532d3e1fcc13936d9de2f	9	0.999997998	D_0__Bacteria;D_1__Bacteroidetes;D_2__Bacteroidia;D_3__Cytophagales;D_4__Cyclobacteriaceae;D_5__Reichenbachiella
5e68f782c00efaf729c4ccd474450acf	9	0.979549727	D_0__Bacteria;D_1__Proteobacteria;D_2__Gammaproteobacteria;D_3__OM182 clade;D_4__uncultured bacterium;D_5__uncultured bacterium;D_6__uncultured bacterium
75e665d14cc29ed1ba519d7ee7016480	9	0.999999493	D_0__Bacteria;D_1__Bacteroidetes;D_2__Bacteroidia;D_3__Flavobacteriales;D_4__Cryomorphaceae;D_5__Cryomorpha;D_6__uncultured deep-sea bacterium
9e4bb52a30262dd18e9cc6ee7a51b08f	9	0.943042595	D_0__Bacteria;D_1__Proteobacteria;D_2__Alphaproteobacteria;D_3__Micavibrionales;D_4__Micavibrionaceae;D_5__uncultured;D_6__uncultured bacterium
a5577541cbf99c61a853c834987ac17d	9	1	D_0__Bacteria;D_1__Proteobacteria;D_2__Gammaproteobacteria;D_3__Gammaproteobacteria Incertae Sedis;D_4__Unknown Family;D_5__Marinicella
c5b9ab8efdd93dbb18591809e7482ac4	9	0.999994524	D_0__Bacteria;D_1__Proteobacteria;D_2__Gammaproteobacteria;D_3__Thiotrichales;D_4__Thiotrichaceae;D_5__Cocleimonas
ed347145ab935ef912e1f90fe9590298	9	0.886294614	D_0__Bacteria;D_1__Proteobacteria;D_2__Gammaproteobacteria;D_3__Alteromonadales;D_4__Colwelliaceae;D_5__Colwellia
fa563495ba0f5215a6a8403ec332132d	9	0.890569128	D_0__Bacteria;D_1__Proteobacteria;D_2__Alphaproteobacteria;D_3__Rhodobacterales;D_4__Rhodobacteraceae;D_5__Loktanella
04b637f3c78c015d30b30f5b5168eddd	8	0.992069762	D_0__Bacteria;D_1__Verrucomicrobia;D_2__Verrucomicrobiae;D_3__Verrucomicrobiales;D_4__DEV007;D_5__uncultured Verrucomicrobia bacterium;D_6__uncultured Verrucomicrobia bacterium
0d42c6bc61177509c486c8ca0ca148c3	8	0.768296898	D_0__Bacteria;D_1__Bacteroidetes;D_2__Bacteroidia;D_3__Flavobacteriales;D_4__Schleiferiaceae;D_5__Schleiferia;D_6__uncultured Bacteroidetes bacterium

1f886dfa8f6136eab4660b909667dea0	8	0.945078418	D_0__Bacteria;D_1__Bacteroidetes;D_2__Bacteroidia;D_3__Cytophagales;D_4__Cyclobacteriaceae;D_5__Fabibacter;Ambiguous_taxa
2b27a33e6824822674ccf9d01b914d57	8	0.999611345	D_0__Bacteria;D_1__Bacteroidetes;D_2__Bacteroidia;D_3__Chitinophagales;D_4__Saprospiraceae;D_5__Rubidimonas;D_6__uncultured bacterium
338ee77aa8b156ae36c5afb123bb1753	8	0.956607869	D_0__Bacteria;D_1__Proteobacteria;D_2__Gammaproteobacteria;D_3__Arenicellales;D_4__Arenicellaceae;D_5__Arenicella;D_6__uncultured bacterium
51a5de4227edafec4841d560fb4ea06b	8	0.999998197	D_0__Bacteria;D_1__Proteobacteria;D_2__Gammaproteobacteria;D_3__Oceanospirillales;D_4__Pseudohongiellaceae;D_5__Pseudohongiella
537a2abd146168ac7581149542b319e6	8	0.806271184	D_0__Bacteria;D_1__Bacteroidetes;D_2__Bacteroidia;D_3__Flavobacteriales;D_4__Flavobacteriaceae;D_5__Maribacter
64baf572d00835f3cb89cbae732974a0	8	0.999972029	D_0__Bacteria;D_1__Bacteroidetes;D_2__Bacteroidia;D_3__Flavobacteriales;D_4__Flavobacteriaceae;D_5__Aquibacter
70ec781c936311855ea407954453c92d	8	0.998960065	D_0__Bacteria;D_1__Bacteroidetes;D_2__Bacteroidia;D_3__Flavobacteriales;D_4__Flavobacteriaceae;D_5__Winogradskyella
7c48952a1bb1a9df5f303579e6f7771f	8	0.982926121	D_0__Bacteria;D_1__Verrucomicrobia;D_2__Verrucomicrobiae;D_3__Verrucomicrobiales;D_4__Rubritaleaceae;D_5__Persicirhabdus;D_6__uncultured bacterium
af9c9edf8abdefd03492b10b0bd864bb	8	0.981213681	D_0__Bacteria;D_1__Proteobacteria;D_2__Gammaproteobacteria;D_3__Alteromonadales;D_4__Alteromonadaceae;D_5__Paraglaciecola;Ambiguous_taxa
b99d6738a0771a81b47cab4575e57b2b	8	0.991838	D_0__Bacteria;D_1__Bacteroidetes;D_2__Bacteroidia;D_3__Flavobacteriales;D_4__Schleiferiaceae;D_5__Schleiferia;Ambiguous_taxa
baf6cf911eb4d01ae16f3451a6640883	8	0.999978894	D_0__Bacteria;D_1__Bacteroidetes;D_2__Bacteroidia;D_3__Flavobacteriales;D_4__Flavobacteriaceae
2ed48e3b0a583bc636767e0dc2609274	7	0.994292422	D_0__Bacteria;D_1__Bacteroidetes;D_2__Bacteroidia;D_3__Flavobacteriales;D_4__Flavobacteriaceae;D_5__Ulvibacter;D_6__uncultured marine bacterium
7874f80382e597a6eb3bf95a6defd394	7	0.875967844	D_0__Bacteria;D_1__Fusobacteria;D_2__Fusobacteriia;D_3__Fusobacteriales;D_4__Fusobacteriaceae;D_5__Psychrilyobacter;D_6__uncultured bacterium
8852ae6351d0132687162bacbf5c558e	7	0.945950996	D_0__Bacteria;D_1__Bacteroidetes;D_2__Bacteroidia;D_3__Flavobacteriales;D_4__Flavobacteriaceae;D_5__Arcticiflavibacter;D_6__uncultured bacterium
9390711f3044e7aba9cb577a0aaaf34e	7	0.999999983	D_0__Bacteria;D_1__Bacteroidetes;D_2__Bacteroidia;D_3__Flavobacteriales;D_4__Flavobacteriaceae;D_5__Pseudofulvibacter
b0dd91d8d7c89d5dfcf4c312564821e8	7	0.999999919	D_0__Bacteria;D_1__Proteobacteria;D_2__Gammaproteobacteria;D_3__Tenderiales;D_4__Tenderiaceae;D_5__Candidatus Tenderia

d753a45b28b4542443dbfd12cba83a5b	7	0.999854193	D_0__Bacteria;D_1__Bacteroidetes;D_2__Bacteroidia;D_3__Chitinophagales;D_4__uncultured;D_5__uncultured Sphingobacteriales bacterium;D_6__uncultured Sphingobacteriales bacterium
d82a1758e5d6ed55452b2fa01dc32240	7	0.920591936	D_0__Bacteria;D_1__Proteobacteria;D_2__Gammaproteobacteria;D_3__Thiotrichales;D_4__Thiotrichaceae;D_5__Leucothrix;Ambiguous_taxa
df3485238d28be83c8532a8a909c8f59	7	0.999991163	D_0__Bacteria;D_1__Bacteroidetes;D_2__Bacteroidia;D_3__Cytophagales;D_4__Cyclobacteriaceae;D_5__Reichenbachiella;D_6__uncultured bacterium
fae896f1fb945462f67f3a67a0d79954	7	0.877468478	D_0__Bacteria;D_1__Bacteroidetes;D_2__Bacteroidia;D_3__Flavobacteriales;D_4__Flavobacteriaceae;D_5__uncultured
fd556660b39a8d9d68f05271539b9ef6	7	0.91165048	D_0__Bacteria;D_1__Proteobacteria;D_2__Gammaproteobacteria;D_3__Oceanospirillales;D_4__Oleiphilaceae;D_5__Oleiphilus;D_6__uncultured gamma proteobacterium
0734ba045054b53eb03f53a7e74ce39d	6	0.966462036	D_0__Bacteria;D_1__Proteobacteria;D_2__Deltaproteobacteria;D_3__Myxococcales;D_4__Blfdi19;D_5__uncultured bacterium;D_6__uncultured bacterium
07694b01b7ea9e9a39899530f7541af9	6	0.97574035	D_0__Bacteria;D_1__Bacteroidetes;D_2__Bacteroidia;D_3__Flavobacteriales;D_4__Flavobacteriaceae;D_5__Ulvibacter
0b04405baedf51a425f743c1d9e6905a	6	0.760940266	D_0__Bacteria;D_1__Proteobacteria;D_2__Alphaproteobacteria;D_3__Caulobacterales;D_4__Hyphomonadaceae;D_5__uncultured;D_6__uncultured alpha proteobacterium
114ff11a2b5085191d3af94b3db86ce7	6	0.991156807	D_0__Bacteria;D_1__Proteobacteria;D_2__Alphaproteobacteria;D_3__Rhizobiales;D_4__Rhizobiaceae
43701eb4ff6823885b53f82c7aa3b41e	6	0.999965685	D_0__Bacteria;D_1__Proteobacteria;D_2__Gammaproteobacteria;D_3__Oceanospirillales;D_4__Pseudohongiellaceae;D_5__Pseudohongiella
440876b3d8ac4365b1f63f93be24f02d	6	0.900456527	D_0__Bacteria;D_1__Bacteroidetes;D_2__Bacteroidia;D_3__Flavobacteriales;D_4__Crocinitomicaceae;D_5__Crocinitomix;Ambiguous_taxa
8249a563d2c03ee2f719dc5d24fb0c00	6	1	D_0__Bacteria;D_1__Bacteroidetes;D_2__Bacteroidia;D_3__Flavobacteriales;D_4__Flavobacteriaceae
83c82b926eb576ecbf92daf178a21267	6	0.819204897	D_0__Bacteria;D_1__Proteobacteria;D_2__Gammaproteobacteria;D_3__Steroidobacterales;D_4__Woeseiaceae;D_5__Woeseia
b69b0d3890492c3a332d9eb69a0b28b3	6	0.898447454	D_0__Bacteria;D_1__Bacteroidetes;D_2__Bacteroidia;D_3__Flavobacteriales;D_4__Flavobacteriaceae;D_5__Pseudofulvibacter;Ambiguous_taxa
badc7e0169c2aa5ef4e044cbb2b6c500	6	0.959497183	D_0__Bacteria;D_1__Bacteroidetes;D_2__Bacteroidia;D_3__Flavobacteriales;D_4__Flavobacteriaceae;D_5__Lutimonas;Ambiguous_taxa
bd7f3a03456c7658ca48b649af8eeb56	6	0.988450034	D_0__Bacteria;D_1__Proteobacteria;D_2__Gammaproteobacteria;D_3__OM182 clade;Ambiguous_taxa;Ambiguous_taxa;Ambiguous_taxa

d58d9619c3feb93a407d1b654342c852	6	0.996470124	D_0__Bacteria;D_1__Bacteroidetes;D_2__Bacteroidia;D_3__Flavobacteriales;D_4__Flavobacteriaceae;D_5__Kordia;Ambiguous_taxa
d5d849c8ae6cbf5fa1d626575c365468	6	0.897136029	D_0__Bacteria;D_1__Proteobacteria;D_2__Gammaproteobacteria;D_3__Alteromonadales;D_4__Alteromonadaceae;D_5__Paraglaciecola;Ambiguous_taxa
ea5e40c523be5744ce50bc9c24aae93c	6	0.970685711	D_0__Bacteria;D_1__Proteobacteria;D_2__Alphaproteobacteria
fe63eab2baa2a22eb52cb32d332511f6	6	0.994538372	D_0__Bacteria;D_1__Verrucomicrobia;D_2__Verrucomicrobiae;D_3__Verrucomicrobiales;D_4__DEV007;D_5__uncultured Verrucomicrobia bacterium;D_6__uncultured Verrucomicrobia bacterium
fe6cb0afe45ab56fde5f6491e7a4853f	6	0.999995781	D_0__Bacteria;D_1__Proteobacteria;D_2__Gammaproteobacteria;D_3__Cellvibrionales;D_4__Halieaceae
00a8468015f87dce01d98ed62581d8f9	5	0.928700511	D_0__Bacteria;D_1__Bacteroidetes;D_2__Bacteroidia;D_3__Chitinophagales;D_4__uncultured;D_5__uncultured Bacteroidetes bacterium;D_6__uncultured Bacteroidetes bacterium
083a82373b22ec10045bdabce3b6c45a	5	0.999999952	D_0__Bacteria;D_1__Proteobacteria;D_2__Alphaproteobacteria;D_3__Rhodovibrionales;D_4__Kiloniellaceae;D_5__Kiloniella
1ea66ec48c7d65a3a472aa4245af1c90	5	0.850724554	D_0__Bacteria;D_1__Bacteroidetes;D_2__Bacteroidia;D_3__Chitinophagales;D_4__Saprospiraceae;D_5__uncultured;D_6__uncultured Bacteroidetes bacterium
24510bb1d95a4c4eafd3a1022b214d05	5	0.812095923	D_0__Bacteria;D_1__Bacteroidetes;D_2__Bacteroidia;D_3__Flavobacteriales;D_4__Flavobacteriaceae;D_5__Polaribacter
249174fb86128c197098f81aba111996	5	0.95352615	D_0__Bacteria;D_1__Proteobacteria;D_2__Gammaproteobacteria;D_3__Arenicellales;D_4__Arenicellaceae;D_5__Arenicella;D_6__uncultured gamma proteobacterium
2aa7103bf5226d561f5315d393a0527d	5	0.926619565	D_0__Bacteria;D_1__Proteobacteria;D_2__Alphaproteobacteria;D_3__Micavibrionales;D_4__Micavibrionaceae;D_5__uncultured;D_6__uncultured bacterium
2bb4d67a7d8c3e4c647ed17fd072e74d	5	0.867904373	D_0__Bacteria;D_1__Verrucomicrobia;D_2__Verrucomicrobiae;D_3__Verrucomicrobiales;D_4__DEV007;D_5__uncultured deep-sea bacterium;D_6__uncultured deep-sea bacterium
46f1273e4585071df64ff30412ff30ff	5	0.999999914	D_0__Bacteria;D_1__Planctomycetes;D_2__OM190
4aa6874360158baeebfd783061e7ae02	5	0.748410862	D_0__Bacteria;D_1__Proteobacteria;D_2__Gammaproteobacteria;D_3__Cellvibrionales;D_4__Spongiibacteraceae;D_5__BD1-7 clade
6654b008689f523f8b471881dceb2a4c	5	0.999999994	D_0__Bacteria;D_1__Patescibacteria;D_2__Parcubacteria;D_3__Candidatus Campbellbacteria
6cb1587876ef02be596da19701676dab	5	0.946308243	D_0__Bacteria;D_1__Proteobacteria;D_2__Gammaproteobacteria;D_3__Cellvibrionales;D_4__Halieaceae;D_5__OM60(NOR5) clade;D_6__uncultured bacterium

6dd2160e7d29115148c388d0ab6cf631	5	0.95469187	D_0__Bacteria;D_1__Proteobacteria;D_2__Gammaproteobacteria;D_3__Cellvibrionales;D_4__Halieaceae;D_5__Halioglobus;D_6__Saccharina japonica
7086bdb7d00fa7ede3793bfaecdda290	5	0.952142225	D_0__Bacteria;D_1__Bacteroidetes;D_2__Bacteroidia;D_3__Chitinophagales;D_4__Saprospiraceae;D_5__Lewinella;D_6__Lewinella agarilytica
71dac9bd043cd476f40ef2d5e7bb28fa	5	0.999999551	D_0__Bacteria;D_1__Proteobacteria;D_2__Gammaproteobacteria;D_3__Alteromonadales;D_4__Psychromonadaceae;D_5__Psychromonas
7b4056c1dc66d0f4b83867fb71337b7b	5	0.998931083	D_0__Bacteria;D_1__Bacteroidetes;D_2__Bacteroidia;D_3__Bacteroidetes VC2.1 Bac22
8d3f3ea41db5c368cb519d56ea863612	5	0.967409506	D_0__Bacteria;D_1__Proteobacteria;D_2__Alphaproteobacteria;D_3__Rickettsiales;D_4__Rickettsiaceae;D_5__Candidatus Megaira;Ambiguous_taxa
8ffa9c2df90158e2e2cfa6c4ccaa0b52	5	0.99906136	D_0__Bacteria;D_1__Proteobacteria;D_2__Gammaproteobacteria;D_3__KI89A clade;D_4__uncultured gamma proteobacterium;D_5__uncultured gamma proteobacterium;D_6__uncultured gamma [illegible]
908a80f39bd18379e9e6f2ab9948e477	5	0.764072575	D_0__Bacteria;D_1__Bacteroidetes;D_2__Bacteroidia;D_3__Chitinophagales;D_4__Saprospiraceae;D_5__uncultured;D_6__uncultured Bacteroidetes bacterium
c1ea9b3d65046fc3117ed5ee17c4b8a1	5	0.990127023	D_0__Bacteria;D_1__Bacteroidetes;D_2__Bacteroidia;D_3__Flavobacteriales;D_4__Cryomorphaceae;D_5__uncultured
c50c14389abdb643c592052582edc4b4	5	0.782694916	D_0__Bacteria;D_1__Proteobacteria;D_2__Gammaproteobacteria;D_3__Gammaproteobacteria Incertae Sedis;D_4__Unknown Family;D_5__Marinicella;Ambiguous_taxa
c84d321d0e04bd8202dd6e1ae1ac41b1	5	0.999998793	D_0__Bacteria;D_1__Proteobacteria;D_2__Gammaproteobacteria;D_3__Arenicellales;D_4__Arenicellaceae;D_5__Arenicella
cd4bbda080d4aeb2afdfbb6d83cb5e8e	5	0.997585394	D_0__Bacteria;D_1__Bacteroidetes;D_2__Bacteroidia;D_3__Chitinophagales;D_4__Saprospiraceae;D_5__uncultured
dc99114ad680ef9f30110cfb2baadd51	5	0.941420646	D_0__Bacteria;D_1__Proteobacteria;D_2__Gammaproteobacteria;D_3__Cellvibrionales;D_4__Halieaceae;D_5__OM60(NOR5) clade
e9c00c498f88216d24379be57c5e2660	5	0.976968192	D_0__Bacteria;D_1__Chloroflexi;D_2__Anaerolineae;D_3__Ardenticatenales;D_4__uncultured;Ambiguous_taxa;Ambiguous_taxa
f011094a245568cfea52fbd6a132cbf7	5	0.999938748	D_0__Bacteria;D_1__Bacteroidetes;D_2__Bacteroidia;D_3__Chitinophagales;D_4__Saprospiraceae;D_5__uncultured;D_6__uncultured marine bacterium
f48d534b86d383e50b59a2c3815b115e	5	0.98347029	D_0__Bacteria;D_1__Bacteroidetes;D_2__Bacteroidia;D_3__Chitinophagales;D_4__Saprospiraceae;D_5__uncultured;D_6__uncultured bacterium
085f909fd9a9edf1a0bbd3798cf5279b	4	0.887600266	D_0__Bacteria;D_1__Proteobacteria;D_2__Gammaproteobacteria;D_3__Thiomicrospirales;D_4__Thioglobaceae;D_5__SUP05 cluster;D_6__uncultured marine bacterium

16a17b6bff10137f8e1a022d6202656a	4	0.955923338	D_0__Bacteria;D_1__Bacteroidetes;D_2__Bacteroidia;D_3__Chitinophagales;D_4__Saprospiraceae;D_5__uncultured;D_6__uncultured bacterium
1842b42087ff314a68c6ec0b87da0753	4	0.936289163	D_0__Bacteria;D_1__Proteobacteria;D_2__Deltaproteobacteria;D_3__PB19;D_4__uncultured bacterium;D_5__uncultured bacterium;D_6__uncultured bacterium
3845f987090ff4dc36b9cf58bb441e31	4	1	D_0__Bacteria;D_1__Bacteroidetes;D_2__Bacteroidia;D_3__Cytophagales;D_4__Bernardetiaceae;D_5__Bernardetia;Ambiguous_taxa
43573e6b5c36be0d5af77c5e699de989	4	0.97948175	D_0__Bacteria;D_1__Bacteroidetes;D_2__Bacteroidia;D_3__Chitinophagales;D_4__Saprospiraceae;D_5__uncultured
517ee1f407ca6aeaf0f3d5bca3149fe7	4	0.981330229	D_0__Bacteria;D_1__Bacteroidetes;D_2__Bacteroidia;D_3__Flavobacteriales;D_4__Flavobacteriaceae;D_5__uncultured;D_6__uncultured Lutibacter sp.
56370f6c6a447928066d992816943e95	4	0.999885255	D_0__Bacteria;D_1__Bacteroidetes;D_2__Bacteroidia;D_3__Chitinophagales;D_4__Saprospiraceae;D_5__uncultured
5a5ac0fe46feb8370df8de977a20e5a7	4	0.999724897	D_0__Bacteria;D_1__Verrucomicrobia;D_2__Verrucomicrobiae;D_3__Opitutales;D_4__Puniceicoccaceae;D_5__Lentimonas
5ef16d5bc24d4bbf574ea171480b7344	4	0.790756239	D_0__Bacteria;D_1__Proteobacteria;D_2__Gammaproteobacteria;D_3__JTB23;D_4__uncultured bacterium;D_5__uncultured bacterium;D_6__uncultured bacterium
67b6fbaae40c0e6217ddd8ac577003ee	4	0.998606237	D_0__Bacteria;D_1__Actinobacteria;D_2__Acidimicrobiia;D_3__Microtrichales;D_4__Microtrichaceae;D_5__uncultured;Ambiguous_taxa
6ce459bb36d31e51ac2df23dfab7a5d5	4	0.955947538	D_0__Bacteria;D_1__Lentisphaerae;D_2__Lentisphaeria;D_3__Lentisphaerales;D_4__Lentisphaeraceae;D_5__Lentisphaera;D_6__uncultured organism
a9d2b445506e18c66020cf2399e3f98b	4	0.999997816	D_0__Bacteria;D_1__Proteobacteria;D_2__Gammaproteobacteria;D_3__Arenicellales;D_4__Arenicellaceae;D_5__HTCC5015;D_6__uncultured gamma proteobacterium
aa2b3a79f63097e06fb754a2d0cebbbc	4	0.780580098	D_0__Bacteria;D_1__Bacteroidetes;D_2__Bacteroidia;D_3__Chitinophagales;D_4__Saprospiraceae;D_5__Portibacter;D_6__uncultured bacterium
c49a43716ce4ca4d4029913af299067c	4	0.844460712	D_0__Bacteria;D_1__Proteobacteria;D_2__Gammaproteobacteria;D_3__Cellvibrionales;D_4__Spongiibacteraceae;D_5__BD1-7 clade
ce9458307297e216dd1e5e49a2a88c2a	4	0.999981121	D_0__Bacteria;D_1__Bacteroidetes;D_2__Bacteroidia;D_3__Cytophagales;D_4__Cyclobacteriaceae;D_5__uncultured;D_6__uncultured sediment bacterium
d423fb662fac55cc101e7eb3870b4a32	4	0.954661009	D_0__Bacteria;D_1__Proteobacteria;D_2__Gammaproteobacteria;D_3__Gammaproteobacteria Incertae Sedis;D_4__Unknown Family;D_5__Marinicella;D_6__uncultured Marinicella sp.
dc0ad7ea4f45a7c3f59c2b59d71587a8	4	0.992598105	D_0__Bacteria;D_1__Proteobacteria;D_2__Gammaproteobacteria;D_3__Oceanospirillales;D_4__Kangiellaceae;D_5__Aliikangiella;D_6__uncultured bacterium

e23e179b1541672770ccfb43f8a1b9ce	4	0.948864033	D_0__Bacteria;D_1__Proteobacteria;D_2__Gammaproteobacteria;D_3__SZB50;D_4__uncultured gamma proteobacterium;D_5__uncultured gamma proteobacterium;D_6__uncultured gamma [illegible]
ee5f06479b01c2f75b9e517ee7d66cf6	4	0.907797936	D_0__Bacteria;D_1__Bacteroidetes;D_2__Bacteroidia;D_3__Chitinophagales;D_4__Saprospiraceae;D_5__uncultured
074ad65a206ef6f821f59172e56026b8	3	0.997183526	D_0__Bacteria;D_1__Bacteroidetes;D_2__Bacteroidia;D_3__Chitinophagales;D_4__uncultured;D_5__uncultured Bacteroidetes bacterium;D_6__uncultured Bacteroidetes bacterium
09a3b1e276a31f3f9e9a2f524c4061ee	3	0.969171517	D_0__Bacteria;D_1__Bacteroidetes;D_2__Bacteroidia;D_3__Cytophagales;D_4__Amoebophilaceae;D_5__uncultured;D_6__uncultured bacterium
0a9245d394e32d7b5d84e593095b2546	3	0.982636334	D_0__Bacteria;D_1__Proteobacteria;D_2__Deltaproteobacteria;D_3__Myxococcales;D_4__Haliangiaceae;D_5__Haliangium;D_6__uncultured deep-sea bacterium
0c21eda91308d643a8f95e9588f2a6c6	3	0.926942772	D_0__Bacteria;D_1__Verrucomicrobia;D_2__Verrucomicrobiae;D_3__Verrucomicrobiales;D_4__Rubritaleaceae;D_5__Roseibacillus;D_6__uncultured marine bacterium
2e0ceaf33e571630ceabe35a0477615f	3	0.93838107	D_0__Bacteria;D_1__Proteobacteria;D_2__Gammaproteobacteria;D_3__Cellvibrionales;D_4__Halieaceae;D_5__Halioglobus
58b68861741a447896f6d30cad27d9d3	3	0.998106023	D_0__Bacteria;D_1__Bacteroidetes;D_2__Bacteroidia;D_3__Flavobacteriales;D_4__Crocinitomicaceae;D_5__uncultured
5a1f8148ba7c19ba89e32c426b660cd6	3	0.999991849	D_0__Bacteria;D_1__Bacteroidetes;D_2__Bacteroidia;D_3__Flavobacteriales;D_4__Flavobacteriaceae
6a6692d92947022df272e24c9a144103	3	0.999999054	D_0__Bacteria;D_1__Proteobacteria;D_2__Deltaproteobacteria;D_3__Myxococcales;D_4__Sandaracinaceae;D_5__uncultured
6bbf35251ac5a1cfc73af04e85db50cb	3	0.999712485	D_0__Bacteria;D_1__Proteobacteria;D_2__Gammaproteobacteria;D_3__Cellvibrionales;D_4__Halieaceae
941c1066287d07859a05174f31a3e849	3	1	D_0__Bacteria;D_1__Bacteroidetes;D_2__Bacteroidia;D_3__Flavobacteriales;D_4__Flavobacteriaceae
9b5dd8d46eba3fc4707705d66920f305	3	0.999934201	D_0__Bacteria;D_1__Proteobacteria;D_2__Deltaproteobacteria;D_3__Myxococcales;D_4__bacteriap25;D_5__uncultured organism;D_6__uncultured organism
a24267bca26fc178bbca1e25214bdb57	3	0.926133483	D_0__Bacteria;D_1__Proteobacteria;D_2__Deltaproteobacteria;D_3__Bdellovibrionales;D_4__Bacteriovoracaceae;D_5__uncultured
a31910efc6ad722aa8c523b23ca17804	3	0.99999998	D_0__Bacteria;D_1__Epsilonbacteraeota;D_2__Campylobacteria;D_3__Campylobacterales;D_4__Arcobacteraceae;D_5__Arcobacter
a4d5dc6c404d37f861cb0afc8e95ef47	3	0.978829634	D_0__Bacteria;D_1__Bacteroidetes;D_2__Bacteroidia;D_3__Flavobacteriales;D_4__Crocinitomicaceae;D_5__Crocinitomix;D_6__Crocinitomix sp. 0182

a611520840b0df74f3f4bbfa3f24871c	3	0.999998031	D_0__Bacteria;D_1__Proteobacteria;D_2__Alphaproteobacteria;D_3__Caulobacterales;D_4__Hyphomonadaceae;D_5__Hellea
a710bbf3598674a0c4d4cf608c945d39	3	0.908165943	D_0__Bacteria;D_1__Proteobacteria;D_2__Gammaproteobacteria;D_3__Cellvibrionales;D_4__Halieaceae;D_5__Halioglobus
ac37cb56a2f60c859c6128a387dd001e	3	0.987476292	D_0__Bacteria;D_1__Proteobacteria;D_2__Deltaproteobacteria;D_3__Myxococcales;D_4__Eel-36e1D6
b2c9bc9bdda9971f1a930b5232f6efe4	3	0.870096016	D_0__Bacteria;D_1__Bacteroidetes;D_2__Bacteroidia;D_3__Cytophagales;D_4__Cyclobacteriaceae;D_5__uncultured;Ambiguous_taxa
b6620e454c0522825a483adb3345b757	3	0.747383685	D_0__Bacteria;D_1__Bacteroidetes;D_2__Bacteroidia;D_3__Flavobacteriales;D_4__Cryomorphaceae;D_5__uncultured;D_6__uncultured Bacteroidetes bacterium
ca3153e68e47da6ec5023c2a5d78bf08	3	0.994399661	D_0__Bacteria;D_1__Proteobacteria;D_2__Deltaproteobacteria;D_3__NB1-j;Ambiguous_taxa;Ambiguous_taxa;Ambiguous_taxa
e28011584ccaced1fa94c55ac529a128	3	0.99205407	D_0__Bacteria;D_1__Bacteroidetes;D_2__Bacteroidia;D_3__Flavobacteriales;D_4__Flavobacteriaceae;D_5__Ulvibacter;D_6__uncultured bacterium
f40c07cafcd0f9a8e2e848462ebbca32	3	0.880890278	D_0__Bacteria;D_1__Proteobacteria;D_2__Gammaproteobacteria;D_3__Cellvibrionales;D_4__Halieaceae;D_5__Halioglobus;D_6__Saccharina japonica
fbded9582f1e981500cbcd900bf9385e	3	0.972015858	D_0__Bacteria;D_1__Chloroflexi;D_2__Anaerolineae;D_3__Ardenticatenales;D_4__uncultured;D_5__uncultured bacterium;D_6__uncultured bacterium
22be5673e01d15edae6f2de3cbb8a863	2	1	D_0__Bacteria;D_1__Proteobacteria;D_2__Deltaproteobacteria;D_3__Myxococcales;D_4__Nannocystaceae
3b9747f15c0ed6fcfbcb2af0a44eb442	2	0.997271682	D_0__Bacteria;D_1__Patescibacteria;D_2__Gracilibacteria;D_3__JGI 0000069-P22;D_4__uncultured bacterium;D_5__uncultured bacterium;D_6__uncultured bacterium
4477483870925dfa3787975cb7eec08d	2	0.999982821	D_0__Bacteria;D_1__WPS-2;D_2__uncultured bacterium;D_3__uncultured bacterium;D_4__uncultured bacterium;D_5__uncultured bacterium;D_6__uncultured bacterium
44ce77063de0e615def7f4be6e01e343	2	0.999892149	D_0__Bacteria;D_1__Patescibacteria;D_2__Parcubacteria;D_3__Candidatus Moranbacteria;D_4__uncultured bacterium;D_5__uncultured bacterium;D_6__uncultured bacterium
4da426668e8d9202c8111628a29bbaf0	2	0.764358193	D_0__Bacteria;D_1__Proteobacteria;D_2__Gammaproteobacteria;D_3__Cellvibrionales;D_4__Halieaceae;D_5__OM60(NOR5) clade
7a7340214845e07ec920c4dedae937f9	2	0.967069786	D_0__Bacteria;D_1__Proteobacteria;D_2__Deltaproteobacteria;D_3__PB19;D_4__uncultured deep-sea bacterium;D_5__uncultured deep-sea bacterium;D_6__uncultured deep-sea bacterium
7b17bc11e0abe0e899d2f8c0e1db5e4b	2	0.996876944	D_0__Bacteria;D_1__Proteobacteria;D_2__Gammaproteobacteria;D_3__UBA10353 marine group

7c2f78382155f501228a4731b2b438d3	2	0.954139161	D_0__Bacteria;D_1__Bacteroidetes;D_2__Bacteroidia;D_3__Flavobacteriales;D_4__Schleiferiaceae;D_5__Schleiferia;Ambiguous_taxa
87eafc99da738900dd2b657c8fd74fcb	2	0.999999917	D_0__Bacteria;D_1__Proteobacteria;D_2__Gammaproteobacteria
880f3181259f4029660b3b21d501dbc3	2	0.999999266	D_0__Bacteria;D_1__Bacteroidetes;D_2__Bacteroidia;D_3__Cytophagales;D_4__Amoebophilaceae;D_5__uncultured
9675509ad27b39a83dfd976fce0560d9	2	0.760972754	D_0__Bacteria;D_1__Proteobacteria;D_2__Gammaproteobacteria;D_3__Alteromonadales;D_4__Colwelliaceae;D_5__Colwellia
9c68652c784c1a82bf0485a5e8738442	2	0.999896557	D_0__Bacteria;D_1__Proteobacteria;D_2__Alphaproteobacteria;D_3__Micavibrionales;D_4__Micavibrionaceae;D_5__uncultured
9c6878ab573f961608ddc1c858109612	2	0.997362908	D_0__Bacteria;D_1__Proteobacteria;D_2__Alphaproteobacteria;D_3__Rhizobiales;D_4__Rhizobiales Incertae Sedis;D_5__Anderseniella;Ambiguous_taxa
a7a7c6fa482957f1e6ddb23fc660d5f3	2	0.999999113	D_0__Bacteria;D_1__Proteobacteria;D_2__Deltaproteobacteria;D_3__Bdellovibrionales;D_4__Bdellovibrionaceae;D_5__OM27 clade
aa1c1a111e0916528fda6ff7c22f7114	2	0.991078304	D_0__Bacteria;D_1__Proteobacteria;D_2__Deltaproteobacteria;D_3__Desulfobacterales;D_4__Desulfobacteraceae;D_5__Desulfosarcina;Ambiguous_taxa
ae8eee9443d863f4aa9ac72bcacfc227	2	0.800858152	D_0__Bacteria;D_1__Proteobacteria;D_2__Deltaproteobacteria;D_3__Bdellovibrionales;D_4__Bdellovibrionaceae;D_5__Bdellovibrio;D_6__uncultured Bdellovibrio sp.
e63851ec4add7c00cf1c58139653ec21	2	0.999822933	D_0__Bacteria;D_1__Proteobacteria;D_2__Gammaproteobacteria;D_3__Oceanospirillales;D_4__Endozoicomonadaceae;D_5__Endozoicomonas
f90a8e630b8cfddff29b353813409ab8	2	0.795486921	D_0__Bacteria;D_1__Bacteroidetes;D_2__Bacteroidia;D_3__Flavobacteriales;D_4__Flavobacteriaceae;D_5__Aquibacter
2f5bd330cf65bda9028181c7feef7a23	1	0.999998871	D_0__Bacteria;D_1__Proteobacteria;D_2__Gammaproteobacteria
35867c74d4e209233847681dda7de645	1	0.999999974	D_0__Bacteria;D_1__Proteobacteria;D_2__Gammaproteobacteria
4591377c6d349960df79cbe9b7ab95fd	1	0.958580077	D_0__Bacteria;D_1__Proteobacteria;D_2__Gammaproteobacteria;D_3__Oceanospirillales;D_4__Nitrincolaceae;D_5__uncultured
47307b8283c3614e9c0f7b9b3cf52d58	1	1	D_0__Bacteria
5aa19433dc70e7af8aebc0a78b63bd23	1	0.999999969	D_0__Bacteria;D_1__Proteobacteria;D_2__Gammaproteobacteria

5bcd674deba252800b4c2be7701df8b6	1	0.999999929	D_0__Bacteria;D_1__Proteobacteria;D_2__Gammaproteobacteria
6286607563def4d7f3f316c248a7ac6f	1	0.999999935	D_0__Bacteria;D_1__Proteobacteria;D_2__Gammaproteobacteria
687ba53d93ea3e07b6981e10390fc78b	1	0.999990119	D_0__Bacteria;D_1__Bacteroidetes;D_2__Bacteroidia;D_3__Flavobacteriales;D_4__Flavobacteriaceae
747cdd0a60ca9e9549af2ddd311a6c64	1	0.826414913	D_0__Bacteria;D_1__Proteobacteria;D_2__Gammaproteobacteria;D_3__Oceanospirillales;D_4__Nitrincolaceae;D_5__uncultured;D_6__uncultured marine bacterium
a818fc81c896c24ffe175bc98a6ae8dd	1	0.996232638	D_0__Bacteria;D_1__Proteobacteria;D_2__Gammaproteobacteria;D_3__Ectothiorhodospirales;D_4__Ectothiorhodospiraceae;D_5__uncultured;D_6__uncultured bacterium
ad00a990773ccc085390a8d9b1cd2f41	1	0.881892521	D_0__Bacteria;D_1__Proteobacteria;D_2__Gammaproteobacteria;D_3__Alteromonadales;D_4__Alteromonadaceae
af9d479f4b6256d0f59da90f64eea627	1	0.993884199	D_0__Bacteria;D_1__Proteobacteria;D_2__Gammaproteobacteria;D_3__HOC36;D_4__uncultured bacterium;D_5__uncultured bacterium;D_6__uncultured bacterium
c84414b510ae2af8c21836a2894103f7	1	0.949076794	D_0__Bacteria;D_1__Proteobacteria;D_2__Gammaproteobacteria;D_3__Cellvibrionales;D_4__Spongiibacteraceae;D_5__BD1-7 clade;D_6__uncultured marine bacterium
cf60aa59052b108edf6c929c9b44b86c	1	0.999999926	D_0__Bacteria;D_1__Proteobacteria;D_2__Gammaproteobacteria
d417db562ab35fc0d490d7a8a4bf79cc	1	0.835030433	D_0__Bacteria;D_1__Proteobacteria;D_2__Gammaproteobacteria;D_3__Betaproteobacteriales;D_4__Nitrosomonadaceae
ddb65b3fec98525b373f8b9a77fbdeb5	1	0.965812289	D_0__Bacteria;D_1__Proteobacteria;D_2__Gammaproteobacteria;D_3__Cellvibrionales;D_4__Spongiibacteraceae;D_5__BD1-7 clade;D_6__uncultured marine bacterium
febe025e22663643e5493464c040c114	1	0.836770999	D_0__Bacteria;D_1__Proteobacteria;D_2__Gammaproteobacteria;D_3__Oceanospirillales;D_4__Nitrincolaceae;D_5__uncultured;Ambiguous_taxa

Table S6. ASVs found from *H.* (*Rhizoniera*) *dancoi* with percentage of confidence ≥ 75%.

Feature ID	Number of sequences	Confidence	Taxon
8101484a5c241eb9197f3f5ed275e28d	3374	0.999999995	D_0__Bacteria;D_1__Proteobacteria;D_2__Alphaproteobacteria;D_3__Rhodobacterales;D_4__Rhodobacteraceae
bd490e3ab22353eb9055a0cd610e6b7b	1627	0.999979753	D_0__Bacteria;D_1__Proteobacteria;D_2__Gammaproteobacteria;D_3__UBA10353 marine group
66144bd48db1b642593c979327676e71	469	0.803096292	D_0__Bacteria;D_1__Nitrospinae;D_2__Nitrospinia;D_3__Nitrospinales;D_4__Nitrospinaceae;D_5__LS-NOB;D_6__uncultured delta proteobacterium
8d47cc06557e4eef496df29414b3dae0	406	0.964964319	D_0__Bacteria;D_1__Proteobacteria;D_2__Gammaproteobacteria;D_3__JTB23;D_4__hydrothermal vent metagenome;D_5__hydrothermal vent metagenome;D_6__hydrothermal vent metagenome
236cac611fc74c2387f52bce68880c0e	386	0.990036375	D_0__Bacteria;D_1__Proteobacteria;D_2__Gammaproteobacteria;D_3__Betaproteobacteriales;D_4__Methylophilaceae;D_5__Methylotenera
818d9fcef920f3637dd4f68382634c0e	284	0.99941094	D_0__Bacteria;D_1__Actinobacteria;D_2__Acidimicrobiia;D_3__Microtrichales;D_4__Microtrichaceae;D_5__Sva0996 marine group;D_6__uncultured organism
2ababd6d22c8aad22fa045bbcaa423bc	282	0.907056143	D_0__Bacteria;D_1__Proteobacteria;D_2__Alphaproteobacteria;D_3__Thalassobaculales;D_4__Nisaeaceae;D_5__OM75 clade;D_6__uncultured alpha proteobacterium
178de13f8345fa4ee0d6a84777ccb705	247	0.990355421	D_0__Bacteria;D_1__Proteobacteria;D_2__Gammaproteobacteria;D_3__Thiomicrospirales;D_4__Thioglobaceae;D_5__SUP05 cluster;D_6__uncultured bacterium
3d1a14e152a9397daebe6259de1c85b9	199	0.999696372	D_0__Bacteria;D_1__Actinobacteria;D_2__Acidimicrobiia;D_3__Microtrichales;D_4__Microtrichaceae;D_5__Sva0996 marine group;D_6__uncultured bacterium
965a7a8086d78a368dd6bccc35429e79	133	0.930569131	D_0__Bacteria;D_1__Proteobacteria;D_2__Alphaproteobacteria;D_3__uncultured
148e426c8329a83fc5e43afaacdcc2c2	121	0.999999226	D_0__Bacteria;D_1__Proteobacteria;D_2__Gammaproteobacteria;D_3__Oceanospirillales;D_4__Pseudohongiellaceae;D_5__Pseudohongiella
063a62980037e31b544a1f92e34a032b	94	0.98905359	D_0__Bacteria;D_1__Proteobacteria;D_2__Gammaproteobacteria;D_3__Betaproteobacteriales;D_4__Methylophilaceae;D_5__Methylotenera
9e7cd2e390b97196123849b615bc466c	80	0.998380182	D_0__Bacteria
91eb9562fc7aefa38568e96822db8f4b	78	0.941782157	D_0__Bacteria;D_1__Proteobacteria;D_2__Alphaproteobacteria;D_3__Rhodospirillales;D_4__Magnetospiraceae;D_5__uncultured;D_6__uncultured bacterium

109f4a96b21eb6c1f3349132c4ffe186	66	0.755923215	D_0__Bacteria;D_1__Proteobacteria;D_2__Gammaproteobacteria;D_3__Cellvibrionales;D_4__Spongiibacteraceae;D_5__BD1-7 clade;Ambiguous_taxa
86c4313b7a3946068e32ca211314c4a6	58	0.979720802	D_0__Bacteria;D_1__Proteobacteria;D_2__Gammaproteobacteria;D_3__Cellvibrionales;D_4__Spongiibacteraceae;D_5__BD1-7 clade
d3bc04a05bcab8a9d527fe3c946dfdee	53	0.842346275	D_0__Bacteria;D_1__Bacteroidetes;D_2__Bacteroidia;D_3__Cytophagales;D_4__Cyclobacteriaceae;D_5__Ekhidna;Ambiguous_taxa
dcef5e9d4283900ba2cd2b66ab14894b	53	0.957789518	D_0__Bacteria;D_1__Proteobacteria;D_2__Alphaproteobacteria;D_3__uncultured;D_4__uncultured bacterium;D_5__uncultured bacterium;D_6__uncultured bacterium
5d9835c028d02be320fdd0c95eb23f4f	36	0.82943598	D_0__Bacteria;D_1__Bacteroidetes;D_2__Bacteroidia;D_3__Flavobacteriales;D_4__Flavobacteriaceae;D_5__Polaribacter 1;Ambiguous_taxa
2fe8d6838909663a039294cf48ea3720	21	1	D_0__Bacteria;D_1__Firmicutes;D_2__Clostridia;D_3__Clostridiales;D_4__Peptostreptococcaceae;D_5__Peptostreptococcus
ee0f5caff94365896ef095e26d1fed6d	21	0.749932806	D_0__Bacteria;D_1__Proteobacteria;D_2__Alphaproteobacteria;D_3__SAR11 clade;D_4__Clade I;D_5__Clade Ia
2ecd7cdde1ebb615fe5b7d0bf14ff1d3	16	0.982377708	D_0__Bacteria;D_1__Proteobacteria;D_2__Deltaproteobacteria;D_3__Bdellovibrionales;D_4__Bdellovibrionaceae;D_5__Bdellovibrio;D_6__uncultured bacterium
56ef94fb3e414b8ddcfbb92d223464b7	14	0.999748562	D_0__Bacteria;D_1__Proteobacteria;D_2__Alphaproteobacteria;D_3__Rhodobacterales;D_4__Rhodobacteraceae
5171c7c84b18d54ac99984e3fc0bc2f6	13	0.8955098	D_0__Bacteria;D_1__Fusobacteria;D_2__Fusobacteriia;D_3__Fusobacteriales;D_4__Fusobacteriaceae;D_5__Fusobacterium;Ambiguous_taxa
ccb67f3a797ef786dc0c42cf3b8bd220	13	0.999963971	D_0__Bacteria;D_1__Proteobacteria;D_2__Gammaproteobacteria;D_3__Alteromonadales;D_4__Colwelliaceae;D_5__Colwellia
ce42b14845e10ad958ca1cb53045dbca	12	0.995966837	D_0__Bacteria;D_1__Proteobacteria;D_2__Gammaproteobacteria;D_3__Alteromonadales;D_4__Colwelliaceae;D_5__Colwellia
da945cd94d83cad51bfcd0c5d26176a9	12	0.994989502	D_0__Bacteria;D_1__Proteobacteria;D_2__Gammaproteobacteria;D_3__Arenicellales;D_4__Arenicellaceae;D_5__Arenicella;D_6__uncultured bacterium
ece89bff980b08b04d6f8dbe287592c3	11	0.999999069	D_0__Bacteria;D_1__Epsilonbacteraeota;D_2__Campylobacteria;D_3__Campylobacterales;D_4__Arcobacteraceae;D_5__Arcobacter
cfb8795bebf1b9c511f8939312c10d75	8	0.99808824	D_0__Bacteria;D_1__Planctomycetes;D_2__Phycisphaerae;D_3__Phycisphaerales;D_4__Phycisphaeraceae;D_5__Phycisphaera
67f51937ae1fb923adccee019281e23a	7	0.967116949	D_0__Bacteria;D_1__Bacteroidetes;D_2__Bacteroidia;D_3__Bacteroidales;D_4__Bacteroidaceae;D_5__Bacteroides;D_6__uncultured bacterium

eb95a4e7fc05db5f925d4c8ca8ff01b8	7	1	D_0__Bacteria;D_1__Bacteroidetes;D_2__Bacteroidia;D_3__Bacteroidales;D_4__Prevotellaceae;D_5__Alloprevotella
061ebd61b6e228fb835fa95b48c4fef5	6	0.781049076	D_0__Bacteria;D_1__Proteobacteria;D_2__Gammaproteobacteria;D_3__Arenicellales;D_4__Arenicellaceae;D_5__uncultured;Ambiguous_taxa
6abb852c91aab4f588254187c4d86f7b	6	0.784569604	D_0__Bacteria;D_1__Proteobacteria;D_2__Gammaproteobacteria;D_3__UBA10353 marine group
7874f80382e597a6eb3bf95a6defd394	5	0.875967844	D_0__Bacteria;D_1__Fusobacteria;D_2__Fusobacteriia;D_3__Fusobacteriales;D_4__Fusobacteriaceae;D_5__Psychrilyobacter;D_6__uncultured bacterium
b45ad382acdfdda9b261dd9f408b9a41	5	0.891074376	D_0__Bacteria;D_1__Fusobacteria;D_2__Fusobacteriia;D_3__Fusobacteriales;D_4__Fusobacteriaceae;D_5__Cetobacterium;D_6__uncultured bacterium
e1b000731bf1b4ab30332e90d5da12fd	5	0.984201195	D_0__Bacteria;D_1__Bacteroidetes;D_2__Bacteroidia;D_3__Bacteroidales;D_4__Rikenellaceae;D_5__Alistipes;D_6__uncultured bacterium
04e1d5fe2db1e05d3f7464d881e335db	4	0.999999631	D_0__Bacteria;D_1__Epsilonbacteraeota;D_2__Campylobacteria;D_3__Campylobacterales;D_4__Arcobacteraceae;D_5__Arcobacter
0aebc7c8b90e3922ae2960d6bc6ba3f8	4	0.968351558	D_0__Bacteria;D_1__Proteobacteria;D_2__Gammaproteobacteria;D_3__Alteromonadales;D_4__Moritellaceae;D_5__Moritella;Ambiguous_taxa
775cf63863803484cc269b57f8480f06	4	0.793784399	D_0__Bacteria;D_1__Proteobacteria;D_2__Gammaproteobacteria;D_3__Tenderiales;D_4__Tenderiaceae;D_5__Candidatus Tenderia;D_6__uncultured gamma proteobacterium
d8661c97e95673d5ce39a8660bd77e1f	4	0.969751856	D_0__Bacteria
f8138d2dc2b8ea0acdb8fa121bb28fa3	4	0.977022449	D_0__Bacteria;D_1__Firmicutes;D_2__Clostridia;D_3__Clostridiales;D_4__Clostridiaceae 1;D_5__Clostridium sensu stricto 1
177920551a2a705a66e2f4a2663d4561	3	0.927689543	D_0__Bacteria;D_1__Bacteroidetes;D_2__Bacteroidia;D_3__Flavobacteriales;D_4__Cryomorphaceae;D_5__uncultured;D_6__marine metagenome
1db50f814d8a99be5666486f4a4de024	3	0.999094451	D_0__Bacteria;D_1__Bacteroidetes;D_2__Bacteroidia;D_3__Flavobacteriales;D_4__Flavobacteriaceae;D_5__NS2b marine group
588712d3aea224fb95ee4119a7100b98	3	0.999931918	D_0__Bacteria;D_1__Bacteroidetes;D_2__Bacteroidia;D_3__Flavobacteriales;D_4__NS9 marine group;D_5__seawater metagenome;D_6__seawater metagenome
67c9e284c7de43eb5596d0b6434a57f8	3	0.759345136	D_0__Bacteria;D_1__Bacteroidetes;D_2__Bacteroidia;D_3__Bacteroidales;D_4__Prevotellaceae;D_5__Alloprevotella;D_6__gut metagenome
8a4c5faa1ab7f0561f9124015ffebe4d	3	0.981544224	D_0__Bacteria;D_1__Proteobacteria;D_2__Gammaproteobacteria;D_3__Oceanospirillales;D_4__Nitrincolaceae;D_5__uncultured;D_6__uncultured marine bacterium

bca6f8c038dcda27a706e3ae8f739a85	3	0.99999991	D_0__Bacteria;D_1__Patescibacteria;D_2__Parcubacteria;D_3__Candidatus Campbellbacteria;D_4__uncultured bacterium;D_5__uncultured bacterium;D_6__uncultured bacterium
cc1a20baae9c705d4ce4ddc21cd48ecb	3	0.847305238	D_0__Bacteria;D_1__Firmicutes;D_2__Clostridia;D_3__Clostridiales;D_4__Ruminococcaceae;D_5__Butyricicoccus;Ambiguous_taxa
d942bac17b1a69d8499fc1ac7c8b4dac	3	0.984858379	D_0__Bacteria;D_1__Bacteroidetes;D_2__Bacteroidia;D_3__Bacteroidales;D_4__Marinifilaceae;D_5__Marinifilum;D_6__uncultured Bacteroidetes bacterium
8b4ba85d891e756d6829fb6162686bcf	2	0.996213196	D_0__Bacteria;D_1__Proteobacteria;D_2__Gammaproteobacteria;D_3__Oceanospirillales;D_4__Saccharospirillaceae;D_5__Oleispira;Ambiguous_taxa
be66204e7b2c9ca0be77a78be57aeb58	2	0.952091125	D_0__Bacteria;D_1__Actinobacteria;D_2__Coriobacteriia;D_3__Coriobacteriales;D_4__Coriobacteriales Incertae Sedis;D_5__uncultured;D_6__uncultured bacterium
daff3b4904237890080a447c6dfe4d40	2	0.832359493	D_0__Bacteria;D_1__Patescibacteria;D_2__Gracilibacteria;D_3__JGI 0000069-P22;D_4__uncultured bacterium;D_5__uncultured bacterium;D_6__uncultured bacterium
295e65e6893355570920c42e3da53739	1	0.999935057	D_0__Bacteria;D_1__Proteobacteria;D_2__Gammaproteobacteria;D_3__Alteromonadales;D_4__Alteromonadaceae
4da211ba6ea0339b60009f074cc4d0e0	1	0.783727801	D_0__Bacteria;D_1__Actinobacteria;D_2__Coriobacteriia;D_3__Coriobacteriales;D_4__Atopobiaceae;D_5__Atopobium;D_6__uncultured Coriobacteriaceae bacterium
fb3df40abc0b26682babb06ac498989f	1	0.963122268	D_0__Bacteria;D_1__Proteobacteria;D_2__Gammaproteobacteria;D_3__Alteromonadales;D_4__Alteromonadaceae

Table S7. ASVs found from *H. pilosus* with percentage of confidence ≥ 75%.

Feature ID	Number of sequences	Confidence	Taxon
6a7e066c3fad35a215e4104aa89bff4a	3882	0.977613476	D_0__Bacteria;D_1__Bacteroidetes;D_2__Bacteroidia;D_3__Flavobacteriales;D_4__Flavobacteriaceae; D_5__NS5 marine group;Ambiguous_taxa
b8e89b31d202d0f91db56a853e8fa35e	1934	0.999992535	D_0__Bacteria;D_1__Proteobacteria;D_2__Alphaproteobacteria;D_3__Caulobacterales;D_4__Hyphom onadaceae;D_5__Robiginitomaculum
00a80fb2e5b6d10a531c341927651e92	1130	0.75514023	D_0__Bacteria;D_1__Proteobacteria;D_2__Alphaproteobacteria;D_3__Rhodobacterales;D_4__Rhodob acteraceae;D_5__Roseobacter clade NAC11-7 lineage;D_6__uncultured alpha proteobacterium
a660e09cb7abcf685efa1407a1e7ac81	1118	0.999999997	D_0__Bacteria;D_1__Proteobacteria;D_2__Alphaproteobacteria;D_3__Rhodobacterales;D_4__Rhodob acteraceae
e4887289324a32b29b9cd5f9f2945d84	1052	0.999999846	D_0__Bacteria;D_1__Proteobacteria;D_2__Gammaproteobacteria;D_3__Oceanospirillales;D_4__Pseu dohongiellaceae;D_5__Pseudohongiella
7e3a7538d28877279afb650ad72e063c	559	0.998358455	D_0__Bacteria;D_1__Proteobacteria;D_2__Gammaproteobacteria;D_3__KI89A clade;Ambiguous_taxa;Ambiguous_taxa;Ambiguous_taxa
775cf63863803484cc269b57f8480f06	544	0.793784399	D_0__Bacteria;D_1__Proteobacteria;D_2__Gammaproteobacteria;D_3__Tenderiales;D_4__Tenderiace ae;D_5__Candidatus Tenderia;D_6__uncultured gamma proteobacterium
d83bf1a83ba09c470f55f6c15cfe1a4a	419	0.871432659	D_0__Archaea;D_1__Thaumarchaeota;D_2__Nitrososphaeria;D_3__Nitrosopumilales;D_4__Nitrosopu milaceae;D_5__Candidatus Nitrosopumilus
9ed5ea3d900ceda3d4b386816ee4ef92	396	0.999999968	D_0__Bacteria;D_1__Proteobacteria;D_2__Deltaproteobacteria;D_3__Bdellovibrionales;D_4__Bdellovi brionaceae;D_5__Bdellovibrio
508baf54718ae7094689191a66899ba3	294	0.999991272	D_0__Bacteria;D_1__Proteobacteria;D_2__Gammaproteobacteria;D_3__UBA10353 marine group
155289cff466171178063805d2a3319b	280	0.909067567	D_0__Bacteria;D_1__Proteobacteria;D_2__Gammaproteobacteria;D_3__Steroidobacterales;D_4__Wo eseiaceae;D_5__Woeseia
6cbaf2a2e24d8dc1a6e13b3bd71bce78	228	0.971862011	D_0__Bacteria;D_1__Proteobacteria;D_2__Gammaproteobacteria;D_3__JTB23
2401cfdd7aae2d2977a422acc798c0b9	213	0.999044233	D_0__Bacteria;D_1__Actinobacteria;D_2__Acidimicrobiia;D_3__Microtrichales;D_4__Microtrichaceae; D_5__Sva0996 marine group;D_6__uncultured organism
dc898434231409883e60c022df816362	199	0.999699272	D_0__Bacteria;D_1__Proteobacteria;D_2__Gammaproteobacteria;D_3__Cellvibrionales

8f5b60e2ef54d12840bd47faa5616cab	191	0.963474653	D_0__Bacteria;D_1__Proteobacteria;D_2__Alphaproteobacteria;D_3__Thalassobaculales;D_4__Nisaeaceae;D_5__OM75 clade;D_6__uncultured alpha proteobacterium
ec145354454885b65f8fa2a7d9464543	76	0.972176791	D_0__Bacteria;D_1__Bacteroidetes;D_2__Bacteroidia;D_3__Flavobacteriales;D_4__Flavobacteriaceae;D_5__NS5 marine group;Ambiguous_taxa
e9ac73db852dad95a70f25e4f63b8e1a	64	0.999990894	D_0__Bacteria;D_1__Proteobacteria;D_2__Alphaproteobacteria;D_3__Caulobacterales;D_4__Hyphomonadaceae;D_5__Robiginitomaculum
b593eca7682213ae9045b637757739a5	61	0.999999943	D_0__Bacteria;D_1__Proteobacteria;D_2__Deltaproteobacteria;D_3__Bdellovibrionales;D_4__Bdellovibrionaceae;D_5__Bdellovibrio
7c1be5aa888860516751b219fb83ece8	50	0.78800496	D_0__Bacteria;D_1__Proteobacteria;D_2__Alphaproteobacteria;D_3__Rhodobacterales;D_4__Rhodobacteraceae;D_5__Roseobacter clade NAC11-7 lineage;D_6__uncultured alpha proteobacterium
53ed70e9e9cb94365506e1cb76933fa0	48	0.992509086	D_0__Bacteria
ee0f5caff94365896ef095e26d1fed6d	48	0.749932806	D_0__Bacteria;D_1__Proteobacteria;D_2__Alphaproteobacteria;D_3__SAR11 clade;D_4__Clade I;D_5__Clade Ia
16ac7540cf6e429f02f3ec1921bc4e22	45	0.999999996	D_0__Bacteria;D_1__Proteobacteria;D_2__Alphaproteobacteria;D_3__Rhodobacterales;D_4__Rhodobacteraceae
5d9835c028d02be320fdd0c95eb23f4f	32	0.82943598	D_0__Bacteria;D_1__Bacteroidetes;D_2__Bacteroidia;D_3__Flavobacteriales;D_4__Flavobacteriaceae;D_5__Polaribacter 1;Ambiguous_taxa
74818e9c275df1d2842b9d4ef8ae3fc5	32	0.995895467	D_0__Bacteria;D_1__Proteobacteria;D_2__Gammaproteobacteria;D_3__KI89A clade;Ambiguous_taxa;Ambiguous_taxa;Ambiguous_taxa
8547a0a71e8c173be5e253364f24957d	16	0.999999836	D_0__Bacteria;D_1__Patescibacteria;D_2__Parcubacteria;D_3__Candidatus Campbellbacteria;D_4__uncultured bacterium;D_5__uncultured bacterium;D_6__uncultured [illegible]
dfb9078f726f9b239561ae7a04e4c445	15	0.997416652	D_0__Bacteria;D_1__Proteobacteria;D_2__Gammaproteobacteria;D_3__Oceanospirillales;D_4__Nitrincolaceae;D_5__uncultured;D_6__uncultured marine bacterium
422b267b273b1af947e80657c47c8072	13	0.999501022	D_0__Bacteria;D_1__Lentisphaerae;D_2__Lentisphaeria;D_3__Lentisphaerales;D_4__Lentisphaeraceae;D_5__Lentisphaera
ce42b14845e10ad958ca1cb53045dbca	12	0.995966837	D_0__Bacteria;D_1__Proteobacteria;D_2__Gammaproteobacteria;D_3__Alteromonadales;D_4__Colwelliaceae;D_5__Colwellia
085f909fd9a9edf1a0bbd3798cf5279b	8	0.887600266	D_0__Bacteria;D_1__Proteobacteria;D_2__Gammaproteobacteria;D_3__Thiomicrospirales;D_4__Thioglobaceae;D_5__SUP05 cluster;D_6__uncultured marine bacterium
ece89bff980b08b04d6f8dbe287592c3	7	0.999999069	D_0__Bacteria;D_1__Epsilonbacteraeota;D_2__Campylobacteria;D_3__Campylobacterales;D_4__Arcobacteraceae;D_5__Arcobacter

ed347145ab935ef912e1f90fe9590298	7	0.886294614	D_0__Bacteria;D_1__Proteobacteria;D_2__Gammaproteobacteria;D_3__Alteromonadales;D_4__Colwelliaceae;D_5__Colwellia
25931e17ea6c6d8fd57a5230fec2c625	5	0.939286821	D_0__Bacteria;D_1__Proteobacteria;D_2__Alphaproteobacteria;D_3__Parvibaculales;D_4__PS1 clade;D_5__uncultured bacterium;D_6__uncultured bacterium
54397cb35127b6a5df751155b7461547	4	0.748330375	D_0__Bacteria;D_1__Proteobacteria;D_2__Gammaproteobacteria;D_3__Cellvibrionales;D_4__Porticoccaceae;D_5__SAR92 clade;D_6__uncultured marine bacterium
177920551a2a705a66e2f4a2663d4561	3	0.927689543	D_0__Bacteria;D_1__Bacteroidetes;D_2__Bacteroidia;D_3__Flavobacteriales;D_4__Cryomorphaceae;D_5__uncultured;D_6__marine metagenome
1db50f814d8a99be5666486f4a4de024	3	0.999094451	D_0__Bacteria;D_1__Bacteroidetes;D_2__Bacteroidia;D_3__Flavobacteriales;D_4__Flavobacteriaceae;D_5__NS2b marine group
3dfd78ec33126f07b602cf3e31bbde97	3	0.998655009	D_0__Bacteria;D_1__Proteobacteria;D_2__Gammaproteobacteria;D_3__Alteromonadales;D_4__Alteromonadaceae;D_5__Paraglaciecola;Ambiguous_taxa
9646e8e963bfa6d3658a2e4db796657f	3	0.85862286	D_0__Bacteria;D_1__Proteobacteria;D_2__Gammaproteobacteria;D_3__Alteromonadales;D_4__Colwelliaceae;D_5__Colwellia
ba99a570c2761e419c443a97c0ea39d3	3	0.999999975	D_0__Bacteria;D_1__Epsilonbacteraeota;D_2__Campylobacteria;D_3__Campylobacterales;D_4__Arcobacteraceae;D_5__Arcobacter
28708382f55f006ce34f01206e5abd54	2	0.978943092	D_0__Bacteria
d50919d54541b225899cc06110d14e29	2	0.994562885	D_0__Bacteria;D_1__Bacteroidetes;D_2__Bacteroidia;D_3__Flavobacteriales;D_4__Cryomorphaceae;D_5__uncultured;D_6__uncultured Flavobacteriia bacterium
17ac0de31a0f390831256c95971fe063	1	0.999999997	D_0__Bacteria;D_1__Proteobacteria;D_2__Gammaproteobacteria
3dfb18f9278b81c2508361494d586e23	1	0.780506511	D_0__Bacteria;D_1__Proteobacteria;D_2__Gammaproteobacteria;D_3__Alteromonadales
5b1e245cd77376d116b32903bd8eb50e	1	0.808646049	D_0__Bacteria;D_1__Proteobacteria;D_2__Gammaproteobacteria;D_3__Alteromonadales;D_4__Moritellaceae
d08b4ba7275c9769d6cfefd220c24ac7	1	0.806919402	D_0__Bacteria;D_1__Proteobacteria;D_2__Gammaproteobacteria;D_3__Alteromonadales;D_4__Moritellaceae;D_5__Moritella
d689cd003ad6e8c86be92e1ac114ff9d	1	0.827327601	D_0__Bacteria;D_1__Proteobacteria;D_2__Alphaproteobacteria;D_3__Rhodobacterales;D_4__Rhodobacteraceae
db4c9169139fd731339514a79da0567a	1	0.826185459	D_0__Bacteria;D_1__Proteobacteria;D_2__Gammaproteobacteria;D_3__Alteromonadales;D_4__Moritellaceae;D_5__Moritella

Table S8. ASVs found from *M. sarai* with percentage of confidence ≥ 75%.

Feature ID	Number of sequences	Confidence	Taxon
3893f274e96d4cc70e9f44136fec7477	4605	0.790016759	D_0__Bacteria;D_1__Proteobacteria;D_2__Gammaproteobacteria;D_3__Betaproteobacteriales;D_4__EC94;D_5__uncultured bacterium;D_6__uncultured bacterium
c3267a32e1403cf02ac84957f2cbb902	1808	0.937720316	D_0__Bacteria;D_1__Proteobacteria;D_2__Gammaproteobacteria;D_3__Betaproteobacteriales;D_4__EC94;Ambiguous_taxa;Ambiguous_taxa
1d5a7a4ffdf2def18e8c51a31ac52022	1451	0.954210628	D_0__Bacteria;D_1__Proteobacteria;D_2__Gammaproteobacteria;D_3__Betaproteobacteriales;D_4__EC94;Ambiguous_taxa;Ambiguous_taxa
011cd7959322e1d5e2949938bfc0ed59	818	0.994228284	D_0__Bacteria
5d9835c028d02be320fdd0c95eb23f4f	612	0.82943598	D_0__Bacteria;D_1__Bacteroidetes;D_2__Bacteroidia;D_3__Flavobacteriales;D_4__Flavobacteriaceae;D_5__Polaribacter 1;Ambiguous_taxa
7bca951215ee55fad72ca4245f276361	477	0.98477904	D_0__Bacteria;D_1__Bacteroidetes;D_2__Bacteroidia;D_3__Cytophagales;D_4__Cyclobacteriaceae;D_5__Ekhidna;D_6__uncultured bacterium
983f6b74e25c3d36c9d9940508779742	325	0.999716167	D_0__Bacteria;D_1__Proteobacteria;D_2__Alphaproteobacteria
ee0f5caff94365896ef095e26d1fed6d	194	0.749932806	D_0__Bacteria;D_1__Proteobacteria;D_2__Alphaproteobacteria;D_3__SAR11 clade;D_4__Clade I;D_5__Clade Ia
cb1103feca0f73cffe3e11aa250b31fd	136	0.995767309	D_0__Bacteria;D_1__Proteobacteria;D_2__Gammaproteobacteria
d5533bb4f1f8f99cd6ab0d4513071e63	102	0.846784887	D_0__Bacteria;D_1__Proteobacteria;D_2__Deltaproteobacteria;D_3__Bdellovibrionales;D_4__Bacteriovoracaceae;D_5__Peredibacter;D_6__marine snow associated bacterium Adriatic33
9067ee2adb6a587bc491759a28206ef3	97	0.999879885	D_0__Bacteria;D_1__Proteobacteria;D_2__Gammaproteobacteria
ccb67f3a797ef786dc0c42cf3b8bd220	62	0.999963971	D_0__Bacteria;D_1__Proteobacteria;D_2__Gammaproteobacteria;D_3__Alteromonadales;D_4__Colwelliaceae;D_5__Colwellia
ce42b14845e10ad958ca1cb53045dbca	60	0.995966837	D_0__Bacteria;D_1__Proteobacteria;D_2__Gammaproteobacteria;D_3__Alteromonadales;D_4__Colwelliaceae;D_5__Colwellia
8ad347d406777a81fcd8cfe37ca1c6c0	48	0.999546718	D_0__Bacteria;D_1__Proteobacteria;D_2__Gammaproteobacteria

dfb9078f726f9b239561ae7a04e4c445	42	0.997416652	D_0__Bacteria;D_1__Proteobacteria;D_2__Gammaproteobacteria;D_3__Oceanospirillales;D_4__Nitrincolaceae;D_5__uncultured;D_6__uncultured marine bacterium
09f3ca0e70a9a5172f54c951440f80a1	39	0.999609525	D_0__Bacteria;D_1__Bacteroidetes;D_2__Bacteroidia;D_3__Flavobacteriales;D_4__NS9 marine group;D_5__seawater metagenome;D_6__seawater metagenome
d981719504755a2c4d1d38004b3f25bb	39	0.843412938	D_0__Bacteria;D_1__Proteobacteria;D_2__Gammaproteobacteria;D_3__Thiomicrospirales;D_4__Thioglobaceae;D_5__SUP05 cluster;D_6__uncultured marine bacterium
ece89bff980b08b04d6f8dbe287592c3	39	0.999999069	D_0__Bacteria;D_1__Epsilonbacteraeota;D_2__Campylobacteria;D_3__Campylobacterales;D_4__Arcobacteraceae;D_5__Arcobacter
085f909fd9a9edf1a0bbd3798cf5279b	37	0.887600266	D_0__Bacteria;D_1__Proteobacteria;D_2__Gammaproteobacteria;D_3__Thiomicrospirales;D_4__Thioglobaceae;D_5__SUP05 cluster;D_6__uncultured marine bacterium
1db50f814d8a99be5666486f4a4de024	32	0.999094451	D_0__Bacteria;D_1__Bacteroidetes;D_2__Bacteroidia;D_3__Flavobacteriales;D_4__Flavobacteriaceae;D_5__NS2b marine group
2ed5317eed5cca5f24215fae43088517	29	0.953567109	D_0__Bacteria;D_1__Bacteroidetes;D_2__Bacteroidia;D_3__Flavobacteriales;D_4__Flavobacteriaceae;D_5__[Polaribacter] huanghezhanensis;Ambiguous_taxa
43312cc49398d40c07f6e6122d592887	28	0.991274966	D_0__Archaea;D_1__Thaumarchaeota;D_2__Nitrososphaeria;D_3__Nitrosopumilales;D_4__Nitrosopumilaceae;D_5__Candidatus Nitrosopumilus
6f8cc8a570ce8fcbeeb9ffcbe1d5971a	26	0.999999987	D_0__Bacteria;D_1__Bacteroidetes;D_2__Bacteroidia;D_3__Flavobacteriales;D_4__Flavobacteriaceae;D_5__NS4 marine group
8b1d800c36d922ac85239ca5f576d0ff	26	0.782848771	D_0__Bacteria;D_1__Bacteroidetes;D_2__Bacteroidia;D_3__Flavobacteriales;D_4__NS9 marine group;D_5__uncultured bacterium;D_6__uncultured bacterium
a09fd242d4bbdae1944d1b43ab0a33e9	26	0.926647049	D_0__Bacteria;D_1__Bacteroidetes;D_2__Bacteroidia;D_3__Flavobacteriales;D_4__Flavobacteriaceae;D_5__uncultured;D_6__uncultured marine bacterium
a7bf0266830b194e5ffd0cb22c0effa1	20	0.863165323	D_0__Bacteria;D_1__Bacteroidetes;D_2__Bacteroidia;D_3__Flavobacteriales;D_4__Flavobacteriaceae;D_5__Polaribacter 1;Ambiguous_taxa
ed347145ab935ef912e1f90fe9590298	19	0.886294614	D_0__Bacteria;D_1__Proteobacteria;D_2__Gammaproteobacteria;D_3__Alteromonadales;D_4__Colwelliaceae;D_5__Colwellia
58d4ccc3b5caaca78eb060f7a2d1d9ee	18	1	D_0__Bacteria;D_1__Proteobacteria;D_2__Alphaproteobacteria;D_3__SAR11 clade
7874f80382e597a6eb3bf95a6defd394	17	0.875967844	D_0__Bacteria;D_1__Fusobacteria;D_2__Fusobacteriia;D_3__Fusobacteriales;D_4__Fusobacteriaceae;D_5__Psychrilyobacter;D_6__uncultured bacterium
e007a5a33100efaa889829480150a396	16	0.999999998	D_0__Bacteria;D_1__Planctomycetes;D_2__Phycisphaerae;D_3__Phycisphaerales;D_4__Phycisphaeraceae;D_5__JL-ETNP-F27

e6e4648a60dfe431e5ae2c981eb1ae87	16	0.767008572	D_0__Bacteria;D_1__Spirochaetes;D_2__Leptospirae;D_3__Leptospirales;D_4__Leptospiraceae;D_5__Turneriella;D_6__metagenome
177920551a2a705a66e2f4a2663d4561	15	0.927689543	D_0__Bacteria;D_1__Bacteroidetes;D_2__Bacteroidia;D_3__Flavobacteriales;D_4__Cryomorphaceae;D_5__uncultured;D_6__marine metagenome
51f4a36ffc035b291789656685592f35	15	0.841127864	D_0__Bacteria;D_1__Proteobacteria;D_2__Gammaproteobacteria;D_3__Alteromonadales;D_4__Shewanellaceae;D_5__Ferrimonas;Ambiguous_taxa
71dac9bd043cd476f40ef2d5e7bb28fa	14	0.999999551	D_0__Bacteria;D_1__Proteobacteria;D_2__Gammaproteobacteria;D_3__Alteromonadales;D_4__Psychromonadaceae;D_5__Psychromonas
146d2cd899ac43e7ba5da85a562451c8	13	0.99080369	D_0__Bacteria;D_1__Bacteroidetes;D_2__Bacteroidia;D_3__Flavobacteriales;D_4__Flavobacteriaceae;D_5__Aurantivirga;Ambiguous_taxa
72ab946b81e68afd44dae98c7d4dceb8	13	0.818021979	D_0__Bacteria;D_1__Planctomycetes;D_2__Phycisphaerae;D_3__Phycisphaerales;D_4__Phycisphaeraceae;D_5__FS140-16B-02 marine group;Ambiguous_taxa
0aebc7c8b90e3922ae2960d6bc6ba3f8	12	0.968351558	D_0__Bacteria;D_1__Proteobacteria;D_2__Gammaproteobacteria;D_3__Alteromonadales;D_4__Moritellaceae;D_5__Moritella;Ambiguous_taxa
5ef5daa696583f4b2c8c967c2bc6ac6e	12	0.750595606	D_0__Bacteria;D_1__Proteobacteria;D_2__Gammaproteobacteria;D_3__Cellvibrionales;D_4__Porticoccaceae;D_5__C1-B045;D_6__Cladosiphon okamuranus
a1ab33754f725190a46a5ab09b97314c	12	0.934379771	D_0__Bacteria;D_1__Proteobacteria;D_2__Alphaproteobacteria;D_3__Rhodobacterales;D_4__Rhodobacteraceae;D_5__Planktomarina
c87228fb6f3d84df43335fb48673a6aa	12	0.968020252	D_0__Bacteria;D_1__Bacteroidetes;D_2__Bacteroidia;D_3__Bacteroidales;D_4__Bacteroidaceae;D_5__Bacteroides;D_6__uncultured bacterium
5d6c93c193027c8343ac09042db51049	11	1	D_0__Bacteria;D_1__Planctomycetes;D_2__Phycisphaerae;D_3__Phycisphaerales;D_4__Phycisphaeraceae;D_5__JL-ETNP-F27
04e1d5fe2db1e05d3f7464d881e335db	10	0.999999631	D_0__Bacteria;D_1__Epsilonbacteraeota;D_2__Campylobacteria;D_3__Campylobacterales;D_4__Arcobacteraceae;D_5__Arcobacter
96b6bb9bc81a32759c28a823d202c9c5	10	0.990004753	D_0__Bacteria;D_1__Bacteroidetes;D_2__Bacteroidia;D_3__Flavobacteriales;D_4__Flavobacteriaceae;D_5__NS5 marine group;Ambiguous_taxa
a32ba414e0374b023f41d7a7d80df3a9	10	0.998982167	D_0__Bacteria;D_1__Bacteroidetes;D_2__Bacteroidia;D_3__Flavobacteriales;D_4__Flavobacteriaceae;D_5__Ulvibacter;D_6__uncultured bacterium
2fe8d6838909663a039294cf48ea3720	9	1	D_0__Bacteria;D_1__Firmicutes;D_2__Clostridia;D_3__Clostridiales;D_4__Peptostreptococcaceae;D_5__Peptostreptococcus
35edd74e500829f34672c77fe135a4d8	9	1	D_0__Bacteria;D_1__Bacteroidetes;D_2__Bacteroidia;D_3__Flavobacteriales;D_4__Flavobacteriaceae

b45ad382acdfdda9b261dd9f408b9a41	9	0.891074376	D_0__Bacteria;D_1__Fusobacteria;D_2__Fusobacteriia;D_3__Fusobacteriales;D_4__Fusobacteriaceae;D_5__Cetobacterium;D_6__uncultured bacterium
cc82292bf4dabc6ee88ae87d79c1177f	9	1	D_0__Bacteria;D_1__Proteobacteria;D_2__Alphaproteobacteria;D_3__Rhodobacterales;D_4__Rhodobacteraceae
113ca0d78dfbab0cc1c8b13f7344ffe3	8	0.76008117	D_0__Bacteria;D_1__Proteobacteria;D_2__Gammaproteobacteria;D_3__Cellvibrionales;D_4__Porticoccaceae;D_5__SAR92 clade;D_6__uncultured marine bacterium
197fa7485a53351f83c841134ba5675c	8	0.934418961	D_0__Bacteria;D_1__Bacteroidetes;D_2__Bacteroidia;D_3__Cytophagales;D_4__Cyclobacteriaceae;D_5__Ekhidna;D_6__uncultured bacterium
350073d106270d03bfabb454f3756aa2	8	0.995368165	D_0__Bacteria;D_1__Proteobacteria;D_2__Gammaproteobacteria;D_3__Alteromonadales;D_4__Alteromonadaceae;D_5__Paraglaciecola;Ambiguous_taxa
4071f0397c05488b3cab337f4c7e9bef	8	0.997024978	D_0__Bacteria;D_1__Bacteroidetes;D_2__Bacteroidia;D_3__Flavobacteriales;D_4__Flavobacteriaceae;D_5__Polaribacter 1
8b7d1dd5792eef291d006590ae917a94	8	0.988433233	D_0__Bacteria;D_1__Bacteroidetes;D_2__Bacteroidia;D_3__Flavobacteriales;D_4__Flavobacteriaceae;D_5__NS5 marine group;D_6__uncultured bacterium ARCTIC26_B_07
9073f1da59db31cfe0580916658da539	8	0.99999014	D_0__Bacteria;D_1__Proteobacteria;D_2__Alphaproteobacteria;D_3__SAR11 clade;D_4__Clade IV;Ambiguous_taxa;Ambiguous_taxa
a8a20fd9974fb8fac63672894bb35170	8	0.999756607	D_0__Bacteria;D_1__Bacteroidetes;D_2__Bacteroidia;D_3__Flavobacteriales;D_4__NS9 marine group;D_5__hydrothermal vent metagenome;D_6__hydrothermal vent metagenome
b24b157400490f3df9bf86bb2e09d90b	8	0.992283502	D_0__Bacteria;D_1__Planctomycetes;D_2__Phycisphaerae;D_3__Phycisphaerales;D_4__Phycisphaeraceae;D_5__FS140-16B-02 marine group;D_6__uncultured bacterium
b51ebea7e128a204c4827541f01193fb	8	0.947404044	D_0__Bacteria;D_1__Bacteroidetes;D_2__Bacteroidia;D_3__Bacteroidales;D_4__Bacteroidaceae;D_5__Bacteroides;D_6__uncultured bacterium
b80321b3d96fe17546da5b172528118e	8	0.999891983	D_0__Bacteria;D_1__Proteobacteria;D_2__Gammaproteobacteria;D_3__Oceanospirillales;D_4__Nitrincolaceae;D_5__Profundimonas;Ambiguous_taxa
fa5d7e11432c8e7ab07590c5bd25d55e	8	0.999999999	D_0__Bacteria;D_1__Proteobacteria;D_2__Alphaproteobacteria;D_3__Rhodobacterales;D_4__Rhodobacteraceae
2d1527fdf206a9dbf8f3702b90d68ce8	7	0.753968916	D_0__Bacteria;D_1__Spirochaetes;D_2__Leptospirae;D_3__Leptospirales;D_4__Leptospiraceae;D_5__uncultured
3ff67ff349463e155e421199e745409b	7	0.999999997	D_0__Bacteria;D_1__Planctomycetes;D_2__Planctomycetacia;D_3__Pirellulales;D_4__Pirellulaceae;D_5__Blastopirellula
43adac1596ede1dd88ea74502011a729	7	0.999306936	D_0__Bacteria;D_1__Proteobacteria;D_2__Gammaproteobacteria;D_3__Steroidobacterales;D_4__Woeseiaceae;D_5__Woeseia;D_6__uncultured marine bacterium

79762d2abcbd928b75e0b39c675d5424	7	0.904765981	D_0__Bacteria;D_1__Bacteroidetes;D_2__Bacteroidia;D_3__Bacteroidales;D_4__Rikenellaceae;D_5__Alistipes;D_6__uncultured bacterium
9390711f3044e7aba9cb577a0aaaf34e	7	0.999999983	D_0__Bacteria;D_1__Bacteroidetes;D_2__Bacteroidia;D_3__Flavobacteriales;D_4__Flavobacteriaceae;D_5__Pseudofulvibacter
b2ec1271704b44ad94883257227b11df	7	0.876150587	D_0__Bacteria;D_1__Proteobacteria;D_2__Gammaproteobacteria;D_3__Cellvibrionales;D_4__Porticoccaceae;D_5__C1-B045;D_6__Cladosiphon okamuranus
d942bac17b1a69d8499fc1ac7c8b4dac	7	0.984858379	D_0__Bacteria;D_1__Bacteroidetes;D_2__Bacteroidia;D_3__Bacteroidales;D_4__Marinifilaceae;D_5__Marinifilum;D_6__uncultured Bacteroidetes bacterium
e147fdb14fbbbdd2b7e9f974c42603e0	7	0.999996332	D_0__Bacteria;D_1__Proteobacteria;D_2__Gammaproteobacteria;D_3__Alteromonadales;D_4__Shewanellaceae;D_5__Psychrobium
e1b000731bf1b4ab30332e90d5da12fd	7	0.984201195	D_0__Bacteria;D_1__Bacteroidetes;D_2__Bacteroidia;D_3__Bacteroidales;D_4__Rikenellaceae;D_5__Alistipes;D_6__uncultured bacterium
00a80fb2e5b6d10a531c341927651e92	6	0.75514023	D_0__Bacteria;D_1__Proteobacteria;D_2__Alphaproteobacteria;D_3__Rhodobacterales;D_4__Rhodobacteraceae;D_5__Roseobacter clade NAC11-7 lineage;D_6__uncultured alpha proteobacterium
12d1684808f0df24b75a71c8bf8ccd56	6	0.99975945	D_0__Bacteria;D_1__Proteobacteria;D_2__Gammaproteobacteria;D_3__Cellvibrionales;D_4__Halieaceae;D_5__OM60(NOR5) clade
443ec713433d2a2beece7932060554c0	6	0.999999985	D_0__Bacteria;D_1__Epsilonbacteraeota;D_2__Campylobacteria;D_3__Campylobacterales;D_4__Arcobacteraceae;D_5__Arcobacter
4da211ba6ea0339b60009f074cc4d0e0	6	0.783727801	D_0__Bacteria;D_1__Actinobacteria;D_2__Coriobacteriia;D_3__Coriobacteriales;D_4__Atopobiaceae;D_5__Atopobium;D_6__uncultured Coriobacteriaceae bacterium
60a8eb1607d40d20a96caadce2d139c6	6	0.992260838	D_0__Bacteria;D_1__Proteobacteria;D_2__Gammaproteobacteria;D_3__Oceanospirillales;D_4__Pseudohongiellaceae;D_5__Pseudohongiella;D_6__uncultured marine bacterium
63a8d5a3a8791f53fae465b038315d2a	6	0.93554543	D_0__Bacteria;D_1__Bacteroidetes;D_2__Bacteroidia;D_3__Bacteroidales;D_4__Marinifilaceae;D_5__uncultured;Ambiguous_taxa
a318c488bbc906c5166e934ea935ddd6	6	0.856710524	D_0__Bacteria;D_1__Nitrospinae;D_2__Nitrospinia;D_3__Nitrospinales;D_4__Nitrospinaceae;D_5__LS-NOB;D_6__uncultured delta proteobacterium
abdbe9456e55bfa79d5ec2ef030deb43	6	0.997785995	D_0__Bacteria;D_1__Patescibacteria;D_2__Gracilibacteria;D_3__uncultured bacterium;D_4__uncultured bacterium;D_5__uncultured bacterium;D_6__uncultured bacterium
e249ebb7b48c0afd3c421d36e989c87a	6	0.992511423	D_0__Bacteria;D_1__Planctomycetes;D_2__Phycisphaerae;D_3__Phycisphaerales;D_4__Phycisphaeraceae;D_5__CL500-3;Ambiguous_taxa
3f9aab3f978fca06b2e018c352a72b97	5	0.845134855	D_0__Bacteria;D_1__Proteobacteria;D_2__Gammaproteobacteria;D_3__Betaproteobacteriales;D_4__Methylophilaceae;D_5__OM43 clade;D_6__uncultured organism

4fea4c7f3485c1c417cdd94a20b1271f	5	0.999791177	D_0__Bacteria;D_1__Proteobacteria;D_2__Alphaproteobacteria;D_3__Parvibaculales;D_4__Parvibaculaceae;D_5__uncultured;Ambiguous_taxa
5bddb82944e47ce930542b6cc2cec5bb	5	0.936341911	D_0__Bacteria;D_1__Proteobacteria;D_2__Alphaproteobacteria;D_3__Puniceispirillales;D_4__SAR116 clade;D_5__uncultured marine bacterium;D_6__uncultured marine bacterium
67bd572773f3d10297941e4d5aa8fbda	5	0.999919553	D_0__Bacteria;D_1__Marinimicrobia (SAR406 clade);D_2__uncultured marine bacterium;D_3__uncultured marine bacterium;D_4__uncultured marine bacterium;D_5__uncultured marine bacterium;D_6__uncultured marine bacterium
690b115eebd20dbe59a39553854f51f9	5	0.999975583	D_0__Bacteria;D_1__Proteobacteria;D_2__Alphaproteobacteria;D_3__uncultured
7b7afb455fc96b0b2aa4a17f9a5dbd0a	5	0.923579305	D_0__Bacteria;D_1__Bacteroidetes;D_2__Bacteroidia;D_3__Flavobacteriales;D_4__NS9 marine group;D_5__uncultured marine bacterium;D_6__uncultured marine bacterium
a7e09914eeb30a7fbd752cd174474a55	5	0.998192618	D_0__Bacteria;D_1__Verrucomicrobia;D_2__Verrucomicrobiae;D_3__Verrucomicrobiales;D_4__Rubritaleaceae;D_5__Rubritalea;D_6__uncultured bacterium
ba99a570c2761e419c443a97c0ea39d3	5	0.999999975	D_0__Bacteria;D_1__Epsilonbacteraeota;D_2__Campylobacteria;D_3__Campylobacterales;D_4__Arcobacteraceae;D_5__Arcobacter
fed8160f587c4ce15fde645541293117	5	0.999897816	D_0__Bacteria;D_1__Nitrospinae;D_2__Nitrospinia;D_3__Nitrospinales;D_4__Nitrospinaceae;D_5__Nitrospina
0806215b5b0ea280961ac91fee9cffce	4	0.999999555	D_0__Bacteria;D_1__Proteobacteria;D_2__Gammaproteobacteria;D_3__SAR86 clade
0ab95cab67a77cfdddcc279c77735436	4	0.997926476	D_0__Bacteria;D_1__Planctomycetes;D_2__Planctomycetacia;D_3__Pirellulales;D_4__Pirellulaceae;D_5__Rhodopirellula;D_6__marine metagenome
2e1b6e797697e572ca82dd224d99d79b	4	0.999998257	D_0__Bacteria;D_1__Bacteroidetes;D_2__Bacteroidia;D_3__Bacteroidales;D_4__Marinifilaceae;D_5__Marinifilum
7a4321e429e11674a5ef2c41c8619231	4	0.998746714	D_0__Bacteria;D_1__Bacteroidetes;D_2__Bacteroidia;D_3__Chitinophagales;D_4__uncultured;D_5__bacterium episymbiont of Kiwa sp.;D_6__bacterium episymbiont of Kiwa sp.
cc1a20baae9c705d4ce4ddc21cd48ecb	4	0.847305238	D_0__Bacteria;D_1__Firmicutes;D_2__Clostridia;D_3__Clostridiales;D_4__Ruminococcaceae;D_5__Butyricicoccus;Ambiguous_taxa
ea6d70a3073a2631241629b5be1a12ef	4	0.99880395	D_0__Bacteria;D_1__Firmicutes;D_2__Clostridia;D_3__Clostridiales;D_4__Peptostreptococcaceae;D_5__Paeniclostridium
1721390e92c8ec94f680613d2abfa4e5	3	0.999999968	D_0__Bacteria;D_1__Planctomycetes;D_2__Phycisphaerae;D_3__Phycisphaerales;D_4__Phycisphaeraceae;D_5__JL-ETNP-F27;Ambiguous_taxa
47a798da9c732214d382228868734180	3	0.999999734	D_0__Bacteria;D_1__Dadabacteria;D_2__Dadabacteriia;D_3__Dadabacteriales;D_4__metagenome;D_5__metagenome;D_6__metagenome

80c2824714f43afc7d5cf2bfd7fe87c1	3	0.90390555	D_0__Bacteria;D_1__Bacteroidetes;D_2__Bacteroidia;D_3__Flavobacteriales;D_4__Crocinitomicaceae;D_5__Crocinitomix;Ambiguous_taxa
8c68ef866ea38f62b8dfcff1c4046a17	3	0.982985514	D_0__Bacteria;D_1__Proteobacteria;D_2__Deltaproteobacteria;D_3__SAR324 clade(Marine group B);D_4__uncultured bacterium;D_5__uncultured bacterium;D_6__uncultured bacterium
9791a91a6d944850f4b9a785ed666a8b	3	0.830231704	D_0__Bacteria;D_1__Marinimicrobia (SAR406 clade);D_2__uncultured marine bacterium;D_3__uncultured marine bacterium;D_4__uncultured marine bacterium;D_5__uncultured marine bacterium;D_6__uncultured marine bacterium
98a71c6554e70951ba750ec1252c270e	3	0.999851021	D_0__Bacteria;D_1__Proteobacteria;D_2__Gammaproteobacteria;D_3__SAR86 clade;Ambiguous_taxa;Ambiguous_taxa;Ambiguous_taxa
9e4bb52a30262dd18e9cc6ee7a51b08f	3	0.943042595	D_0__Bacteria;D_1__Proteobacteria;D_2__Alphaproteobacteria;D_3__Micavibrionales;D_4__Micavibrionaceae;D_5__uncultured;D_6__uncultured bacterium
bfec995109ecdb78c9407f9673703274	3	0.999999999	D_0__Bacteria;D_1__Proteobacteria;D_2__Gammaproteobacteria;D_3__SAR86 clade
d0b7d52b3cf4151668dcf5eb892d0621	3	0.999929109	D_0__Bacteria;D_1__Proteobacteria;D_2__Gammaproteobacteria;D_3__Oceanospirillales;D_4__Nitrincolaceae;D_5__Profundimonas;Ambiguous_taxa
e6e0465de143c5fa2665b96e0fc6efa0	3	0.998613306	D_0__Bacteria;D_1__Bacteroidetes;D_2__Bacteroidia;D_3__Cytophagales;D_4__Cyclobacteriaceae;D_5__Marinoscillum;Ambiguous_taxa
143feb115e9ca370475882e79107400a	2	0.999884546	D_0__Bacteria;D_1__Proteobacteria;D_2__Gammaproteobacteria;D_3__Betaproteobacteriales
1494b3f147593c4487c77bf81ad1b72f	2	0.999177113	D_0__Bacteria;D_1__Verrucomicrobia;D_2__Verrucomicrobiae;D_3__Opitutales;D_4__Puniceicoccaceae;D_5__MB11C04 marine group;Ambiguous_taxa
16a17b6bff10137f8e1a022d6202656a	2	0.955923338	D_0__Bacteria;D_1__Bacteroidetes;D_2__Bacteroidia;D_3__Chitinophagales;D_4__Saprospiraceae;D_5__uncultured;D_6__uncultured bacterium
64aa0a70eb77e52893e8f6b763ec3912	2	0.93062016	D_0__Bacteria;D_1__Planctomycetes;D_2__OM190;Ambiguous_taxa;Ambiguous_taxa;Ambiguous_taxa;Ambiguous_taxa
67c9e284c7de43eb5596d0b6434a57f8	2	0.759345136	D_0__Bacteria;D_1__Bacteroidetes;D_2__Bacteroidia;D_3__Bacteroidales;D_4__Prevotellaceae;D_5__Alloprevotella;D_6__gut metagenome
7597f9affb71896463d206286537c96f	2	0.962043014	D_0__Bacteria;D_1__Firmicutes;D_2__Erysipelotrichia;D_3__Erysipelotrichales;D_4__Erysipelotrichaceae;D_5__Erysipelatoclostridium;Ambiguous_taxa
9a1187e6d6390c1c499f5fcdac7409dd	2	0.955580637	D_0__Bacteria;D_1__Chlamydiae;D_2__Chlamydiae;D_3__Chlamydiales
9cae1a3205516123b356969fdf0ae6a3	2	0.767597456	D_0__Bacteria;D_1__Firmicutes;D_2__Clostridia;D_3__Clostridiales;D_4__Ruminococcaceae;D_5__[Eubacterium] coprostanoligenes group;D_6__uncultured bacterium

a017e5656bed86b3ce4efc4ae0afa01b	2	0.999997973	D_0__Bacteria;D_1__Epsilonbacteraeota;D_2__Campylobacteria;D_3__Campylobacterales;D_4__Campylobacteraceae;D_5__Campylobacter
d736a7f6a5f0457fe08269e4604c3852	2	0.993864882	D_0__Bacteria;D_1__Tenericutes;D_2__Mollicutes;D_3__Mycoplasmatales;D_4__Mycoplasmataceae;D_5__Ureaplasma;D_6__gut metagenome
ee78aacb586424536d6150fd3975c5d7	2	0.999997059	D_0__Bacteria;D_1__Proteobacteria;D_2__Deltaproteobacteria;D_3__Desulfarculales;D_4__Desulfarculaceae;D_5__uncultured;Ambiguous_taxa
267fcc354b6397d0441b08c2274f6287	1	0.917729639	D_0__Bacteria;D_1__Proteobacteria;D_2__Gammaproteobacteria;D_3__Oceanospirillales
36ba763d09fcb5edf46f04298cc640c0	1	0.782141413	D_0__Bacteria;D_1__Proteobacteria;D_2__Alphaproteobacteria;D_3__Kordiimonadales;D_4__uncultured
52ecbaefad84d6219c14f865457a431a	1	0.938640681	D_0__Bacteria;D_1__Proteobacteria;D_2__Gammaproteobacteria;D_3__UBA10353 marine group
b834fc0909937d337ac9a5d1423a37e4	1	0.973737587	D_0__Bacteria;D_1__Proteobacteria;D_2__Gammaproteobacteria;D_3__Oceanospirillales;D_4__Endozoicomonadaceae;D_5__Endozoicomonas;D_6__uncultured marine bacterium
dd9833fecbec2837598dc450e08febeb	1	0.873543141	D_0__Bacteria;D_1__Proteobacteria;D_2__Gammaproteobacteria;D_3__Betaproteobacteriales
ebc0b6e340bcfca0ecd2c7868bbae828	1	0.912956972	D_0__Bacteria;D_1__Verrucomicrobia;D_2__Verrucomicrobiae;D_3__Opitutales;D_4__Puniceicoccaceae;D_5__Pelagicoccus;D_6__uncultured gamma proteobacterium

Table S9. Targeted region, forward and reverse names, sequences (5'→ 3') and reference of primers pairs used for molecular characterization.

Targeted region	Forward Name	Sequence (5'→3')	Reverse Name	Sequence (5'→3')	Reference
18S	A	AACCTGGTTGATC CTGCCAGT	B	TGATCCTTCTGCAGGTT CACCTAC	Medlin, 1988; Schmitt et al., 2005
18S	18S-AF	CTGGTTGATCCTGCCAG	18S-BR	CTGCAGGTTCACCTAC	Dohrmann et al., 2008; Collins, 2002
18S	18S1	AACCTGGTTGATCCTGCCA	18S2	TGCAGGTTCACCTACAGAA	Manuel et al., 2003
ITS	RA2	GTCCCTGCCCTTTGT ACACA	ITS2.2	CCTGGTTAGTTTCTTTTCCTCC GC	Wörheide et al., 2002a, b; Schmitt et al., 2005
28S	NL4F	GACCCGAAAGATGGTGAACTA	NL4R	ACCTTGGAGACCTGATGCG	Dohrmann et al., 2008; Collins, 2002
28S	C2	GAAAAGAACTTTGRARAGAGAGT	D2	TCCGTGTTTCAAGACGGG	Chombard et al., 1998
CO1	dgLCO1490	GGTCAACAAATCATAAAGAYATYGG	dgHCO2198	TAAACTTCAGGGTGACCAAARAAYCA	Meyer et al., 2005
CO1	PorCOI2fwd	AATATGNGGGCNCCNGGNATNAC	PorCOI2rev	ACTGCCCCCATNGATAAAACAT	Pons et al., 2017

Table S10. Denoising process.

Sample ID	Input	Filtered	Input passed filter (%)	Denoised	Merged	Input merged (%)	Non-chimeric	Input non-chimeric (%)
B4	22476	22309	99.26	20279	16985	75.57	11647	51.82
C6	14741	14711	99.8	14148	12878	87.36	9108	61.79
D4	23248	23191	99.75	22582	20948	90.11	13898	59.78
D6	18168	18095	99.6	17240	16007	88.11	13212	72.72
